# Shedding X-ray Light on the Role of Magnesium in the Activity of *Mycobacterium tuberculosis* Salicylate Synthase (MbtI) for Drug Design

**DOI:** 10.1021/acs.jmedchem.0c00373

**Published:** 2020-06-12

**Authors:** Matteo Mori, Giovanni Stelitano, Arianna Gelain, Elena Pini, Laurent R. Chiarelli, José C. Sammartino, Giulio Poli, Tiziano Tuccinardi, Giangiacomo Beretta, Alessio Porta, Marco Bellinzoni, Stefania Villa, Fiorella Meneghetti

**Affiliations:** † Dipartimento di Scienze Farmaceutiche, Università degli Studi di Milano, via L. Mangiagalli 25, 20133 Milano,Italy; ‡ Dipartimento di Biologia e Biotecnologie “Lazzaro Spallanzani”, Università degli Studi di Pavia, via A. Ferrata 9, 27100 Pavia, Italy; § Dipartimento di Farmacia, Università di Pisa, via Bonanno Pisano 6, 56126 Pisa, Italy; ∥ Sbarro Institute for Cancer Research and Molecular Medicine, Center for Biotechnology, College of Science and Technology, Temple University, Philadelphia, Pennsylvania 19122, United States; ⊥ Dipartimento di Scienze e Politiche Ambientali, Università degli Studi di Milano, via G. Celoria 2, 20133 Milano, Italy; # Dipartimento di Chimica, Università degli Studi di Pavia, via T. Taramelli 12, 27100 Pavia, Italy; ¶ Unité de Microbiologie Structurale, Institut Pasteur, CNRS, Université de Paris, F-75015 Paris, France

## Abstract

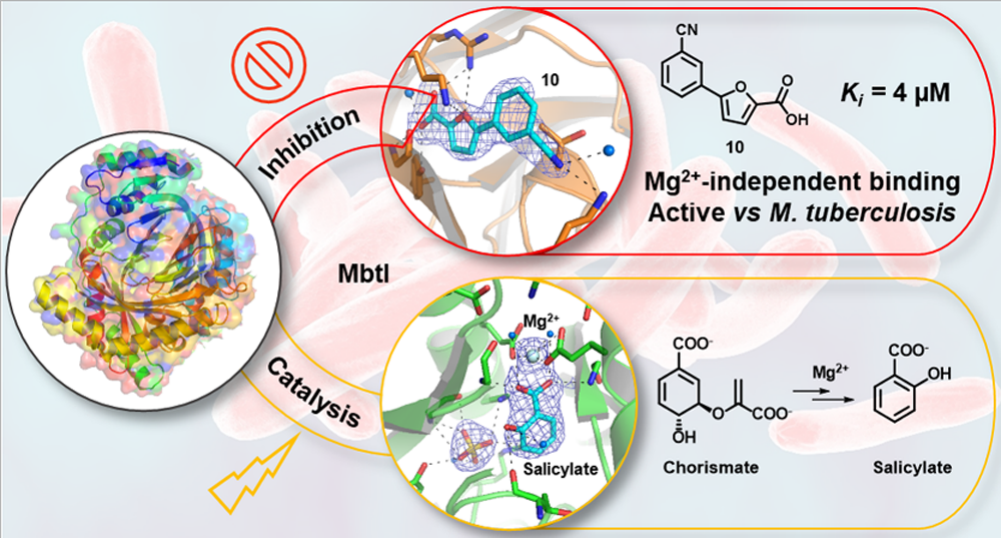

The Mg^2+^-dependent *Mycobacterium tuberculosis* salicylate synthase (MbtI) is a key enzyme involved in the biosynthesis of siderophores. Because iron is essential for the survival and pathogenicity of the microorganism, this protein constitutes an attractive target for antitubercular therapy, also considering the absence of homologous enzymes in mammals. An extension of the structure–activity relationships of our furan-based candidates allowed us to disclose the most potent competitive inhibitor known to date (**10**, *K*_i_ = 4 μM), which also proved effective on mycobacterial cultures. By structural studies, we characterized its unexpected Mg^2+^-independent binding mode. We also investigated the role of the Mg^2+^ cofactor in catalysis, analyzing the first crystal structure of the MbtI–Mg^2+^–salicylate ternary complex. Overall, these results pave the way for the development of novel antituberculars through the rational design of improved MbtI inhibitors.

## Introduction

Nowadays, tuberculosis (TB) ranks among the top ten causes of death worldwide; therefore, the development of new scaffolds is imperative to sustain the drug pipeline, considering the issues of the available antitubercular therapies and the increasing emergence of resistant infections.^1^ The salicylate synthase MbtI from *Mycobacterium tuberculosis* (Mtb, the etiological agent of TB) is the first enzyme involved in the biosynthesis of mycobactins. These small-molecule siderophores are capable of chelating iron, a key cofactor involved in several mycobacterium-specific biological processes. MbtI has been structurally and biochemically characterized;^2^ it is essential for the survival of Mtb under iron-deficient conditions,^3^ and, as such, it has been identified as a promising therapeutic target.^4^

**Figure 1:**
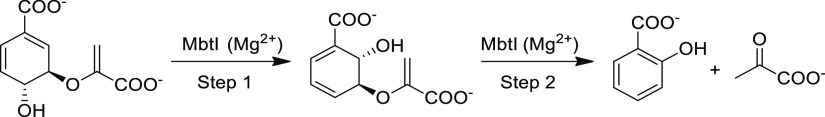
Reactions catalyzed by MbtI.

MbtI belongs to the group of structurally homologous Mg^2+^-dependent menaquinone, siderophore, and tryptophan (MST) enzymes, which transform chorismate by rearrangement, to generate precursor molecules for the respective biosynthetic pathways.^5,6^ In this context, MbtI catalyzes the two-step conversion of chorismate to salicylate, *via* isochorismate as an intermediate, performing an isomerase activity followed by a lyase activity (Figure 1).^4^

Several strategies have been adopted for the discovery of MbtI inhibitors. In particular, dicarboxylate substrate analogues (chorismate and isochorismate), transition state analogues, and compounds originated from high-throughput screening (HTS) have been identified.^7−10^ However, these compounds, albeit active against the isolated enzyme, show modest effects on the whole mycobacterial cell.^7−10^ Notably, methyl-AMT (IC_50_ = 11.6 μM), the best MbtI inhibitor discovered to date not belonging to our furan-based class, exhibited a poor minimum inhibitory concentration (MIC^50^ ≥ 1 mM), which remained weak (MIC^50^ = 792 μM) for its more lipophilic dimethyl ester analogue as well.^4^

**Figure 2:**
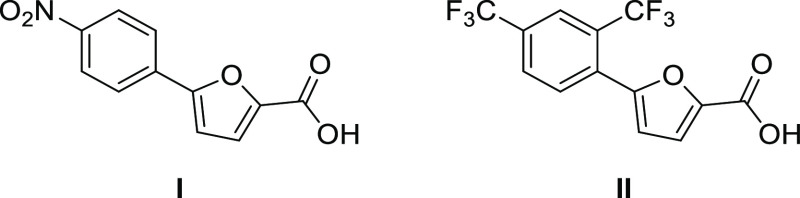
Chemical structures of compounds **I** and **II**.

Therefore, in order to find improved MbtI inhibitors, we followed an *in silico* screening approach, which led to the identification of a new hit.^11^ While exploring the chemical space around this compound in the frame of a thorough structure–activity relationship (SAR) study, we discovered two effective derivatives, **I** and **II** (Figure 2), which are, to our knowledge, the most potent MbtI inhibitors reported to date (IC_50_ = 7.6 ± 1.6 μM and 13.1 ± 2.0 μM, respectively). This strong inhibition of MbtI is correlated with a lethal effect on Mtb cultures (MIC^99^ = 156 and 250 μM, respectively).^11,12^ Furthermore, the Universal chrome azurol S (CAS) assay confirmed the connection between the antimycobacterial effect of these compounds and the disruption of mycobactin biosynthesis, thus highlighting the importance of this pathway as a target for the development of therapeutic interventions. Moreover, we demonstrated the possibility of removing one of the carboxylic groups of methyl-AMT without causing a loss in the inhibitory potency and allowing, at the same time, a better permeability through the cell wall of Mtb.^11,12^

Despite the promising results obtained in the inhibition of this target,^4^ its mechanism of action is still poorly understood and not supported by conclusive experimental data. In particular, a robust definition of the role of Mg^2+^ in the interaction between MbtI and inhibitors is still lacking. It has been reported that the affinity of the MST enzymes EntC, PchA, and Irp9 for ligands is much higher than that for the metal ion, so the driving element of the catalytic reaction is the binding of the substrate, followed by interaction with the cofactor.^6^ After the conversion of chorismate to isochorismate, a sudden change in the affinity for the metal occurs: Mg^2+^ is retained by MST enzymes with an extremely high affinity, promoting the subsequent reaction which quickly leads to the formation of salicylate.^6^

Stimulated by these findings, we started to consider if **I** and **II** may participate in the inhibition mechanism by occupying the active site of the free MbtI before the intervention of the Mg^2+^ ion, thus blocking the isomerase activity. In this work, we provide experimental data to explain the role of Mg^2+^ on the activity and inhibition of MbtI. First, we expanded our library of furan-based compounds, designing and synthesizing new meta-derivatives. Among them, the most potent inhibitor identified so far (the *m*-cyano compound **10**, Table 1) was selected as the most suitable candidate to deepen our investigations. The determination of its activity, performed at increasing concentrations of Mg^2+^, showed that the metal did not influence the binding of the compound to the target. Then, we updated our computational model, taking into account the Mg^2+^-independent binding mode, which allowed us to identify a previously unconsidered key role of some residues. This hypothetical pose was supported by the obtainment of the crystallographic complex of MbtI with **10**, which is described here. As a proof of principle, we also solved a crystal structure of the enzyme in complex with Mg^2+^, obtained at saturating concentrations of the metal. Moreover, the MbtI–Mg^2+^ crystal structure evidenced the presence of salicylate, the product of the enzymatic reaction. Notably, the presence and the interaction pattern of salicylate at the active site confirmed the hypothesized catalytic mechanism of MbtI, previously inferred by similarity to other MST enzymes.

**Table 1:**
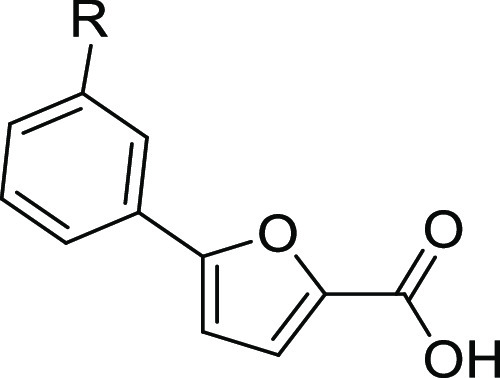
m-Substituted Derivatives **1–10**^a^

code	*R*	% residual activity	IC_50_ (μM)
**1**	3-CF_3_	42.0 ± 6.3	
**2**	3-Cl	101.6 ± 17.8	
**3**	3-OH	70.4 ± 21.8	
**4**	3-CH_3_	103.9 ± 4.8	
**5**	3-NH_2_	65.8 ± 9.6	
**6**	3-CONH_2_	20.9 ± 4.3	31.4 ± 10.3
**7**	3-CONHCH_3_	84.0 ± 9.1	
**8**	3-SO_2_NH_2_	28.6 ± 6.8	
**9**	3-COOH	27.2 ± 4.5	
**10**	3-CN	3.1 ± 1.0	6.3 ± 0.9

a Inhibitory effect is expressed as percentage of residual enzymatic activity (at 100 μM ligand concentration) for all compounds and half-maximal inhibitory concentrations (IC_50_, μM) only for the most active candidates (residual activity ≤ 25%).

## Results and Discussion

### Synthesis and SAR of Compounds **1–10**

Inspired by the encouraging activity of our leads **I** and **II**,^11,12^ we decided to further explore the phenyl-furan scaffold. In particular, with the aim of obtaining additional SAR data, we focused on exploring the meta position of the phenyl ring using substituents endowed with different stereoelectronic properties, many of which were already employed in our previous studies in which the ortho and para positions of the phenyl ring were mainly substituted. Compound **1**, bearing only one CF_3_ at position 3, was purchased from a commercial source, while **2–10** were synthesized.

**Scheme 1:**
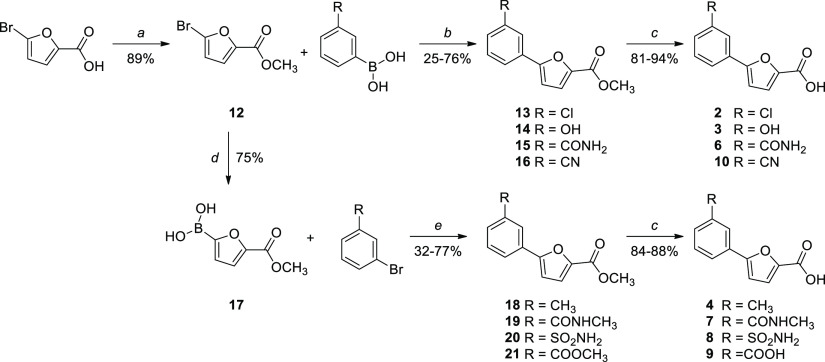
Synthesis of Compounds **2–4** and **6–10** Reagents and conditions: (a) conc. H_2_SO_4_, MeOH, reflux, 24 h; (b) Pd(PPh_3_)_2_Cl_2_, 2 M Na_2_CO_3_, 1,4-dioxane, 90 °C, overnight, N_2_; (c) NaOH, EtOH/THF 1:1, reflux, 5 h or LiOH·H_2_O, THF/H_2_O 1:1, 20 °C, 2 h. (d) (1) Bis[2-(*N*,*N*-dimethylamino)ethyl] ether, 2 M *i*-PrMgCl, THF, 20 min 10–15 °C–30 min r.t., N_2_; (2) B(OCH_3_)_3_, 0 °C, 10 min, N_2_; (e) Pd(PPh_3_)_2_Cl_2_, 2 M Na_2_CO_3_, 1,4-dioxane, 60 °C, 90 min, MW, N_2_.

Compounds **2–4** and **6–10** were synthesized by a Suzuki–Miyaura reaction,^11^ either between methyl 5-bromofuran-2-carboxylate and the suitable boronic acid or between (5-(methoxycarbonyl)furan-2-yl)boronic acid and the appropriate bromo-derivative. The so-obtained esters were hydrolyzed in basic-conditions to yield the free carboxylic acids (Scheme 1). Finally, compound **5** was synthesized from 5-(3-nitrophenyl)furan-2-carboxylate (**23**) through the reduction of its nitro group with tin(II) chloride,^13^ followed by the hydrolysis of the ester function (see Scheme S1 in Supporting Information).

The activity of compounds **1–10** was tested against recombinant MbtI (Table 1), which was prepared and assayed as previously reported.^12^ Compound **1** showed a promising activity, while the replacement with a halogen (**2**) or with electron-donating functions such as the hydroxyl (**3**), methyl (**4**), or amino (**5**) groups abolished or significantly weakened the activity. The insertion of a strong electron-withdrawing group capable of forming a localized negative charge such as the NO_2_ function, previously published (inhibitory activity: ∼50%, at a concentration of 100 μM),^11^ did not lead to an improvement of the activity. Conversely, the amide group of **6** allowed for a significant enhancement of the inhibition, as shown by the IC_50_ value of 31 μM. While the methylation of the amide in compound **7** led to a strong decrement of the activity, the substitution of the amide with classical bioisosteres (sulfonamide, **8**; carboxylic acid, **9**) allowed for a retention of the inhibitory effect. Interestingly, the substitution with a nitrile in **10** afforded the best compound of the series, characterized by an IC_50_ value of about 6 μM; this inhibitor proved to be slightly better than the previous candidates **I** and **II**, thus becoming our improved lead compound.

### Biological Assays

**Figure 3:**
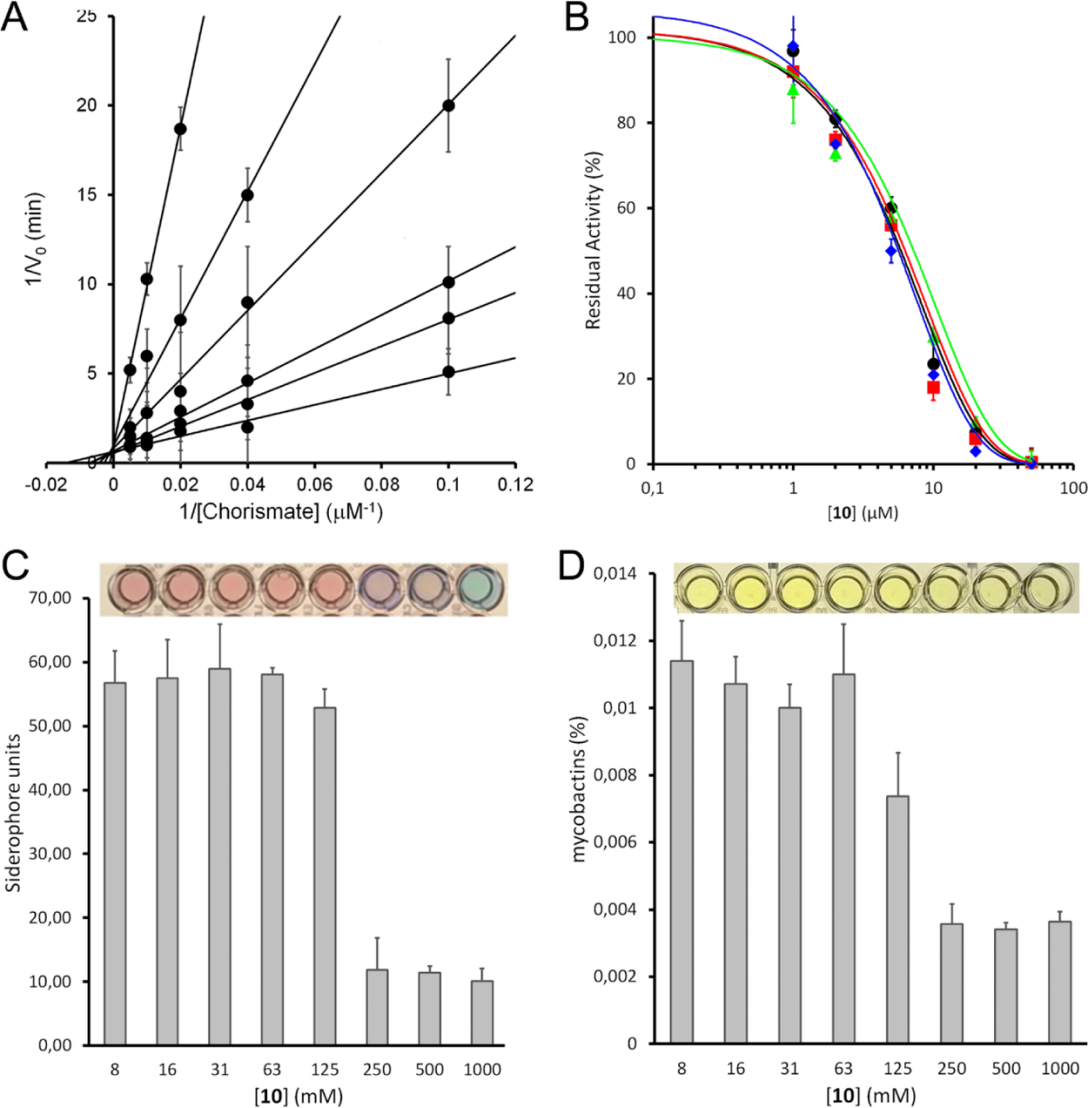
Global reciprocal plot of data from MbtI steady-state kinetics analysis toward chorismic acid at different concentrations of **10** (A). IC_50_ plot of **10** in the presence of BSA (red), Triton X-100 (green), and DTT (blue) (B). Universal CAS assay performed on *M. bovis* BCG, grown at different concentrations of **10** (C). Determination of mycobactins in the abovementioned cells. Bars represent mean and standard deviations of three independent experiments (D).

Compound **10** underwent an in-depth biological evaluation (Figure 3), aimed at characterizing its activity on MbtI and confirming the correlation with a lethal effect on the whole mycobacterial cell. A kinetic analysis confirmed **10** as a competitive inhibitor of MbtI, with a *K*_i_ value of 4.2 ± 0.8 μM (Figure 3A). Additional tests were performed to ensure that it was not a pan-assay interference compound (PAIN):^14^ the addition of bovine serum albumin (BSA) and Triton X-100 did not influence the IC_50_ (6.1 ± 0.9 and 5.8 ± 1.2 μM, respectively), suggesting that it does not form aggregates with the target. Similarly, the addition of 1,4-dithio-dl-threitol (DTT) did not have an impact on the activity of **10** (IC_50_ 7.2 ± 1.1 μM), showing that the ligand does not interact with the cysteine residues of the protein (Figure 3B). The antimycobacterial activity of **10** was tested on *M. tuberculosis* H37Rv, providing an MIC^99^ value (250 μM) similar to those of the previous inhibitors **I** and **II**.^11^ Moreover, to ascertain that the effects of the compound were due to mycobactin inhibition, **10** was assayed against the non-pathogenic *Mycobacterium bovis* bacillus Calmette–Guerin (BCG) strain, whose siderophores closely resemble Mtb mycobactins,^15^ in iron-limiting conditions, using the chelated Sauton’s medium. The compound showed an MIC^99^ value of 250 μM, and the Universal CAS liquid assay and quantification of the mycobactins^16^ in treated cultures demonstrated that siderophore concentration decreased at higher concentrations of the compound (Figure 3C,D). This observation confirmed that the inhibitory effect toward mycobacterial growth was due to mycobactin biosynthesis inhibition. Finally, **10** was screened, following the previously published procedure,^12^ against human normal human fetal lung fibroblast cell line (MRC-5) fibroblasts to evaluate its cytotoxicity, revealing an IC_50_ > 100 μM, thus indicating a low level of toxicity.

### Investigation of the Mg^2+^ Ion

Meneely et al.^6^ demonstrated that, in iron-limiting conditions, the affinity of the MST enzymes EntC, PchA and Irp9 for Mg^2+^ is rather low; hence, the ligand is the first driving element of the catalytic reaction. Subsequently, the binding of the Mg^2+^ caps the active site, thus promoting the initiation of the biosynthetic process. After the conversion of chorismate to isochorismate, a sudden change in the affinity for the metal occurs: the enzyme tightly binds Mg^2+^, preventing the release of the intermediate and allowing the reaction to quickly evolve toward the formation of salicylate.^6^

**Figure 4:**
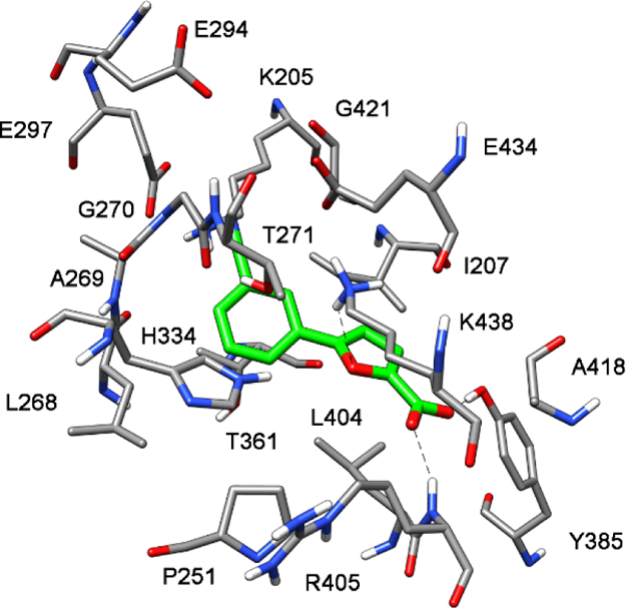
Minimized average structure of **10** within the MbtI binding site (PDB ID 3VEH) in the absence of the Mg^2+^ ion.

According to this theory, a ligand endowed with inhibitory properties should therefore be able to bind to the active site of an MST enzyme, without having to interact with or, much less, be oriented by the Mg^2+^ ion. On these bases, we undertook an investigation to verify if such a deduction could be applied to MbtI and to our compounds. Considering that the presence of Mg^2+^ has been one of the assumptions of most computational studies regarding MbtI, including ours, we realized that unraveling the binding mechanism of our inhibitors would have a particular significance. In order to ascertain the likelihood of a Mg^2+^-independent binding mode for our compounds, docking experiments, combined with molecular dynamics (MD) simulations, were carried out. The predicted dispositions of **10** into the catalytic site of MbtI was calculated by docking in the absence of the Mg^2+^ ion and then refined through 100 ns of MD simulation with explicit water molecules (see Experimental Section for details). The results of these studies are shown in Figure 4, which represents the minimized average structures of **10** within the MbtI binding site. The carboxylic group of the ligand shows interactions with the hydroxyl group of Tyr385 and the backbone nitrogen of Arg405. The furan oxygen forms a H-bond with Lys438, whereas the benzonitrile fragment shows lipophilic interactions with Ile207, Leu268, and Thr361 and an H-bond with Lys205.

Hence, our modeling approach showed that a Mg^2+^-independent binding mode was indeed possible, although the exact pose of the ligand could hardly be predicted by a docking/MD simulation study. Therefore, further experimental studies were carried out to characterize the binding mode of our inhibitor.

### Biochemical Investigations on **10**

**Figure 5:**
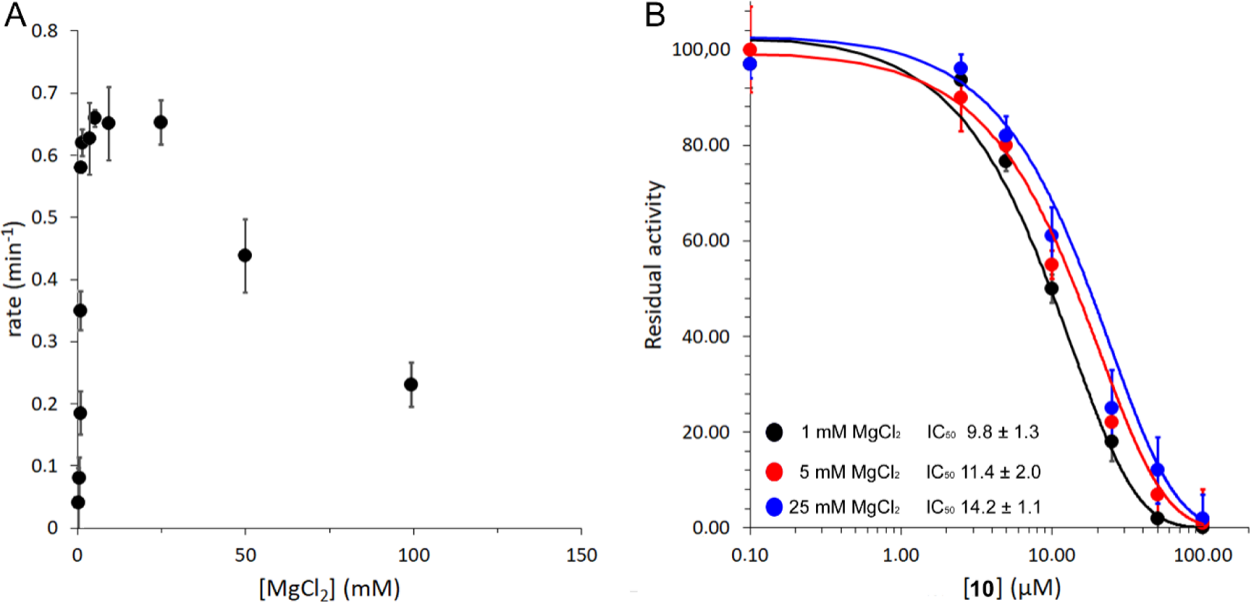
MbtI enzyme activity as a function of [Mg^2+^] (A). Profiles of the IC_50_ of **10**, obtained at different concentrations of Mg^2+^ (B).

Firstly, we acquired further biochemical data on **10**, testing its activity at different concentrations of Mg^2+^, with the aim of defining the role of the cofactor in the interaction between MbtI and the inhibitor. With this strategy, we envisaged that we would observe differences in the inhibitory effect of our ligand in the presence of varying amounts of Mg^2+^, if the ion were necessary for its binding and correct orientation in the active site. As expected, MbtI was found to be completely inactive in the absence of its cofactor, reaching the maximal activity only at 2 mM MgCl_2_. This result confirmed that the ion is essential for enzymatic activity, although the affinity seems rather moderate. Despite Ferrer et al.^17,18^ reported on the ability of MbtI to act as chorismate mutase in the absence of the metal, leading to the synthesis of prephenate, our data corroborate the hypothesis formulated by Ziebart and Toney^19^ that such activity is likely due to a faulty purification of the enzyme. Moreover, the use of increasing concentrations of Mg^2+^ (>50 mM) showed inhibitory effects, as the enzyme activity significantly decreased (Figure 5). Conversely, the enzyme exhibits no significant decrease in specific activity when assayed at concentrations of up to 500 mM KCl, confirming that inhibition is specific to Mg^2+^ and not due to increased ionic strength.

The determination of the activity of **10**, performed at different concentrations of Mg^2+^, showed that the metal did not strongly influence the binding of the compound to the target. However, in the presence of very high Mg^2+^ concentrations, the performance of **10** was slightly reduced. This observation further supported the hypothesis that at moderate concentrations of Mg^2+^, the ligand preferentially binds (and inhibits) MbtI, while the metal has a low affinity for the enzyme. At high Mg^2+^ concentrations, our data suggest that the ion nevertheless binds to the active site, occluding the catalytic region and blocking the access of the inhibitor. These data might also indicate that our compound could prevent the binding of Mg^2+^ in a competitive fashion, becoming slightly less effective when the concentration of the metal reaches a higher millimolar range (Figure 5).

### Synthesis and Activity of a Probe (**11**)

To date, most MbtI inhibitors reported in the literature, including our candidates, share a common carboxylate motif that is supposedly responsible for functional sequestration of the metal ion within the active site.^20^ However, according to our hypothesis, this interaction may not be necessary neither for the binding of the ligand nor for the resulting inhibitory effect. In order to further verify this theory, we designed and synthesized a probe molecule, introducing a chemical modification on **10**. The Mg^2+^ ion is characterized by a small ionic radius, high charge density, and a tendency to bind water molecules in the inner coordination sphere rather than bulkier ligands. Being a “hard” ion, it prefers to bind “hard” oxygen-containing ligands, such as carbonyls, carboxylates, phosphates, hydroxyls, and water.^21^ Therefore, the carboxylate pharmacophore motif was converted to an amide function, affording the analogue **11** (see Scheme S2 in Supporting Information). Its docking into MbtI in the absence of Mg^2+^ suggested that a very similar binding mode to that proposed for **10** was likely (Figure S62). The biological tests on compound **11** confirmed a very good inhibitory activity (IC_50_ = 17.3 ± 1.9 μM), thus supporting our hypothesis that metal chelation should not be considered an essential pharmacophore feature to develop inhibitors of MbtI.

### Structure of MbtI in Complex with **10**

In the context of our attempts to elucidate the structure of complexes between MbtI and 5-phenylfuran-2-carboxylic acid-based inhibitors, we obtained cocrystals with the lead compound **10**: structural data and refinement statistics are summarized in Table 2.

**Table 2:** Data Collection, Refinement, and Model Statistics of MbtI-**10**, MbtI–Mg^2+^, and MbtI–Ba^2+^^a^

	MbtI-**10**	MbtI–Mg^2+^	MbtI–Ba^2+^
space group	*P*2_1_	*I*422	*P*2_1_
Unit–Cell Parameters			
*a*, *b*, *c* (Å)	88.336, 111.687, 94.998	193.97, 193.97, 257.03	88.04, 116.90 94.09
α, β, γ (deg)	90, 92.67, 90	90, 90, 90	90, 91.60, 90
Diffraction Data			
resolution range (Å)	111.69–2.09 (2.13–2.09)	154.83–2.11 (2.15–2.11)	43.63–1.80 (1.85–1.80)
wavelength (Å)	0.97856	0.98012	0.98012
no. unique reflections	108510 (5396)	131041 (6551)	173404 (11664)
multiplicity	7.0 (7.1)	27.0 (26.5)	6.8 (6.5)
completeness (%)	99.9 (99.9)	93.9 (85.6)	99.2 (90.3)
average *I*/σ(*I*)	8.0 (2.1)	17.7 (1.4)	14.6 (1.0)
*R*_pim_^b^	0.063 (0.356)	0.028 (0.546)	0.030 (0595)
CC(1/2)	0.990 (0.803)	0.998 (0.534)	0.999 (0.622)
Refinement Statistics			
*R*_work_^c^	0.208	0.195	0.196
*R*_free_^c^	0.240	0.217	0.214
No. of Non-H Atoms^d^			
protein	12651	13151	13228
ligands	65	198	51
water	878	797	1351
Average *B*-Factors^d^			
protein	43.33	51.97	35.98
ligands	56.33	89.33	43.89
water	44.14	53.14	42.82
RMS Deviations^d^			
bonds (Å)	0.012	0.012	0.012
angles (deg)	1.45	1.50	1.45
molprobity clashscore^d^	3.13	2.90	1.51
Ramachandran outliers^d^ (%)	0.00	0.00	0.00
Ramachandran favoured^d^ (%)	98.98	98.34	98.80
rotamer outliers^d^ (%)	1.09	1.11	1.11
C-β outliers^d^ (%)	0.00	0.00	0.00
PDB code	6ZA4	6ZA5	6ZA6

a Data were indexed and scaled with XDSME (MbtI–Ba^2+^) or autoPROC (MbtI-**10** and MbtI–Mg^2+^), applying, in the latter case, an anisotropic resolution cut-off *via* STARANISO as implemented in autoPROC.^30^

b *R*_pim_ = Σ*h*[1/(/*n*_h_ – 1)]^1/2^Σ*i*|⟨*I*_h_⟩*I*_h,i_|/Σ*h*Σ*iI*_h,i_, where *I*_h_ is the intensity of the unique reflection *h*, whereas *I*_i_ is the intensity of each of its symmetry-equivalent reflections. CC(1/2) according to Karplus.^39^

c *R*_work_ = Σ||*F*_o_| – |*F*_c_||/Σ|*F*_o_|, where *F*_o_ and *F*_c_ are the observed and calculated structure factor amplitudes. Five percent of the reflections were reserved for the calculation of *R*_free_.

d Calculated with phenix.validate.^37^

These experiments were undertaken to definitively characterize the binding mode of our candidates, thus providing a reliable means to verify the results of the computational simulations and our previous observations. Furthermore, considering both the limited size of the compounds and the high plasticity of the active site, we were convinced that structural studies would assume a particular significance in the identification of the key enzyme–inhibitor interactions.^12^

Among the 3D structures of MbtI available in the PDB, two different conformations have been reported, which have been described as “open” (e.g., PDB ID 2G5F)^2^ and “closed” (e.g., PDB ID 3LOG),^7^ depending on the relative position of two mobile loops with respect to the active site. In the closed form, the flexible sequences (residues 268–293 and 324–336)^20^ are bent over the binding pocket, while in the open form, they are tilted upward. A comparative analysis of previously published structures allowed us to identify the intermolecular bonds responsible for this conformational shift. In particular, the closed state seems to be determined by the formation of a H-bond between a suitable moiety of the ligand and the NH of Gly270 and/or the hydroxyl group of Thr271: these contacts effectively drag the first mobile loop toward the active site. In turn, Thr271 establishes a bond with His334, thus pulling the second mobile loop in the same direction. The link between the flexible regions is further stabilized by additional interactions, formed by adjacent amino acids. Overall, this movement determines the capping of the active site. The chemical entity responsible for the interaction with Gly270 or Thr271 is a carbonate anion (or an ordered water molecule) in 3LOG and a carboxyl group in PDB IDs 3ST6 and 3VEH (chains A, B, C). Interestingly, in PDB IDs 3RV7, 3RV8, and 3RV9, the absence of this H-bond induces an open conformation, despite the orientation of the ligands does not differ significantly compared to the one observed in 3VEH. In chain D of 3VEH, the increased distance between the COOH and Thr271 (3.21 *vs* 2.70–2.89 Å) determines an intermediate conformation. Another intermediate, but overall open, state can be observed in 3RV7 chain B, in which a contact between Thr271 and Ser331 induces a slightly more closed configuration compared to the other chains. The peculiar binding mode of the bulkier inhibitor of PDB ID 3RV6 determines an unprecedented conformation, in which the opening of the mobile loops is characterized by a particularly wide angle. Notably, these mobile regions are characterized by high B-factors and partial lack of supporting electron density, especially when the conformation is open, despite some chains show stabilized conformations, mostly in the 324–336 loop, because of different crystal packing interactions involving these regions.

In the complex MbtI-**10**, the enzyme conformation can be defined as an open state for the ensemble of the four molecules in the asymmetric unit (ASU), despite the partially incomplete electron density of the mobile loops (unresolved regions for chain A: 271–276; chain B: 270–290 and 328–334; chain C: 275–282; chain D: 274–277). The adoption of this conformation is due to the fact that **10** does not possess a suitable functional group in the correct orientation to establish a H-bond with Gly270 or Thr271. Consequently, the arrangement of the flexible loops of MbtI-**10** is similar to that of 2G5F, 3RV7, 3RV8, and 3RV9 with root-mean-square deviations in the range 1–4 Å (calculated for residues 325–335).

**Figure 6:**
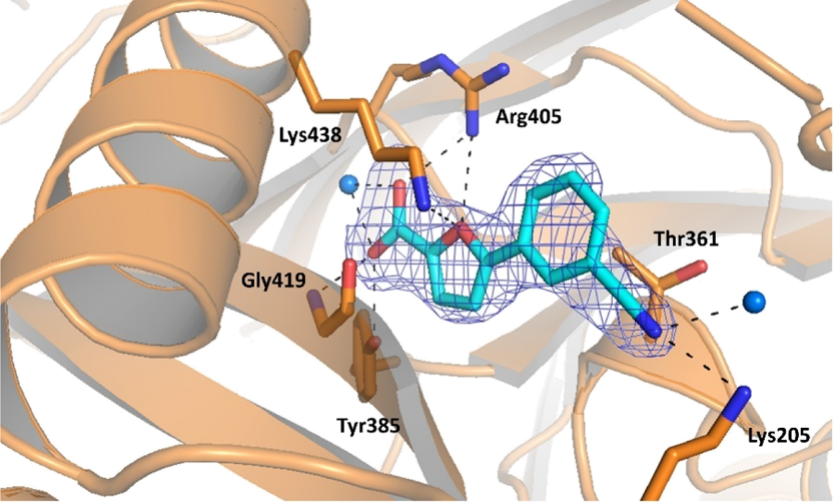
Ribbon diagram of the MbtI-**10** structure (PDB ID 6ZA4): the interactions of the ligand with the side chains (in sticks) and the water molecules (blue spheres) are represented as dashed lines. The blue mesh represents the electron density around the ligand (contoured at 1σ).

An electron density compatible with the presence of the ligand is detectable in all four chains of the ASU, with the furan-2-carboxylic acid region always well-defined and weaker electron density for the phenyl portion. The analysis of the binding mode of the compound revealed the presence of H-bonds between its carboxylic group and Tyr385, Arg405, Gly419, and an ordered water molecule; the oxygen of the furan interacts with Arg405, while the phenyl ring forms a cation−π interaction with Lys438 and a van der Waals contact with Thr361. Finally, the cyano group forms a H-bond with Lys205, a key amino acid involved in the first step of the catalytic reaction (Figure 6). This residue is likely responsible for the activation of a water molecule for nucleophilic attack (S_N_2″) on C2 of chorismate, which leads to the release of the hydroxyl group at position 4, after its protonation mediated by Glu252, thereby affording isochorismate.^22^ One may speculate that the absence of an interaction with the pivotal Lys205 in isochorismate-based derivatives accounts for their slightly weaker inhibitory activity with respect to **10**. The superposition of MbtI-**10** with 3RV7 (Figure S54) shows that the arrangement of the amino acid side chains around the ligands is similar, with the aromatic carboxylic group of the AMT derivative occupying the same position as the acid moiety of **10**. The most significant difference is represented by the ability of the nitrile group of **10** to form a H-bond with Lys205, thereby hindering its physiological functions.

Despite our experiments were carried out in the presence or in the absence of Mg^2+^ in the crystallization buffer (2–5 mM of different Mg^2+^ salts), we never observed any bound Mg^2+^ in the MbtI-**10** cocrystal structure. The orientation of the ligand is substantially in agreement with the predicted binding mode of **10** in the absence of Mg^2+^, as shown by the overlay of the two models (Figure S55). The binding poses in the two complexes exhibit a root-mean-square deviation of 1.6 Å and similar interactions. The main differences are related to the orientation of the side chain of Arg405, which was not predicted to interact with the ligand and the side chains of Thr271 and His334, which are shifted away from the binding site. As previously mentioned, the absence of interactions between the ligand with the amide group of Gly270 or the hydroxyl group of Thr271 seems to favor the outward movement of the backbone in the two mobile loops, thereby leading to an open conformation. Such shift could hardly be predicted by computational simulations, as those were based on a closed form of the enzyme. As for Arg405, the analysis of electron density indicates a high flexibility of its side chain, which is otherwise modeled, where supporting density is present as different rotamers.

### Structure of MbtI with the Mg^2+^ Ion

Although the role of the Mg^2+^ cofactor in the catalytic activity of MbtI has been the subject of a number of publications,^4^ its role remains elusive. Our numerous attempts to obtain a structure of MbtI in ternary complex with **10** and Mg^2+^ failed, invariably leading to the cocrystal described above. Notably, the only available structure of MbtI showing a bound Mg^2+^ ion was obtained by Chi and co-workers (3RV6).^20^ However, as stated by the authors, its binding mode under those conditions may not necessarily reproduce the physiological situation: the ion was found to be hexacoordinated by water molecules, interacting with the enzyme and the inhibitor only through the surrounding waters, inconsistently with both its supposed role in catalysis^18^ and the authors’ predicted binding mode. Therefore, we decided to further pursue the investigation of the binding and coordination of Mg^2+^ into the active site of MbtI in order to clarify its role in the enzymatic mechanism.

Considering the inherent difficulty in obtaining MbtI–Mg^2+^ cocrystals and the results of our biochemical assays, we hypothesized that we could compensate for the rather low affinity of MbtI for its cofactor by using saturating concentrations of Mg^2+^. Interestingly, 2.1 Å resolution data were collected from a non-isomorphous crystal grown under these conditions (Table 2).

**Figure 7:**
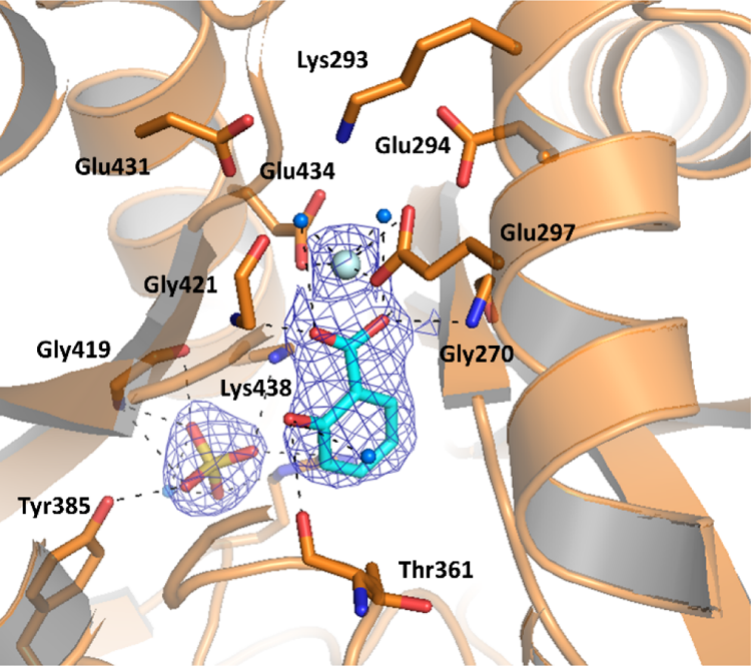
Ribbon diagram of the MbtI–Mg^2+^ structure (PDB ID 6ZA5) focused on the active site of chain D. The interactions of the ligands with the side chains (in sticks), the Mg^2+^ ion (light blue sphere), and the water molecules (blue spheres) are represented as dashed lines. The blue mesh represents the electron density around the ligand (contoured at 1σ).

**Figure 8:**
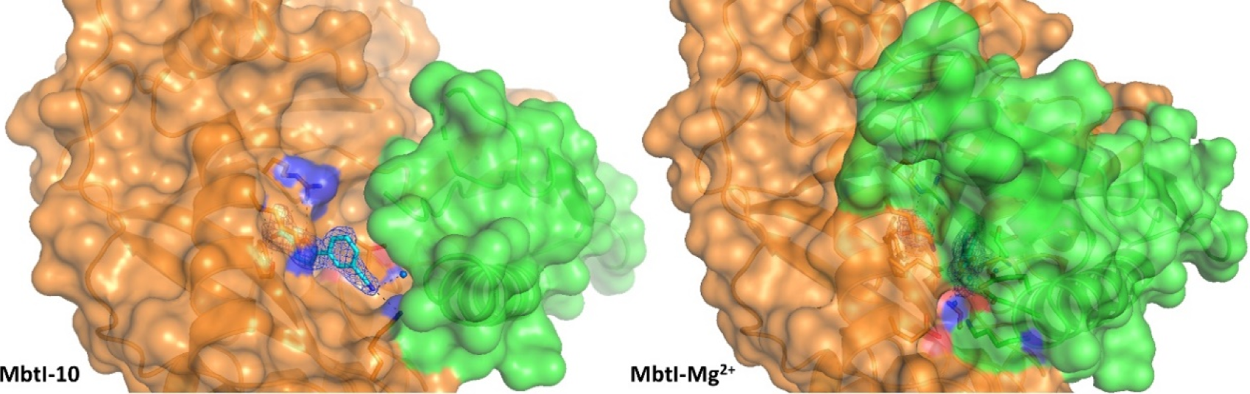
Comparison of surfaces of MbtI-**10** (left, PDB ID 6ZA4) and MbtI–Mg^2+^(right, PDB ID 6ZA5), showing the movement of the mobile loops (in green, only partially traced in MbtI-**10** because of the lack of supporting electron density). The active site accessibility determines the conformational state of the enzyme (open, in MbtI-**10**, *vs* closed in MbtI–Mg^2+^). The blue mesh represents the electron density around active site ligands (contoured at 1σ); the side chains of the interacting amino acids are in sticks.

The crystal structure derived from these data presents four molecules in the ASU, with a well-defined electron density in all regions, including the previously observed mobile loops that are now stabilized into a closed conformation, superimposable with the one observed in 3LOG or 3ST6.^7,20^ The analysis of the active site evidenced electron density fully consistent with the presence of a Mg^2+^ ion, though its coordination sphere is slightly different in the various chains. In chain A (Figure S56), the Mg^2+^ interacts with the oxygen of Gly421, with four ordered water molecules, and with a sulfate anion. Through two of the water molecules, it also contacts Glu431 and Glu434. Chain C does not show the presence of the ion; in its absence, Glu431 binds a water molecule and there is no clear electron density for the side chain of Glu434. The coordination pattern of Mg^2+^ in chains B and D is analogous (Figure 7): the ion could be modeled as directly interacting with Glu297, Glu434, and two ordered water molecules, which in turn make H-bonds to Glu294 and Glu431. In addition to that, the metal forms a strong interaction with another molecule, which, by analogy with the crystal structure of Irp9 from *Yersinia enterocolitica* (PDB ID 2FN1), could be identified and modeled as a salicylate.^23^ Supporting density for the bound catalytic product could be identified in two protein chains over four, where the pose of the refined ligand was perfectly superimposable to that of 2FN1 (Figure S57). The salicylate coordinates the Mg^2+^ ion with its carboxylic moiety, which also forms additional H-bonds with the peptide backbone through Gly270 and Gly421 and with the side chain of Thr271. The hydroxyl group and the phenyl ring do not seem to form significant interactions. As previously noted, the bond between the carboxylic function and Gly270–Thr271 is responsible for the adoption of the closed conformation (Figure 8). The comparison of this structure with MbtI-**10** and 3RV7 showed a moderate rearrangement of the residues in the active site (Figure S58). Both the side chains of the interacting amino acids and some portions of the main chain backbone (Gly270) are tightened around the ion and salicylate, narrowing the binding pocket. The orientation of the salicylate is almost orthogonal to the plane formed by the rings of the inhibitors. Therefore, the aromatic carboxylic groups of the ligands and the carboxylate of the natural product do not occupy the same position: instead, the acidic moiety of the inhibitors is here replaced by a sulfate anion. Conversely, the aliphatic carboxylic function in 3RV7 may be oriented toward the ion by rotation around the ether bond. The superposition of our Mg^2+^-bound structure with the closed structures of 3VEH and 3ST6 showed a much similar arrangement of the residues in the binding pocket, with the exception of some amino acids involved in the binding of Mg^2+^, which are tilted away in the literature structures (Figure S59). Moreover, while the orientation of the compound in 3VEH is comparable to that of 3RV7, the position of the aromatic analogue of isochorismate in 3ST6 is overturned, with the aromatic portion occupying roughly the same position as the salicylate. This is the only AMT derivative which exhibits such an orientation. As mentioned above, the side chain of Arg405 also shows deviations because of its flexibility.

The presence of bound salicylate in the structure is remarkable, especially considering that the natural substrate was not added to the crystallization solution. To confirm the presence of a salicylate molecule bound to the protein, MbtI (25 mg/mL) was heat-denatured to release any bound compound and centrifuged; the resulting supernatant was subjected to UPLH-MS analysis. As shown in Figure S63, a peak corresponding to that of the salicylic acid was detected in the supernatant of the treated MbtI and not in controls. This finding suggests that the enzyme is active in the heterologous expression host (*Escherichia coli*) and that either chorismate or salicylate, both of which are known to be intermediates in *E. coli* aromatic biosynthetic pathways, is copurified with the protein. It is worth to note that the first described crystal structure of MbtI (2G5F), also reportedly copurified from *E. coli*, showed the presence of pyruvate in the active site, which thus prompted the authors to classify the enzyme as a salicylate synthase.^2^ Unsurprisingly, the position that was occupied by the pyruvate is here taken by a sulfate ion, provided in the crystallization solution as the Mg^2+^ counterion. At increasing MgCl_2_ concentrations, the enzyme catalytic turnover is significantly reduced, strongly supporting the notion that high Mg^2+^ concentrations stabilize the closed conformation of the active site, preventing the loss of salicylate from the active site. Besides, the direct salicylate–Mg^2+^ interaction, shown here for the first time for MbtI, is consistent with biochemical observations on the role of Mg^2+^ by Meneely et al.^6^

Furthermore, the indirect confirmation of the binding position of Mg^2+^ in the active site of MbtI was obtained from another structure, solved from crystals grown in the presence of chorismate in its commercially available Ba^2+^ salt (Table 2). Despite no evidence of bound chorismate was found from the analysis of the electron density maps, a bound Ba^2+^ ion was identified (Figure S60). This divalent ion roughly occupies the same position as Mg^2+^ (Figure S61), further confirming the presence of a metal binding site. Ba^2+^ forms a similar pattern of interactions with the surrounding residues, with slight modifications because of its different steric hindrance. Moreover, as in the Mg^2+^-bound structure, the enzyme exhibits the same closed conformation: in this case, the H-bond with Thr271 is established by an oxygen of a phosphate anion, originating from the crystallization condition.

## Conclusions

In this work, we report the discovery of a new pharmacophore model based on the crystal structure of *M. tuberculosis* salicylate synthase (MbtI) complexed with **10**, the most potent inhibitor discovered to date, also active against isolated mycobacterial cells. Our experiments proved the connection between its antimycobacterial effect and the disruption of mycobactin biosynthesis, confirming the importance of this pathway as a target for novel anti-TB therapies. The MbtI-**10** structure highlighted a new mode of ligand–protein interaction in which the binding of the ion is not a key feature for the development of improved inhibitors. Moreover, the cocrystal structure highlighted the importance of fundamental residues for the binding of the ligand, such as Thr361, Tyr385, Arg405, and Lys438, and in particular of Lys205, a key amino acid involved in the first step of the catalytic reaction. These findings constitute the basis for the development of a novel pharmacophore model for MbtI ligands, which could be profitably employed in virtual screening protocols aimed at the identification of structurally novel inhibitors and lead optimization campaigns focused on the development of more potent analogues of compound **10**.

In addition, the X-ray structure of MbtI complexed with Mg^2+^ is consistent with the proposed mechanism of the enzyme and, most notably, the role of the Mg^2+^ cofactor. The crystal structure of the ternary complex of MbtI with Mg^2+^ and salicylate shows the product of the catalytic reaction, chelating the metal ion with its carboxylic moiety and forming additional H-bonds with Gly270, Gly421, and Thr271. Observing such a conformation in this ternary complex is not surprising when high concentrations of Mg^2+^ are present; consistent with previous hypothesis and biochemical data, the bound Mg^2+^ ion stabilizes the enzyme in a close conformation, which in turn prevents the loss of salicylate from the active site.

These findings contribute to fill the gap in the understanding of the kinetic and chemical mechanisms of this enzyme, supporting the development of high affinity inhibitors. Given the structural similarity between MbtI and other MST enzymes, designing new antimicrobials endowed with a multi-target activity is now a foreseeable scenario.

## Experimental Section

### Chemistry

All starting materials, chemicals, and solvents were purchased from commercial suppliers (Sigma-Aldrich, St. Louis, MI, USA; FluoroChem, Hadfield, UK; Carlo Erba, Cornaredo, Italy) and used as received. Anhydrous solvents were utilized without further drying. Aluminum-backed silica gel 60 plates (0.2 mm; Merck, Darmstadt, Germany) were used for analytical thin-layer chromatography (TLC) to follow the course of the reactions. Microwave-assisted reactions were carried out with a Biotage Initiator Classic (Biotage, Uppsala, Sweden). Silica gel 60 (40–63 μm; Merck) was used for the purification of intermediates and final compounds through flash column chromatography. Melting points were determined in open capillary tubes with a Stuart SMP30 melting point apparatus (Cole-Parmer Stuart, Stone, UK). All tested compounds were characterized by means of ^1^H NMR, ^13^C NMR, FT-IR, and HRMS. ^1^H and ^13^C NMR spectra were acquired at ambient temperature with a Varian Oxford 300 MHz instrument (Varian, Palo Alto, CA, USA), operating at 300 MHz for ^1^H and 75 MHz for ^13^C. Chemical shifts are expressed in ppm (δ) from tetramethylsilane resonance in the indicated solvent (TMS: δ = 0.0 ppm), while *J*-couplings are given in Hertz. The APT sequence was used when deemed necessary. The 2D-NOESY sequence was employed to unambiguously assign the hydrogen signals, when appropriate; this experiment was performed on a Bruker Avance 300 MHz instrument (Bruker, Billerica, MA, USA). IR spectra were acquired with a PerkinElmer Spectrum One FT-IR (PerkinElmer, Waltham, MA, USA), in a spectral region between 4000 and 450 cm^–1^, and analyzed by a transmittance technique with 32 scansions and 4 cm^–1^ resolution. Solid samples were mixed in a mortar with KBr (1:100) and pressed to small tablets using a hydraulic press (14 tons). The purity of the final compounds was assessed by means of LC-high-resolution mass spectrometry (Q Exactive Hybrid Quadrupole-Orbitrap mass spectrometer; Thermo Fischer, Waltham, MA, USA) and was ≥95%.

Compound **1** was purchased from Sigma-Aldrich at the highest purity level available (≥95%) and tested as received.

### General Procedure A

Procedure A1. The appropriate methyl ester derivative (1 mmol) was dissolved in a mixture of tetrahydrofuran (THF)/EtOH 1:1 (15 mL), and a 1 M solution of NaOH (2.5 mmol) was added dropwise while stirring. The reaction mixture was heated at reflux for 5 h. After completion, the solvent was evaporated under reduced pressure; the aqueous phase was washed with CHCl_3_ (1 × 5 mL), acidified with 3 M HCl, and then extracted with EtOAc (3 × 7 mL). The organic layers were washed with brine, dried over anhydrous Na_2_SO_4_, and then concentrated *in vacuo*. The resulting solid was washed with cool hexane (3 mL). Procedure A2. The appropriate methyl ester derivative (1 mmol) was treated with LiOH·H_2_O (3.0 mmol) in a mixture of THF–H_2_O 2:1 (15 mL) at room temperature for 2 h. After completion, the solution was acidified with 1 M HCl and then extracted with EtOAc (3 × 7 mL). The organic layers were washed with brine, dried over anhydrous Na_2_SO_4_, and then concentrated *in vacuo*.^11,12^

### General Procedure B

The suitable aromatic ester (1 mmol), the appropriate phenylboronic acid (1.3 mmol), and bis(triphenylphosphine)palladium(II) dichloride (5% mol) were dissolved in dry 1,4-dioxane (10 mL) under a N_2_ atmosphere Na_2_CO_3_ solution (2 M, 2 mmol) was added, and the resulting mixture was stirred overnight at 90 °C. After completion, the solution was cooled to room temperature and then filtered on a celite pad. The filtrate was diluted with H_2_O and extracted with EtOAc (3 × 4 mL). The organic layer was dried over anhydrous Na_2_SO_4_, filtered and concentrated *in vacuo*.^11,12^

### General Procedure C

Methyl 5-boronofuran-2-carboxylate (**17**, 1.3 mmol), the appropriate bromo-derivatives (1.0 mmol), and bis(triphenylphosphine)palladium(II) dichloride (5% mol) were dissolved in dry 1,4-dioxane (10 mL) under a N_2_ atmosphere. Na_2_CO_3_ solution (2 M, 2 mmol) was then added, and the resulting mixture was stirred in a microwave synthesizer (Biotage Initiator Classic) for 1 h at 60 °C. After completion, the solution was cooled to room temperature and filtered on a celite pad. The filtrate was diluted with H_2_O and extracted with EtOAc (3 × 4 mL). The organic layer was dried over anhydrous Na_2_SO_4_, filtered, and concentrated *in vacuo*.^11,12^

### General Procedure D

The appropriate aromatic carboxylic acid (1 mmol) was dissolved in MeOH (4.8 mL) before concentrated H_2_SO_4_ (0.7 mL) was added dropwise while stirring. The reaction mixture was refluxed for 24 h. After completion, the solution was cooled to room temperature and MeOH was removed *in vacuo*; the aqueous phase was treated with a saturated solution of NaHCO_3_ to ensure neutral-basic pH and then extracted with EtOAc (3 × 4 mL). The combined organic layers were washed with brine, dried over anhydrous Na_2_SO_4_, filtered, and concentrated *in vacuo*.^24^

#### 5-(3-Chlorophenyl)furan-2-carboxylic Acid (**2**)

Procedure A1. Starting compound: methyl 5-(3-chlorophenyl)furan-2-carboxylate (50 mg, 0.21 mmol, **12**). Light brown solid. Yield: 42 mg, 0.19 mmol, 89%. mp 187 °C (dec.). TLC (dichloromethane (DCM)–MeOH 8:2): *R*_f_ = 0.27. ^1^H NMR (300 MHz, DMSO-*d*_6_): δ (ppm) 7.76 (t, *J* = 1.7 Hz, 1H, H_7_), 7.67 (d, *J* = 7.7, 1H, H_11_), 7.44 (t, *J* = 7.7 Hz, 1H, H_10_), 7.33 (d, *J* = 7.7, 1H, H_9_), 7.06 (d, *J* = 3.4 Hz, 1H, H_3_), 6.88 (s, 1H, H_4_). ^13^C NMR (75 MHz, DMSO-*d*_6_): δ 160.72, 160.45, 160.36, 151.98, 134.45, 132.97, 131.56, 128.09, 123.92, 122.95, 109.29. FT-IR (KBr) ν cm^–1^: 3412, 2961, 2920, 2851, 1674, 1660, 1602, 1525, 1461, 1420, 1384, 1305, 1278, 776. HRMS (ESI-QOrbitrap) *m*/*z*: calcd for C_11_H_7_ClO_3_ [M – H]^−^, 221.0011; found, 221.0022.

#### 5-(3-Hydroxyphenyl)furan-2-carboxylic Acid (**3**)

Procedure A1. Starting compound: methyl 5-(3-hydroxyphenyl)furan-2-carboxylate (55 mg, 0.25 mmol, **13**). White solid. Yield: 48 mg, 0.24 mmol, 94%. mp 170 °C. TLC (DCM–MeOH 7:3): *R*_f_ = 0.40. ^1^H NMR (300 MHz, DMSO-*d*_6_): δ (ppm) 13.10–13.00 (bs exch. D_2_O, 1H, COOH), 9.80–9.60 (bs exch. D_2_O, 1H, OH), 7.26 (d, *J* = 3.4 Hz, 1H, H_3_), 7.18–7.06 (m, 3H, H_7,10,11_), 7.04 (d, *J* = 3.4 Hz, 1H, H_4_), 6.77 (dt, *J* = 7.3, 2.1 Hz, 1H, H_9_). ^13^C NMR (75 MHz, DMSO-*d*_6_): δ (ppm) 159.72, 158.30, 156.76, 144.46, 130.77, 130.75, 122.18, 116.55, 115.83, 111.33, 108.19. FT-IR (KBr) ν cm^–1^: 3401, 2919, 1675, 1649, 1582, 1533, 1470, 1418, 1309, 1298, 1284, 1200, 1160, 1025, 954, 852, 799, 785. HRMS (ESI-QOrbitrap) *m*/*z*: calcd for C_11_H_9_O_4_ [M + H]^+^, 205.0495; found, 205.0499.

#### 5-(*m*-Tolyl)furan-2-carboxylic Acid (**4**)

Procedure A1. Starting compound: methyl 5-(*m*-tolyl)furan-2-carboxylate (48 mg, 0.22 mmol, **18**). Brown solid. Yield: 39 mg, 0.19 mmol, 86%. mp 147 °C. TLC (DCM–MeOH 8:2): *R*_f_ = 0.35. ^1^H NMR (300 MHz, DMSO-*d*_6_): δ (ppm) 13.07 (bs exch. D_2_O, 1H, COOH), 7.62–7.56 (m, 2H, H_7,11_), 7.34 (t, *J* = 7.7 Hz, 1H, H_10_), 7.29 (d, *J* = 3.7 Hz, 1H, H_3_), 7.19 (d, *J* = 7.7 Hz, 1H, H_9_), 7.09 (d, *J* = 3.7 Hz, 1H, H_4_), 2.35 (s, 3H, CH_3_). ^13^C NMR (75 MHz, DMSO-*d*_6_): δ (ppm) 159.71, 156.85, 144.50, 138.82, 130.08, 129.59, 129.38, 125.23, 122.09, 120.29, 108.24, 21.35. FT-IR (KBr) ν cm^–1^: 3435, 2918, 2666, 2611, 2573, 1674, 1609, 1593, 1573, 1518, 1472, 1421, 1366, 1312, 1275, 1218, 1164, 1024, 788, 760. HRMS (ESI-QOrbitrap) *m*/*z*: calcd for C_12_H_10_O_3_ [M + H]^+^, 203.0703; found, 203.0702.

#### 5-(3-Aminophenyl)furan-2-carboxylic Acid (**5**)

Procedure A1. Starting compound: methyl 5-(3-aminophenyl)furan-2-carboxylate (40 mg, 0.18 mmol, **22**). Yellow solid. Yield: 33 mg, 0.16 mmol, 89%. mp 225 °C (dec.). TLC (DCM–MeOH 7:3): *R*_f_ = 0.33. ^1^H NMR (300 MHz, DMSO-*d*_6_): δ (ppm) 7.25 (d, *J* = 3.7 Hz, 1H, H_3_), 7.10 (t, *J* = 7.9 Hz, 1H, H_10_), 6.99 (t, *J* = 2.0 Hz, 1H, H_7_), 6.92 (m, 1H, H_11_), 6.91 (d, *J* = 3.7 Hz, 1H, H_4_), 6.56 (ddd, *J* = 7.9, 2.0, 1.0 Hz, 1H, H_9_). ^13^C NMR (75 MHz, DMSO-*d*_6_): δ (ppm) 159.79, 157.61, 149.65, 144.21, 130.12, 129.95, 120.11, 115.09, 112.75, 109.69, 107.49. FT-IR (KBr) ν cm^–1^: 3439, 3350, 3241, 3122, 3056, 2961, 2920, 2850, 1682, 1621, 1604, 1573, 1525, 1490, 1471, 1376, 1257, 959, 949, 803, 781. HRMS (ESI-QOrbitrap) *m*/*z*: calcd C_11_H_9_NO_3_ [M – H]^−^, 202.0510; found, 202.0512.

#### 5-(3-Carbamoylphenyl)furan-2-carboxylic Acid (**6**)

Procedure A1. Starting compound: methyl 5-(3-carbamoylphenyl)furan-2-carboxylate (45 mg, 0.18 mmol, **15**). White solid. Yield: 34 mg, 0.15 mmol, 81%. mp 250 °C. TLC (DCM–MeOH 7:3): *R*_f_ = 0.29. ^1^H NMR (300 MHz, DMSO-*d*_6_): δ (ppm) 13.10–13.00 (bs exch. D_2_O, 1H, COOH), 8.27 (s, 1H, H_7_), 8.11 (bs exch. D_2_O, 1H, NH_2_), 7.92 (d, *J* = 7.8 Hz, 1H, H_9_), 7.86 (d, *J* = 7.8 Hz, 1H, H_11_), 7.55 (t, *J* = 7.8 Hz, 1H, H_10_), 7.49 (bs exch. D_2_O, 1H, NH_2_), 7.33 (d, *J* = 3.6 Hz, 1H, H_3_), 7.19 (d, *J* = 3.6 Hz, 1H, H_4_). ^13^C NMR (75 MHz, DMSO-*d*_6_): δ (ppm) 167.68, 159.69, 156.09, 144.93, 135.59, 129.69, 129.56, 128.19, 127.39, 123.90, 120.26, 108.92. FT-IR (KBr) ν cm^–1^: 3451, 3188, 2920, 2649, 2527, 1682, 1598, 1518, 1449, 1403, 1299, 1273, 1160, 1025, 958, 942, 795, 761. HRMS (ESI-QOrbitrap) *m*/*z*: calcd for C_12_H_10_NO_4_ [M + H]^+^, 232.0604; found, 232.0614.

#### 5-(3-(Methylcarbamoyl)phenyl)furan-2-carboxylic Acid (**7**)

Procedure A2. Starting compound: methyl 5-(3-(methylcarbamoyl)phenyl)furan-2-carboxylate (41 mg, 0.17 mmol, **19**). Yellow solid. Yield: 87%. mp 159 °C. TLC (DCM–MeOH 7:3): *R*_f_ = 0.32. ^1^H NMR (300 MHz, DMSO-*d*_6_): δ (ppm) 8.58 (q exch. D_2_O, *J* = 4.5 Hz, 1H, NH), 8.22 (t, *J* = 1.7 Hz, 1H, H_7_), 7.92 (dt, *J* = 7.8, 1.7 Hz, 1H, H_9_), 7.82 (dt, *J* = 7.8, 1.7 Hz, 1H, H_11_), 7.55 (t, *J* = 7.8 Hz, 1H, H_10_), 7.33 (d, *J* = 3.6 Hz, 1H, H_3_), 7.18 (d, *J* = 3.6 Hz, 1H, H_4_), 2.80 (d, *J* = 4.5 Hz, 3H, CH_3_). ^13^C NMR (75 MHz, DMSO-*d*_6_): δ (ppm) 166.54, 159.67, 156.10, 144.87, 135.85, 129.70, 129.62, 127.83, 127.28, 123.42, 120.31, 108.95, 26.73. FT-IR (KBr) ν cm^–1^: 3324, 3116, 3066, 2925, 1718, 1692, 1649, 1584, 1549, 1521, 1481, 1468, 1423, 1407, 1310, 1260, 1220, 1158, 1026, 803, 760. HRMS (ESI-QOrbitrap) *m*/*z*: calcd for C_13_H_11_NO_4_ [M + H]^+^, 246.0761; found, 246.0761.

#### 5-(3-Sulfamoylphenyl)furan-2-carboxylic Acid (**8**)

Procedure A1. Starting compound: methyl 5-(3-sulfamoylphenyl)furan-2-carboxylate (50 mg, 0.18 mmol, **20**). Yellow solid. Yield: 40 mg, 0.15 mmol, 84%. mp 261 °C (dec.). TLC (DCM–MeOH 7:3): *R*_f_ = 0.24. ^1^H NMR (300 MHz, DMSO-*d*_6_): δ (ppm) 13.22 (bs exch. D_2_O, 1H, COOH), 8.21 (t, *J* = 1.8 Hz, 1H, H_7_), 8.03 (dt, *J* = 7.9, 1.8 Hz, 1H, H_11_), 7.81 (dt, *J* = 7.9, 1.8 Hz, 1H, H_9_), 7.67 (t, *J* = 7.9 Hz, 1H, H_10_), 7.48 (s exch. D_2_O, 2H, NH_2_), 7.35 (d, *J* = 3.7 Hz, 1H, H_3_), 7.26 (d, *J* = 3.7 Hz, 1H, H_4_). ^13^C NMR (75 MHz, DMSO-*d*_6_): δ (ppm) 170.43, 159.61, 155.14, 145.55, 145.25, 130.28, 130.23, 128.10, 126.17, 121.31, 120.28, 109.67. FT-IR (KBr) ν cm^–1^: 3340, 3252, 2919, 2844, 2671, 2573, 1669, 1692, 1518, 1458, 1424, 1341, 1322, 1266, 1220, 1161, 1028, 890, 793, 762. HRMS (ESI-QOrbitrap) *m*/*z*: calcd for C_12_H_8_O_5_ [M – H]^−^, 266.0129; found, 266.0129.

#### 5-(3-Carboxyphenyl)furan-2-carboxylic Acid (**9**)

Procedure A1. Starting compound: methyl 5-(3-(methoxycarbonyl)phenyl)furan-2-carboxylate (60 mg, 0.23 mmol, **21**). White solid. Yield: 50 mg, 0.20 mmol, 88%. mp > 300 °C. TLC (DCM–MeOH 8:2): *R*_f_ = 0.26. ^1^H NMR (300 MHz, DMSO-*d*_6_): δ (ppm) 13.20 (2H, bs exch. D_2_O, 2H, COOH), 8.30 (s, 1H, H_7_), 8.04 (d, *J* = 7.8, 1H, H_9_), 7.92 (d, *J* = 7.8, 1H, H_11_), 7.60 (t, *J* = 7.8 Hz, 1H, H_10_), 7.32 (d, *J* = 3.6 Hz, 1H, H_3_), 7.25 (d, *J* = 3.6 Hz, 1H, H_4_). ^13^C NMR (75 MHz, DMSO-*d*_6_): δ (ppm) 167.2, 159.7, 155.6, 145.0, 132.2, 129.9, 129.8, 129.1, 125.2, 120.2, 119.2. FT-IR (KBr) ν cm^–1^: 3435, 2966, 2917, 2851, 2661, 2546, 1681, 1612, 1521, 1455, 1420, 1298, 1165, 1030, 804 761. HRMS (ESI-QOrbitrap) *m*/*z*: calcd for C_12_H_8_O_5_ [M – H]^−^, 231.0299; found, 231.0301.

#### 5-(3-Cyanophenyl)furan-2-carboxylic Acid (**10**)

Procedure A2. Starting compound: methyl 5-(3-cyanophenyl)furan-2-carboxylate (46 mg, 0.20 mmol, **16**). White solid. Yield: 38 mg, 0.18 mmol, 87%. mp 260 °C (dec.). TLC (DCM–MeOH 8:2): *R*_f_ = 0.20. ^1^H NMR (300 MHz, DMSO-*d*_6_): δ (ppm) 13.30 (bs exch D_2_O, 1H, COOH), 8.28 (s, 1H, H_7_), 8.10 (d, *J* = 7.8 Hz, 1H, H_9_); 7.80 (d, *J* = 7.8 Hz, 1H, H_11_), 7.69 (t, *J* = 7.8 Hz, 1H, H_10_), 7.34 (s, 2H, H_3,4_). ^13^C NMR (75 MHz, DMSO-*d*_6_): δ (ppm) 159.61, 154.33, 145.60, 132.55, 130.87, 130.75, 129.00, 128.36, 120.12, 118.77, 112.81, 110.22. FT-IR (KBr) ν cm^–1^: 3112, 2915, 2850, 2666, 2576, 2231, 1710, 1686, 1608, 1572, 1519, 1475, 1436, 1318, 1289, 1232, 1174, 1033, 997, 819, 801, 761, 582. HRMS (ESI-QOrbitrap) *m*/*z*: calcd for C_12_H_7_NO_3_ [M – H]^−^, 212.0353; found, 212.0351.

#### 5-(3-Cyanophenyl)furan-2-carboxamide (**11**)

1-[Bis(dimethylamino)methylene]-1*H*-1,2,3-triazolo[4,5-*b*]pyridinium 3-oxide hexafluorophosphate (HATU) (178 mg, 0.47 mmol) and *N*,*N*-diisopropylethylamine (0.32 mL, 1.9 mmol) were added to a solution of 5-(3-cyanophenyl)furan-2-carboxylic acid (100 mg, 0.47 mmol, **10**) in *N*,*N*-dimethylformamide (DMF) (2.50 mL), and the resulting mixture was stirred for 30 min at room temperature. Then, NH_4_Cl (75 mg, 1.4 mmol) was added, and the stirring was continued for 2 more hours. After completion, the reaction was neutralized with 1 M HCl and partitioned between EtOAc and H_2_O. The organic layer was washed three times with cold H_2_O, dried over anhydrous Na_2_SO_4_, and concentrated *in vacuo*.^25^ The crude product was purified by crystallization from DCM/hexane to afford an off-white solid. Yield: 32 mg, 0.15 mmol, 32%. mp 202 °C. TLC (DCM–MeOH 95:5): *R*_f_ = 0.27. ^1^H NMR (300 MHz, DMSO-*d*_6_): δ (ppm) 8.45 (t, *J* = 1.7 Hz, 1H, H_7_), 8.21 (d, *J* = 7.8 Hz, 1H, H_11_), 8.08 (bs exch. D_2_O, 1H, N*H*), 7.80 (d, *J* = 7.8, 1H, H_9_), 7.65 (t, *J* = 7.8 Hz, 1H, H_10_), 7.52 (bs exch. D_2_O, 1H, N*H*), 7.25 (d, *J* = 3.6 Hz, 1H, H_3_), 7.15 (d, *J* = 3.6 Hz, 1H, H_4_). ^13^C NMR (75 MHz, DMSO-*d*_6_): δ (ppm) 159.51, 152.57, 148.48, 132.29, 131.06, 130.62, 129.01, 128.15, 118.96, 116.18, 112.66, 109.92. FT-IR (KBr) ν cm^–1^: 3474, 3167, 2962, 2923, 2852, 2225, 1697, 1614, 1535, 1518, 1470, 1425, 1395, 1261, 1099, 1037, 958, 903, 893, 798. HRMS (ESI-QOrbitrap) *m*/*z*: calcd for C_12_H_8_N_2_O_2_ [M + H]^+^, 213.0659; found, 213.0659.

#### Methyl 5-Bromofuran-2-carboxylate (**12**)

Procedure D. Starting compound 5-bromo-2-furoic acid (500 mg, 2.6 mmol). White solid. Yield: 480 mg, 2.3 mmol, 89%. mp 64 °C. TLC (cyclohexane–EtOAc 8:2): *R*_f_ = 0.59. ^1^H NMR (300 MHz, CDCl_3_): δ (ppm) 7.12 (d, *J* = 3.5 Hz, 1H, H_3_), 6.45 (d, *J* = 3.5 Hz, 1H, H_4_), 3.89 (s, 3H, CH_3_).

#### Methyl 5-(3-Chlorophenyl)furan-2-carboxylate (**13**)

Procedure B. Starting compounds: methyl 5-bromofuran-2-carboxylate (400 mg, 2.0 mmol, **12**) and (3-chlorophenyl)boronic acid (407 mg, 2.6 mmol). The crude was purified by flash column chromatography (cyclohexane–EtOAc 8:2) to give the desired product as a white solid. Yield: 321 mg, 1.4 mmol, 68%. mp 76 °C. TLC (cyclohexane– EtOAc 8:2): *R*_f_ = 0.53. ^1^H NMR (300 MHz, CDCl_3_): δ (ppm) 7.76 (s, 1H, H_7_), 7.64 (d, *J* = 7.3 Hz, 1H, H_11_), 7.40–7.13 (m, 3H, H_3,9,10_), 6.75 (d, *J* = 3.6 Hz, 1H, H_4_), 3.92 (s, 3H, CH_3_).

#### Methyl 5-(3-Hydroxyphenyl)furan-2-carboxylate (**14**)

Procedure B. Starting compounds: methyl 5-bromofuran-2-carboxylate (400 mg, 2.0 mmol, **12**) and (3-hydroxyphenyl)boronic acid (359 mg, 2.6 mmol). The crude was purified by flash column chromatography (cyclohexane–EtOAc 8:2) to give the desired product as a white solid. Yield: 135 mg, 0.62 mmol, 31%. mp 149 °C. TLC (cyclohexane–EtOAc 8:2): *R*_f_ = 0.23. ^1^H NMR (300 MHz, CDCl_3_): δ (ppm) 7.51–7.10 (m, 4H, H_3,7,10,11_), 6.85 (d, *J* = 7.6 Hz, 1H, H_9_), 6.72 (d, *J* = 3.6 Hz, 1H, H_4_), 5.34 (bs exch. D_2_O, 1H, OH), 3.92 (s, 3H, CH_3_).

#### Methyl 5-(3-Carbamoylphenyl)furan-2-carboxylate (**15**)

Procedure B. Starting compounds: methyl 5-bromofuran-2-carboxylate (400 mg, 2.0 mmol, **12**) and (3-carbamoylphenyl)boronic acid (429 mg, 2.6 mol). The crude was purified by flash column chromatography (DCM–EtOAc 7:3) to give the desired product as a white solid. Yield: 123 mg, 0.5 mmol, 25%. mp 192 °C. TLC (cyclohexane–EtOAc 4:6): *R*_f_ = 0.24. ^1^H NMR (300 MHz, CDCl_3_): δ (ppm) 8.22 (t, *J* = 1.4 Hz, 1H, H_7_), 7.94 (dt, *J* = 7.8, 1.4 Hz, 1H, H_9_), 7.79 (dt, *J* = 7.8, 1.4 Hz, 1H, H_10_), 7.52 (t, *J* = 7.8 Hz, 1H, H_11_), 7.26 (d partially hidden by solvent peak, *J* = 3.6 Hz, 1H, H_3_), 6.83 (d, *J* = 3.6 Hz, 1H, H_4_), 6.20 (bs exch. D_2_O, 1H, N*H*), 5.66 (bs exch. D_2_O, 1H, N*H*), 3.93 (s, 3H, CH_3_).

#### Methyl 5-(3-Cyanophenyl)furan-2-carboxylate (**16**)

Procedure B. Starting compounds: methyl 5-bromofuran-2-carboxylate (400 mg, 2.0 mmol, **12**) and (3-cyanophenyl)boronic acid (382 mg, 2.6 mmol). The crude was purified by flash column chromatography (cyclohexane–EtOAc 8:2) to give the desired product as a white solid. Yield: 345 mg, 1.5 mmol, 75%. mp 149 °C. TLC (cyclohexane–EtOAc 8:2): *R*_f_ = 0.33. ^1^H NMR (300 MHz, CDCl_3_): δ (ppm) 8.06 (s, 1H, H_7_), 8.00 (d, *J* = 7.8 Hz, 1H, H_11_), 7.62 (d, *J* = 7.8 Hz, 1H, H_9_), 7.54 (t, *J* = 7.8 Hz, 1H, H_10_), 7.26 (d partially hidden by solvent peak, *J* = 3.6 Hz, 1H, H_3_), 6.83 (d, *J* = 3.6 Hz, 1H, H_4_), 3.94 (s, 3H, CH_3_).

#### (5-(Methoxycarbonyl)furan-2-yl)boronic Acid (**17**)

Isopropylmagnesium chloride (2 M in THF, 2.4 mmol) was added to a solution of bis[2-(*N*,*N*-dimethylamino)ethyl] ether (62 mg, 2.4 mmol) in THF (10 mL) under a N_2_ atmosphere. The resulting mixture was stirred for 20 min at 10–15 °C before methyl 5-bromofuran-2-carboxylate (400 mg, 2.0 mmol, **12**) was added; the stirring was continued at room temperature for 30 min. Then, trimethyl borate (416 mg, 4.0 mmol) was added at 0 °C and the reaction mixture was stirred for 10 min. After quenching with diluted 1 M HCl, the reaction was extracted with EtOAc (3 × 4 mL) and the organic layers were washed with brine, dried over anhydrous Na_2_SO_4_, and evaporated *in vacuo*. The resulting brown solid was purified *via* crystallization from hexane and EtOAc to afford the desired product as a beige solid. Yield: 255 mg, 1.5 mmol, 75%. mp 128 °C (dec.). TLC (cyclohexane–EtOAc 8:2): *R*_f_ = 0.54. ^1^H NMR (300 MHz, CDCl_3_): δ (ppm) 7.19 (d, *J* = 3.5 Hz, 1H, H_3_), 7.08 (d, *J* = 3.5 Hz, 1H, H_4_), 3.91 (s, 3H, CH_3_).^12^

#### Methyl 5-(*m*-Tolyl)furan-2-carboxylate (**18**)

Procedure C. Starting compounds: (5-(methoxycarbonyl)furan-2-yl)boronic acid (221 mg, 1.3 mmol, **17**) and 1-bromo-3-methylbenzene (171 mg, 1.0 mol). The crude was purified by flash column chromatography (cyclohexane–EtOAc 8:2) to give the desired product as a white solid. Yield: 112 mg, 0.52 mmol, 52%. mp 76 °C. TLC (cyclohexane–EtOAc 8:2): *R*_f_ = 0.73. ^1^H NMR (300 MHz, CDCl_3_): δ (ppm) 7.62 (s, 1H, H_7_), 7.57 (d, *J* = 7.7 Hz, 1H, H_11_), 7.30 (t, *J* = 7.7 Hz, 1H, H_10_), 7.24 (d, *J* = 3.6 Hz, 1H, H_3_), 7.16 (d, *J* = 7.7 Hz, 1H, H_9_), 6.72 (d, *J* = 3.6 Hz, 1H, H_4_), 3.92 (s, 3H, OCH_3_), 2.40 (s, 3H, CH_3_).

#### Methyl 5-(3-(Methylcarbamoyl)phenyl)furan-2-carboxylate (**19**)

Procedure C. Starting compounds: (5-(methoxycarbonyl)furan-2-yl)boronic acid (221 mg, 1.3 mmol, **17**) and 3-bromo-*N*-methylbenzamide (213 mg, 1.0 mmol, **24**). The crude was purified by flash column chromatography (cyclohexane–EtOAc 5:5) to give the desired product as a yellow solid. Yield: 83 mg, 0.32 mmol, 32%. mp 76 °C. TLC (cyclohexane–EtOAc 5:5): *R*_f_ = 0.15. ^1^H NMR (300 MHz, CDCl_3_): δ (ppm) 8.11 (s, 1H, H_7_), 7.86 (d, *J* = 7.8 Hz, 1H, H_11_), 7.73 (d, *J* = 7.8 Hz, 1H, H_9_), 7.45 (t, *J* = 7.8 Hz, 1H, H_10_), 7.23 (d, *J* = 3.6 Hz, 1H, H_3_), 6.78 (d, *J* = 3.6 Hz, 1H, H_4_), 6.45 (bs exch. D_2_O, 1H, NH), 3.91 (s, 3H, OCH_3_), 3.03 (d, *J* = 4.6 Hz, 3H, NHCH_3_).

#### Methyl 5-(3-Sulfamoylphenyl)furan-2-carboxylate (**20**)

Procedure C. Starting compounds: (5-(methoxycarbonyl)furan-2-yl)boronic acid (221 mg, 1.3 mmol, **17**) and 3-bromobenzenesulfonamide (237 mg, 1.0 mmol). The crude was purified by flash column chromatography (cyclohexane–EtOAc 8:2) to give the desired product as a white solid. Yield: 217 mg, 0.77 mmol, 77%. mp 238 °C. TLC (cyclohexane–EtOAc 5:5): *R*_f_ = 0.38. ^1^H NMR (300 MHz, acetone-*d*_6_): δ (ppm) 8.32 (t, *J* = 1.8 Hz, 1H, H_7_), 8.06 (ddd, *J* = 7.8, 1.8, 1.1 Hz, 1H, H_9_), 7.91 (ddd, *J* = 7.8, 1.8, 1.1 Hz, 1H, H_11_), 7.69 (t, *J* = 7.8 Hz, 1H, H_10_), 7.36 (d, *J* = 3.7 Hz, 1H, H_3_), 7.21 (d, *J* = 3.7 Hz, 1H, H_4_), 6.73 (bs exch. D_2_O, 2H, SO_2_NH_2_), 3.90 (s, 3H, CH_3_).

#### Methyl 5-(3-(Methoxycarbonyl)phenyl)furan-2-carboxylate (**21**)

Procedure C. Starting compounds: (5-(methoxycarbonyl)furan-2-yl)boronic acid (221 mg, 1.3 mmol, **17**) and methyl 3-bromobenzoate (214 mg, 1.0 mmol). The crude was purified by flash column chromatography (cyclohexane–EtOAc 8:2) to give the desired product as a white solid. Yield: 125 mg, 0.48 mmol, 48%. mp 115 °C. TLC (cyclohexane–EtOAc 8:2): *R*_f_ = 0.51. ^1^H NMR (300 MHz, CDCl_3_): δ (ppm) 8.42 (s, 1H, H_7_), 8.04–7.98 (m, 2H, H_9,11_), 7.52 (t, *J* = 7.8 Hz, 1H, H_10_), 7.28 (d partially hidden by solvent peak, *J* = 3.6 Hz, 1H, H_3_), 6.84 (d, *J* = 3.6 Hz, 1H, H_4_), 3.96 (s, 3H, CH_3_), 3.93 (s, 3H, CH_3_).

#### Methyl 5-(3-Aminophenyl)furan-2-carboxylate (**22**)

To a solution of methyl 5-(3-nitrophenyl)furan-2-carboxylate (232 mg, 1.0 mmol, **23**) in EtOAc (4 mL), SnCl_2_ (57 mg, 0.3 mmol) was added, and the mixture was refluxed for 5 h. After quenching by addition of a saturated solution of NaHCO_3_ until pH 7–8, the precipitated tin salts were eliminated by filtration, and the aqueous phase was extracted with EtOAc (3 × 4 mL). The organic layer was dried over Na_2_SO_4_, filtered, and evaporated under vacuum. The crude residue was purified by flash column chromatography (cyclohexane–EtOAc 8:2) to provide the desired compound as a white solid. Yield: 165 mg, 0.76 mmol, 76%. mp 215 °C (dec.). TLC (cyclohexane–EtOAc 8:2): *R*_f_ = 0.45. ^1^H NMR (300 MHz, DMSO-*d*_6_): δ (ppm) 7.36 (d, *J* = 3.7 Hz, 1H, H_3_), 7.09 (t, *J* = 7.8 Hz, 1H, H_11_), 7.00 (t, *J* = 2.0 Hz, 1H, H_7_), 6.97 (d, *J* = 3.7 Hz, 1H, H_4_), 6.94 (d, *J* = 7.8 Hz, 1H, H_9_), 6.57 (dd, *J* = 7.8, 2.0 Hz, 1H, H_9_), 5.31 (bs exch. D_2_O, 2H, NH_2_), 3.81 (s, 3H, CH_3_).^26^

#### Methyl 5-(3-Nitrophenyl)furan-2-carboxylate (**23**)

Procedure D. Starting compound: 5-(3-nitrophenyl)furan-2-carboxylic acid (233 mg, 1.0 mmol). Light yellow solid. Yield: 215 mg, 0.87 mmol, 87%. mp = 143 °C. TLC (cyclohexane–EtOAc 8:2) *R*_f_ = 0.28. ^1^H NMR (300 MHz, CDCl_3_): δ (ppm) 8.59 (t, *J* = 2.0 Hz, 1H, H_7_), 8.20 (ddd, *J* = 8.0, 2.0, 1.0 Hz, 1H, H_11_), 8.11 (ddd, *J* = 8.0, 2.0, 1.0 Hz, 1H, H_9_), 7.62 (t, *J* = 8.0 Hz, 1H, H_10_), 7.29 (d, *J* = 3.6 Hz, 1H, H_3_), 6.91 (d, *J* = 3.6 Hz, 1H, H_4_), 3.95 (s, 3H, CH_3_).

#### 3-Bromo-*N*-methylbenzamide (**24**)

To a solution of 3-bromobenzoic acid (200 mg, 1.0 mmol) in THF (4 mL), HATU (570 mg, 1.5 mmol) and *N*,*N*-diisopropylethylamine (259 mg, 2.0 mmol) were added at 0 °C. The reaction mixture was stirred for 30 min at room temperature. Methylamine (2 M in THF, 2.0 mmol) was added, and the reaction was stirred for 18 h. After completion, the mixture was extracted with EtOAc (3 × 4 mL). The organic phase was dried over Na_2_SO_4_, filtered, and concentrated under reduced pressure. The resulting residue was purified by flash column chromatography (DCM–MeOH 97:3) to afford a grey solid. Yield: 151 mg, 0.71 mmol, 71%. mp 91 °C. TLC (DCM–MeOH 97:3): *R*_f_ = 0.33. ^1^H NMR (300 MHz, CDCl_3_): δ (ppm) 7.89 (s, 1H, H_2_), 7.66 (d, *J* = 7.8 Hz, 1H, H_6_), 7.56 (d, *J* = 7.8 Hz, 1H, H_4_), 7.23 (t, *J* = 7.8 Hz, 1H, H_5_), 6.80 (bs exch. D_2_O, 1H, NH), 2.95 (d, *J* = 4.7 Hz, 3H, CH_3_).^27^

### Production and Purification of MbtI for Crystallization Trials

*E. coli* BL21 cells were transformed with a pET-28a plasmid (GenScript, Piscataway, NJ, USA), bearing a codon-optimized open reading frame for MbtI. Two transformed colonies were added to a starter medium (9.2 mL 2YT medium pH 7.0, 200 μL of 40% glucose, 100 μL of 1 M MgSO_4_, 500 μL of 20X NPS buffer, and 20 μL of 50 mg/mL kanamycin) and stirred at 37 °C and 180 rpm for 7 h. Then, sterile 5 L Erlenmeyer flasks were filled with 1 L of 2YT medium pH 7.0, 50 mL of auto-induction supplement (0.5% glycerol, 0.05% glucose, 0.2% α-lactose, 25 mM (NH_4_)_2_SO_4_, 50 mM KH_2_PO_4_, 50 mM Na_2_HPO_4_, 1 mM MgSO_4_), and 2 mL of 50 mg/mL kanamycin; 1 mL of the starter suspension was added to each flask, and the culture was stirred at 180 rpm for 4 h at 37 °C, and 12 h at 25 °C.

After centrifugation, the cell pellet was resuspended using 50 mL of IMAC A solution (25 mM Tris·HCl pH 8.5, 300 mM NaCl, 25 mM imidazole), with the addition of 50 μL of Benzonase Nuclease (Sigma-Aldrich) and a tablet of EDTA-free protease inhibitor complex (cOmplete, Sigma-Aldrich). Then, the cells were lysed with a CF2 cell disruptor (Constant Systems Ltd., Daventry, UK) and centrifuged. The supernatant was charged on a 1 mL HisTrap high performance column (GE Healthcare, Chicago, IL, USA) and eluted with a gradient obtained by mixing IMAC A and IMAC B (25 mM Tris·HCl pH 8.5, 300 mM NaCl, 400 mM imidazole) solutions. Then, TEV protease (200 μL of a 10 mg/mL solution) was added to the protein solution to cleave the His-tag, along with 5 μL of 1 M DTT (final concentration ≈ 100 μM). The resulting mixture was dialyzed overnight at 4 °C in a solution containing 25 mM Hepes·NaOH pH 8.0, 150 mM NaCl, and 0.5 mM DTT. Subsequently, 40 mM imidazole was added to the dialyzed protein, and the solution was loaded on a Bio-Rad column charged with Ni-NTA resin to remove the His-tag. The column was washed with IMAC B, and the protein was concentrated to about 1 mL, using 20 mL Vivaspin 15R centrifugal concentrators equipped with a 10,000 Da cut-off filter (Sartorius, Göttingen, Germany). The supernatant was loaded on a HiLoad 16/600 Superdex 200 exclusion chromatography column (GE Healthcare), previously equilibrated with the eluent solution (25 mM Hepes·NaOH pH 8.0, 150 mM NaCl, 1% glycerol). The fractions containing the protein were collected and concentrated to allow the obtainment of the final purified protein solution at about 20 mg/mL concentration (calculated with a NanoDrop 1000, Thermo Fisher Scientific); the samples were flash-frozen with liquid nitrogen and stored at −80 °C.

### Crystallization of MbtI-**10**, MbtI–Mg^2+^, and MbtI–Ba^2+^ Complexes

The crystallization experiments were performed at 4 °C by the sitting drop vapor diffusion technique in 96-well plates according to established protocols at the Crystallography Core Facility of the Institut Pasteur.^28^ The trials were set up with a Mosquito crystal Nanoliter Protein Crystallization Robot (TTP Labtech, Melbourne, UK); the plates were stored in a Rock Imager 1000 (Formulatrix, Bedford, MA, USA) and visually checked through the dedicated image repository, following a specific timetable. The drops were obtained by mixing an equal amount of protein and reservoir solutions to a final volume of 400 nL; the reservoir contained 150 μL of the precipitant mixture.

A solution of freshly purified MbtI was concentrated to 20 mg/mL in the gel filtration buffer (25 mM Hepes·NaOH pH 8.0, 150 mM NaCl, 1% glycerol); the sodium salt of **10**, dissolved in H_2_O, was added to the protein to a final concentration of 5 mM and incubated at 4 °C overnight. Prism-shaped crystals of up to 320 × 160 × 30 μm grew within two weeks in the presence of 20% polyethylene glycol (PEG) 3350 and 0.2 M solutions of different sodium salts, among which sodium tartrate provided the best results. The previously described protocol was also applied for the obtainment of the MbtI–Mg^2+^ and MbtI–Ba^2+^ crystals. MbtI–Mg^2+^ crystals (tabular, maximum side length: 160 μm) grew within two weeks in the presence of 1.75 M MgSO_4_ and 0.1 M 2-(*N*-morpholino)ethanesulfonic acid (MES) pH 6.5, while MbtI–Ba^2+^ crystals (prism, maximum side length: 270 μm) grew within three weeks in the presence of 5 mM chorismic acid barium salt, 0.04 M KH_2_PO_4_, 16% PEG 8000, and 16% glycerol. The crystals were harvested with CryoLoops (Hampton Research, Aliso Viejo, CA, USA), cryoprotected in a 1:1 mixture of paraffin and parathon oil (Hampton Research) and flash-frozen by rapid immersion in liquid nitrogen.

### Data Collection and Structure Solution

Diffraction data were acquired at the SOLEIL Synchrotron (Saint-Aubin, France) on the beamlines PROXIMA-1 for the MbtI-**10** complex and PROXIMA-2A for MbtI–Mg^2+^ and MbtI–Ba^2+^, from crystals maintained at 100 K. The data were processed, scaled, and analyzed using XDSME and autoPROC.^29,30^ The structures were solved with the molecular replacement method through the program PHASER,^31^ available in the CCP4 suite,^32^ using the PDB-deposited structure 3RV7 model.^20^ The geometrical restraints for the inhibitor **10** were generated with the Grade server (http://grade.globalphasing.org), while restraints for the salicylate were obtained from AceDRG.^33^ All rebuilding and adjustments of the models were performed with COOT.^34^ The refinement was carried out with BUSTER,^35^ applying local structure similarity restraints for noncrystallography symmetry and a translation–libration–screw model. The final validation was performed with MOLPROBITY and PHENIX.^36,37^ Data collection, refinement, and model statistics are summarized in Table 2. Graphical representations were rendered with Pymol.^38^

### MbtI Enzymatic Assays

MbtI was produced in a recombinant form and purified, as previously reported.^12^ The enzyme activity was determined by a fluorimetric assay, performed in a final volume of 400 μL at 37 °C, in 50 mM Hepes pH 7.5, 5 mM MgCl_2_, and 1–2 μM MbtI. The reactions were started by the addition of chorismic acid and monitored using a PerkinElmer LS3 fluorimeter (Ex. λ = 305 nm, Em. λ = 420 nm). Initial inhibition assays were carried out in the presence of 100 μM of each compound [stock solution 20 mM in dimethyl sulfoxide (DMSO)] and chorismic acid at a final concentration of 50 μM. For the most potent inhibitors, the IC_50_ and *K*_i_ were determined. For IC_50_ determinations, the enzyme activity was measured at different compound concentrations, and the values were calculated according to the eq 1, with Origin 8 software
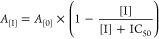
1where *A*_[I]_ is the activity of the enzyme at inhibitor concentration [I] and *A*_[0]_ is the activity of the enzyme without inhibitor.

The *K*_i_ was determined at different substrate [S] and compound concentrations using eq 2
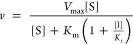
2

To verify that the compounds were not PAINS, the inhibition was tested in the presence of 0.1 mg/mL of BSA or 0.01% (v/v) Triton X-100 to confirm that they did not act as aggregators, and with 100 mM of DTT, to exclude an inhibition because of reaction with cysteines.^40^

### Minimal Inhibitory Concentration Determinations and Siderophore Production Assay

The minimal inhibitory concentration MIC^99^ of active compounds against *M. tuberculosis* H37Rv was determined on Middlebrook 7H11 agar solid medium, as previously reported.^11^ Additionally, MIC^99^ against *M. bovis* BCG was determined in low-iron chelated Sauton’s medium by the resazurin reduction assay method.^41,42^

The siderophore activity was measured by the Universal CAS liquid assay.^16^*M. bovis* cells were grown in 7H9 medium, subcultured in chelated Sauton’s medium, and finally diluted to an optical density (OD_600_) of 0.01 in chelated Sauton’s containing different concentrations of compounds in 96-well plates. After 15 days of incubation at 37 °C, cells were harvested. Supernatants were used to perform the CAS assay, while cell pellets were used for the determination of mycobactins. For the CAS assay, 100 μL of supernatant were mixed with 100 μL of CAS assay liquid solution in a 96-well plate, incubated 10 min at room temperature, and absorbance was read at 630 nm. The siderophore units were calculated using eq 3
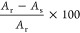
3where *A*_r_ is the absorbance at 630 nm of the blank medium with CAS assay solution and *A*_s_ is the absorbance of the culture supernatants with CAS assay solution.

For mycobactin determination, cell pellets were extracted in EtOH overnight, and then, 0.1 M FeCl_3_ in EtOH was added until no color change was observed. The mixture was incubated at room temperature for 1 h. Mycobactins were extracted in CHCl_3_, washed with H_2_O, and evaporated; then, the residue was dissolved in MeOH. The concentration of mycobactins was determined by measuring the absorbance at 450 nm (1% solution of mycobactins gives an absorbance of 42.8).

### Salicylic Acid Determination in MbtI Purified Protein

To confirm the presence of salicylic acid in complex with the purified MbtI, the protein was concentrated to 25 mg/mL and denatured by incubation at 95 °C for 10 min to liberate any bound ligand. After 15 min of centrifugation at 12,000 rpm, the supernatant was recovered and analyzed in UHPLC/MS. The chromatographic analysis was performed with a UHPLC apparatus JASCO X-LC system (Easton, MD, USA), coupled with a MS spectrometer Thermo Fisher Scientific LTQ XL ESI-MS/MS system. Chromatography was performed on an Acquity column (Waters Corporation, Milford, MA, USA) of 3 μm particle size, 0.3 mL/min, gradient 10 min from 90:10 H_2_O/acetonitrile (MeCN) to 100% MeCN and then 4 min in 100% MeCN. Run were also recorded at 220 nm. The analyses were performed in full-scan from 120 and 2000 amu, negative mode with [M – H]^−^ at 137.11 amu, and base peaks were analyzed with a dependent scan method with collision-induced dissociation (CID) = 30 eV in order to confirm the structure. As a positive control, a solution of salicylic acid (1 mg/mL, Sigma-Aldrich) was used, while the completely unrelated protein pantothenate kinase, expressed in the same *E. coli* strain and purified using a similar protocol to that of MbtI, was employed as negative control.

### Docking Studies

Compounds **10** and **11** were docked into the minimized average structure of MbtI, complexed with the lead **I** in the absence of the Mg^2+^ ion.^11^ The software Gold with ChemScore fitness function was used.^43^ The docking site was defined as the region comprising all residues that stayed within 10 Å from the reference compound **I**. The best docking poses were taken into consideration and subjected to MD simulations.

### MD Simulations

All simulations were performed using AMBER 16.^44^ General Amber force field (GAFF) parameters were assigned to the ligands, whereas partial charges were determined using the AM1-BCC method, as implemented in the antechamber suite of AMBER 16. MD simulations were carried out employing the ff14SB force field at 300 K. The MbtI-**10** complex was placed in a rectangular parallelepiped water-box and solvated with a 20 Å water cap by using the transferable intermolecular potential with 3 points (TIP3P) explicit solvent model. Sodium ions were added as counterions in order to neutralize the system. Before MD simulations, two steps of minimization were performed; in the first stage, a position constraint of 500 kcal/(mol·Å^2^) was applied to keep the protein fixed, thus minimizing only water molecules. In the second stage, the whole system was energy-minimized through 5000 steps of steepest descent followed by conjugate gradient (CG), until a convergence of 0.05 kcal/(mol·Å^2^) and imposing a harmonic potential of 10 kcal/(mol·Å^2^) to the protein α carbon. Particle mesh Ewald (PME) electrostatics and periodic boundary conditions were used in the simulations. The time step of the simulations was 2 fs with a cutoff of 10 Å for the nonbonded interactions, while SHAKE algorithm was applied to keep all bonds involving hydrogen atoms fixed. Constant-volume periodic boundary MD simulation was carried out for the first 0.5 ns, during which the temperature of the system was raised from 0 to 300 K. Then, 110 ns of constant-pressure periodic boundary MD was performed at 300 K, using the Langevin thermostat in order to maintain the temperature of the system constant. A harmonic force constraint of 10 kcal/(mol·Å^2^) was applied to the protein α carbons during the first 10 ns, whereas in the last 100 ns, no restraints were applied to the system. All the obtained MD trajectories were analyzed using the Cpptraj program implemented in AMBER 16.^44^

## Supporting Information Available

Additional synthetic schemes related to the synthesis of compounds **5** and **11**; ^1^H NMR, ^13^C NMR, FT-IR, and HRMS spectra of final compounds (**2–11**); ^1^H NMR spectra of intermediates (**12–24**); and crystallographic and computational supplementary figures (PDF)
Molecular formula strings (SMILES) of final compounds (CSV)

## Accession Codes

PDB codes for the crystal structures of MbtI with **10**, Mg^2+^ and Ba^2+^ are 6ZA4, 6ZA5 and 6ZA6, respectively. Authors will release the atomic coordinates upon article publication.

## Abbreviations

Mtb: *Mycobacterium tuberculosis*
MbtI: *Mycobacterium tuberculosis* salicylate synthase
SAR: structure–activity relationships
HTS: high-throughput screening
PAIN: pan-assay interference compound
BSA: bovine serum albumin
MRC-5: normal human fetal lung fibroblast cell line
MST: menaquinone-siderophore-tryptophan
ASU: asymmetric unit
DMF: *N*,*N*-dimethylformamide
HATU: 1-[bis(dimethylamino)methylene]-1*H*-1,2,3-triazolo[4,5-*b*]pyridinium 3-oxide hexafluorophosphate
THF: tetrahydrofuran
DCM: dichloromethane
PEG: polyethylene glycol
MES: 2-(*N*-morpholino)ethanesulfonic acid
DMSO: dimethyl sulfoxide
DTT: 1,4-dithio-dl-threitol
BCG: bacillus Calmette–Guerin
OD: optical density
CAS: chrome azurol S
CID: collision-induced dissociation
TIP3P: transferable intermolecular potential with 3 points
amu: atomic mass unit
MeCN: acetonitrile
PME: particle mesh Ewald
GAFF: general Amber force field
CG: conjugate gradient
MD: molecular dynamics
