# Supplementary material for: Shedding X-ray Light on the Role of Magnesium in the Activity of Mycobacterium tuberculosis Salicylate Synthase (MbtI) for Drug Design
Source: J Med Chem. 2020 Jun 12;63(13):7066–80. doi: 10.1021/acs.jmedchem.0c00373 (PMC8008425; doi:10.1021/acs.jmedchem.0c00373)
Supplement: Supplementary file 1 — jm0c00373_si_001.pdf [file jm0c00373_si_001.pdf]

Supporting information

Shedding X-Ray Light on the Role of Magnesium in the Activity of *M. tuberculosis* Salicylate Synthase (MbtI) for Drug Design

Matteo Mori[a§], Giovanni Stelitano[b§], Arianna Gelain[a], Elena Pini[a], Laurent R. Chiarelli[b], José C. Sammartino[b], Giulio Poli[c], Tiziano Tuccinardi[c,d], Giangiacomo Beretta[e], Alessio Porta[f], Marco Bellinzoni[g], Stefania Villa[a*], Fiorella Meneghetti[a]*

[a] Dipartimento di Scienze Farmaceutiche, Università degli Studi di Milano, via L. Mangiagalli 25, 20133 Milano; Italy.

[b] Dipartimento di Biologia e Biotecnologie "Lazzaro Spallanzani", Università degli Studi di Pavia, via Ferrata 9, 27100 Pavia; Italy.

[c] Dipartimento di Farmacia, Università di Pisa, via Bonanno 6, 56126 Pisa; Italy.

[d] Sbarro Institute for Cancer Research and Molecular Medicine, Center for Biotechnology, College of Science and Technology, Temple University, Philadelphia, PA 19122, USA.

[e] Dipartimento di Scienze e Politiche Ambientali, Università degli Studi di Milano, via Celoria 2, 20133 Milano; Italy.

[f] Dipartimento di Chimica, Università degli Studi di Pavia, viale Taramelli 12, 27100 Pavia, Italy.

[g] Unité de Microbiologie Structurale, Institut Pasteur, CNRS, Université de Paris, F-75724 Paris, France.

* Correspondence: marco.bellinzoni@pasteur.fr; stefania.villa@unimi.it.

[§] These authors equally contributed.

Table of Contents

S1. Synthetic schemes S3

S2. Analytical data for all synthesized compounds S4

S3. Crystal structures S31

S4. Computational simulation S35

S5. Mass spectrometry analysis S36

S6. SMILES codes S37

S1. Synthetic schemes

S1.1. Scheme 1

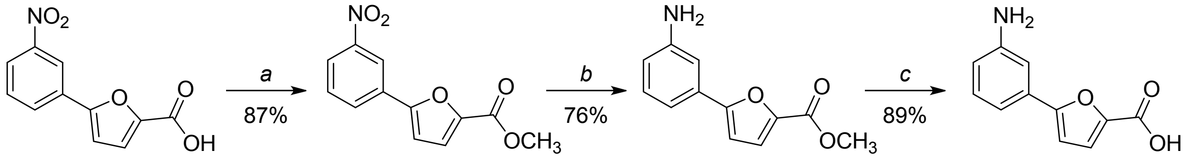

23 **22** **5**

Reagents and conditions: *a)* conc. H_2SO_4, MeOH, reflux, 24 h; *b*) $SnCl_2$, EtOAc, 5 h, reflux; *c*) NaOH, EtOH/THF 1:1, 5 h, reflux.

S1.2. Scheme 2

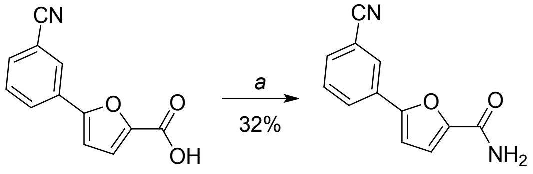

10 **11**

Reagents and conditions: *a*) *1*. HATU, DIPEA, DMF, r.t., 30 min.; *2*. NH_4Cl, r.t., 2 h.

S2. Analytical data for all synthesized compounds

S2.1. 5-(3-Chlorophenyl)furan-2-carboxylic acid (2)

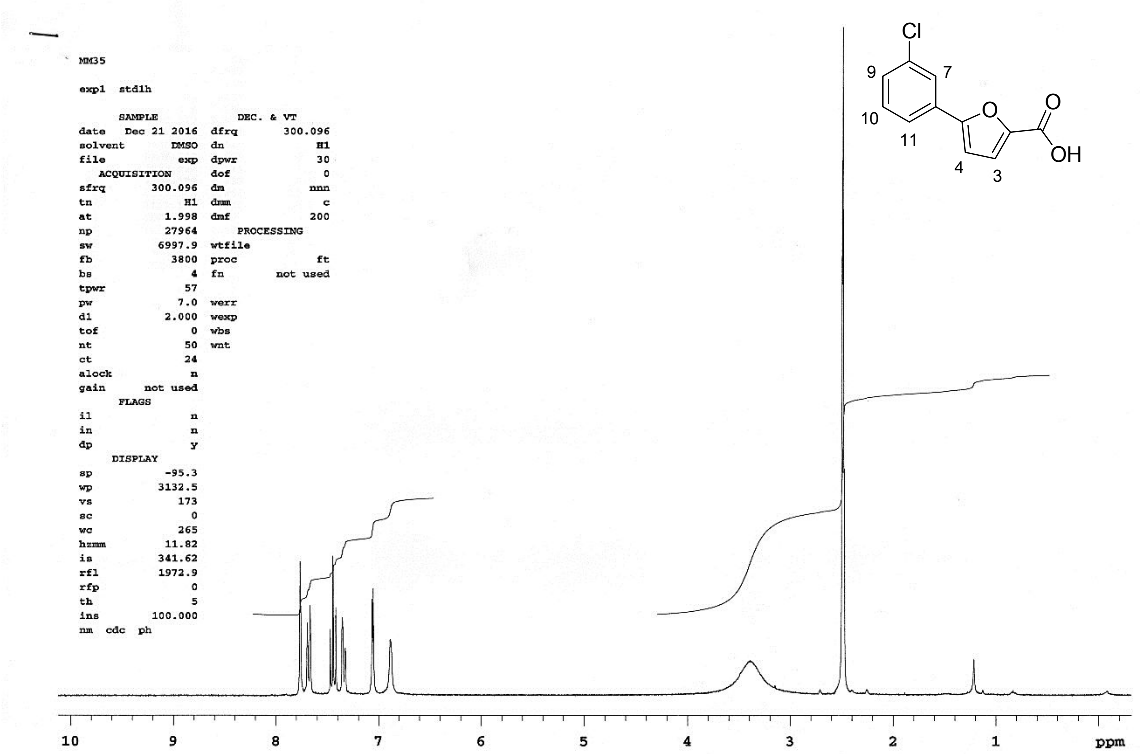

Figure S1. ^{1}H NMR spectrum of **2**.

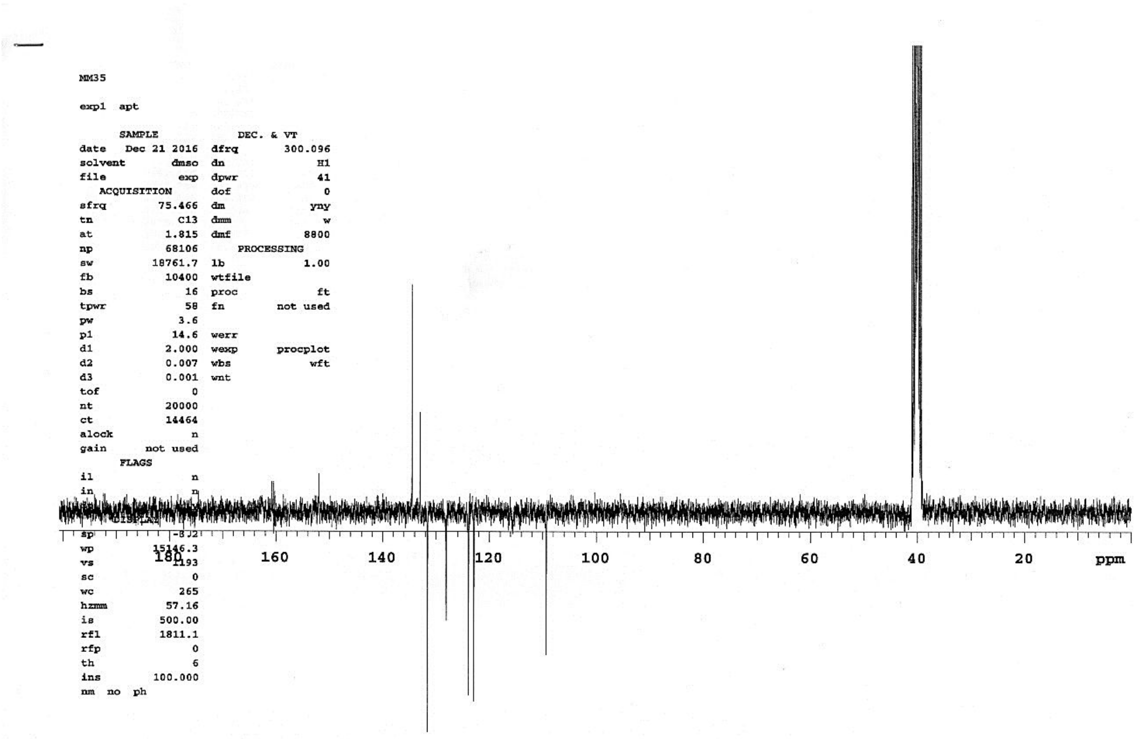

Figure S2. ^{13}C NMR spectrum of **2**.

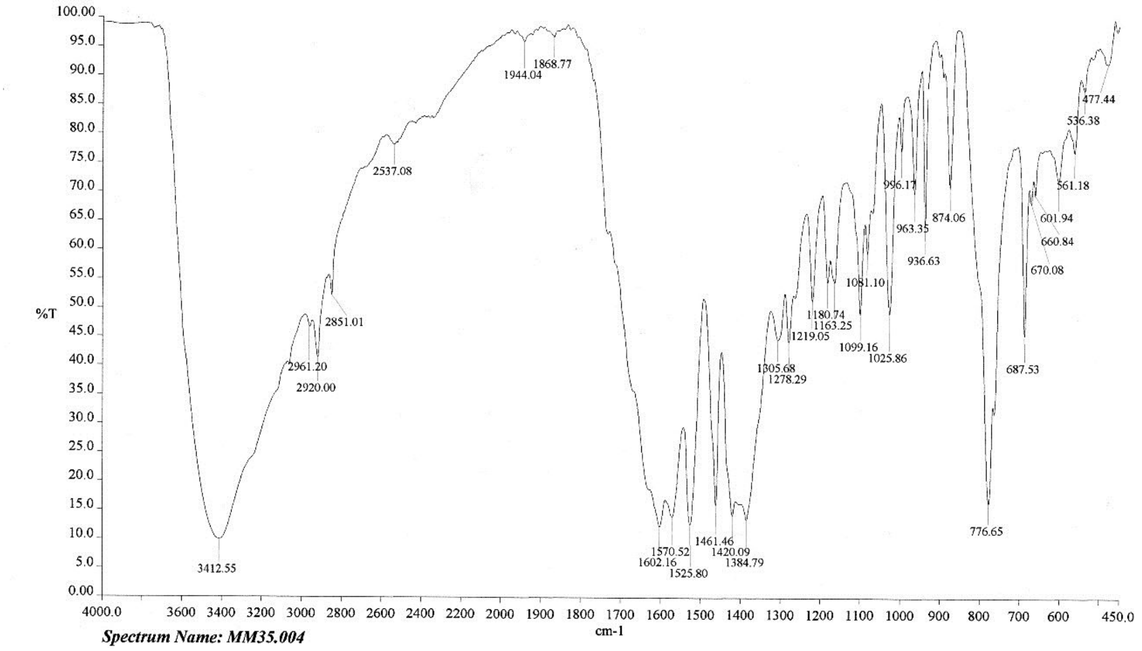

Figure S3. FT-IR spectrum of **2**.

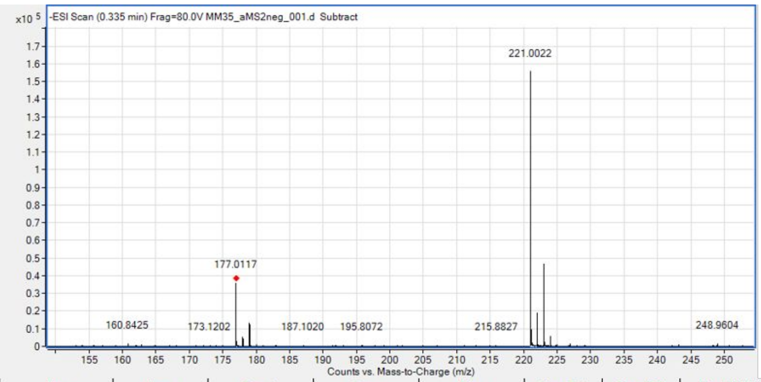

Best	Name	Formula	Score	Mass	Mass (Tgt)	Mass (DB)	Mass (MFG)	Diff (ppm)	Diff (abs. ppm)	Diff (mDa)	RT
TRUE		C11 H7 Cl O3	93.19	222.0094			222.0084	-4.79	4.79	-1.06	

Species	Ion Formula	m/z	Height	Score (MFG)	Score (MS)	Score (MFG, MS/MS	Score (mass)	Score (iso. abund)	Score (iso. spacing)
(M-H)-	C11 H6 Cl O3	221.0011	177531.3	93.19	93.19		89.78	94.76	98.13

m/z	m/z (Calc)	Diff (ppm)	Diff (mDa)	Height	Height (Calc)	Height %	Height % (Calc)	Height Sum %	Height Sum% (Calc)
221.0022	221.0011	-5.21	-1.2	181750.5	177531.3	100	100	68.4	66.8
222.0052	222.0045	-3.06	-0.7	22055.9	21446.8	12.1	12.1	8.3	8.1
222.9994	222.9985	-4.17	-0.9	54519.6	59078.2	30	33.3	20.5	22.2
224.0025	224.0017	-3.34	-0.7	6509.5	7033.1	3.6	4	2.4	2.6
225.0064	225.0038	-11.61	-2.6	992.3	738.5	0.5	0.4	0.4	0.3

Figure S4. HRMS spectrum and data of **2**.

S2.2. 5-(3-Hydroxyphenyl)furan-2-carboxylic acid (3)

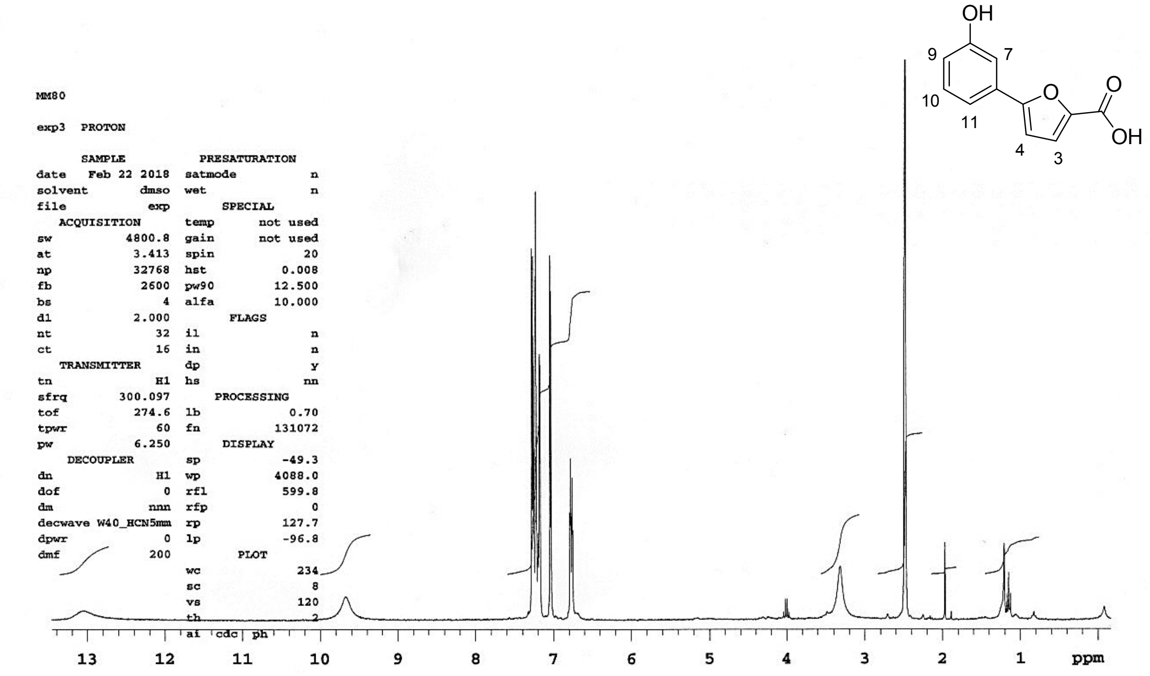

Figure S5. ^{1}H NMR spectrum of **3**.

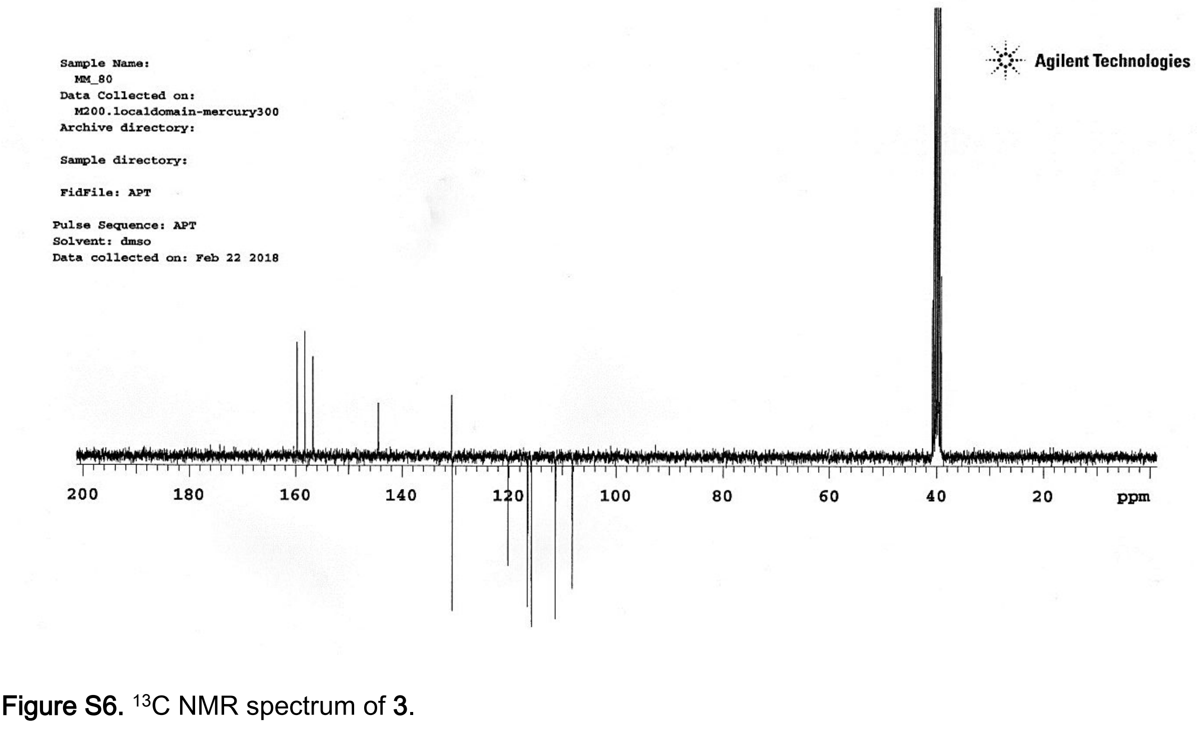

Figure S6. ^{13}C NMR spectrum of **3**.

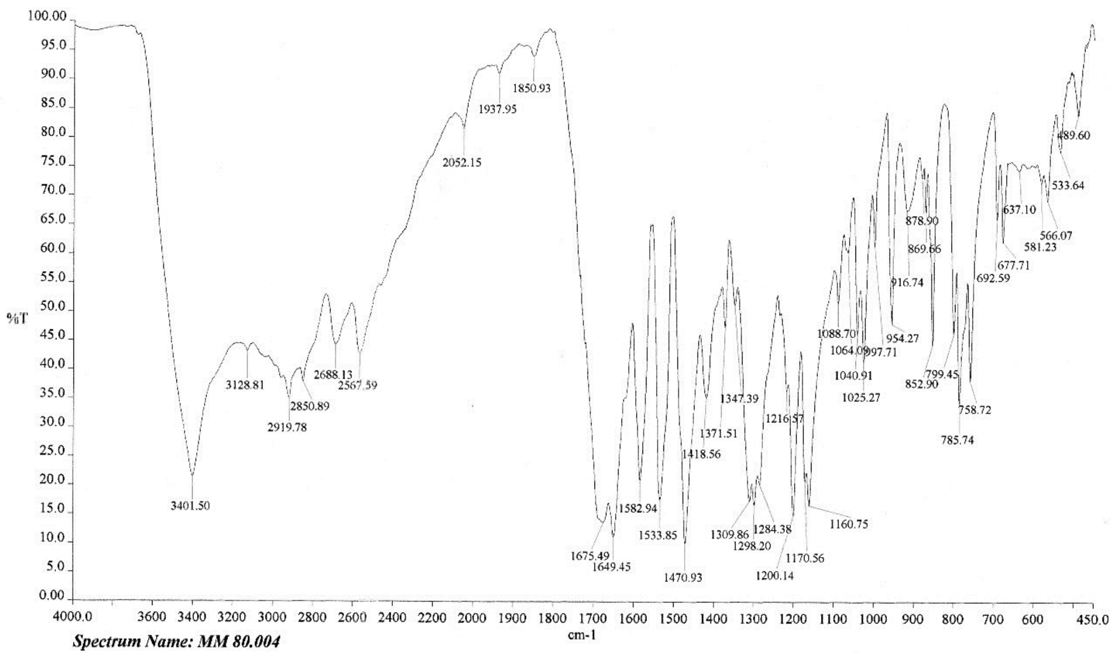

Figure S7. FT-IR spectrum of 3.

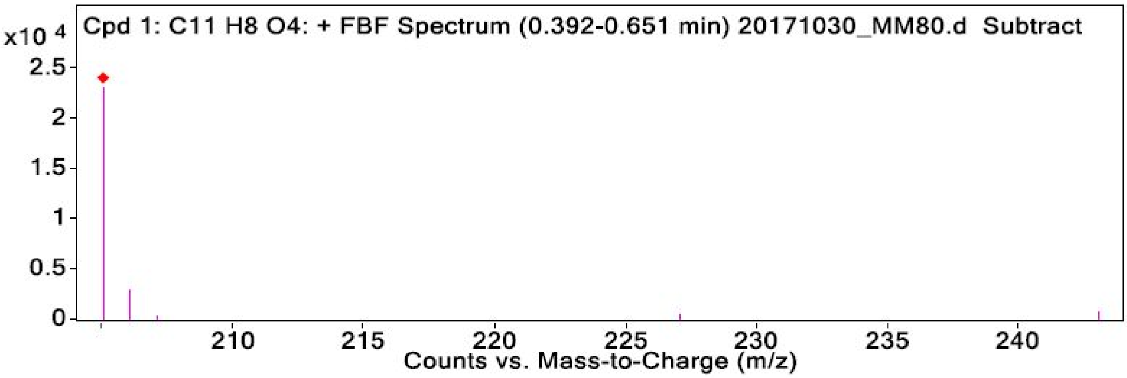

Peak List

m/z	z	Abund	Formula	Ion
205.0499	1	23191.3	C11H9O4	(M+H)+
206.0528	1	3034.29	C11H9O4	(M+H)+
207.0532	1	438.09	C11H9O4	(M+H)+
227.0337	1	578.59	C11H8NaO4	(M+Na)+
242.9963	1	835.95	C11H8KO4	(M+K)+

Figure S8. HRMS spectrum and data of 3.

S2.3. 5-(*m*-Tolyl)furan-2-carboxylic acid (4)

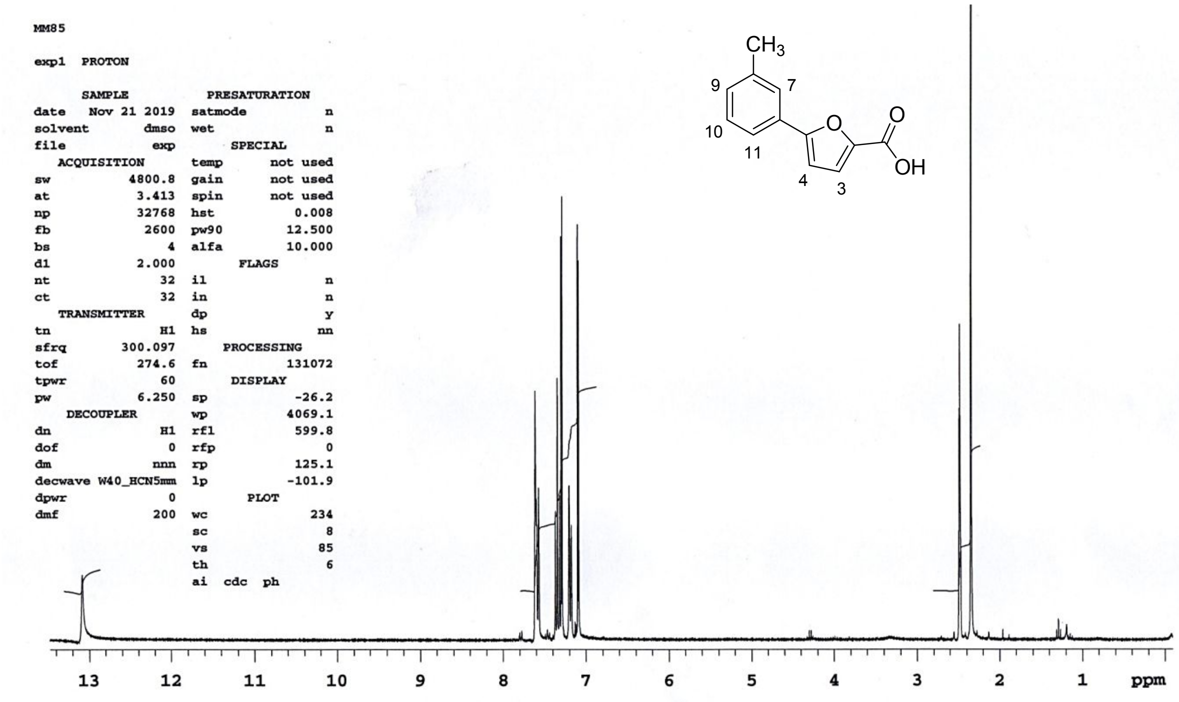

Figure S9. ^{1}H NMR spectrum of **4**.

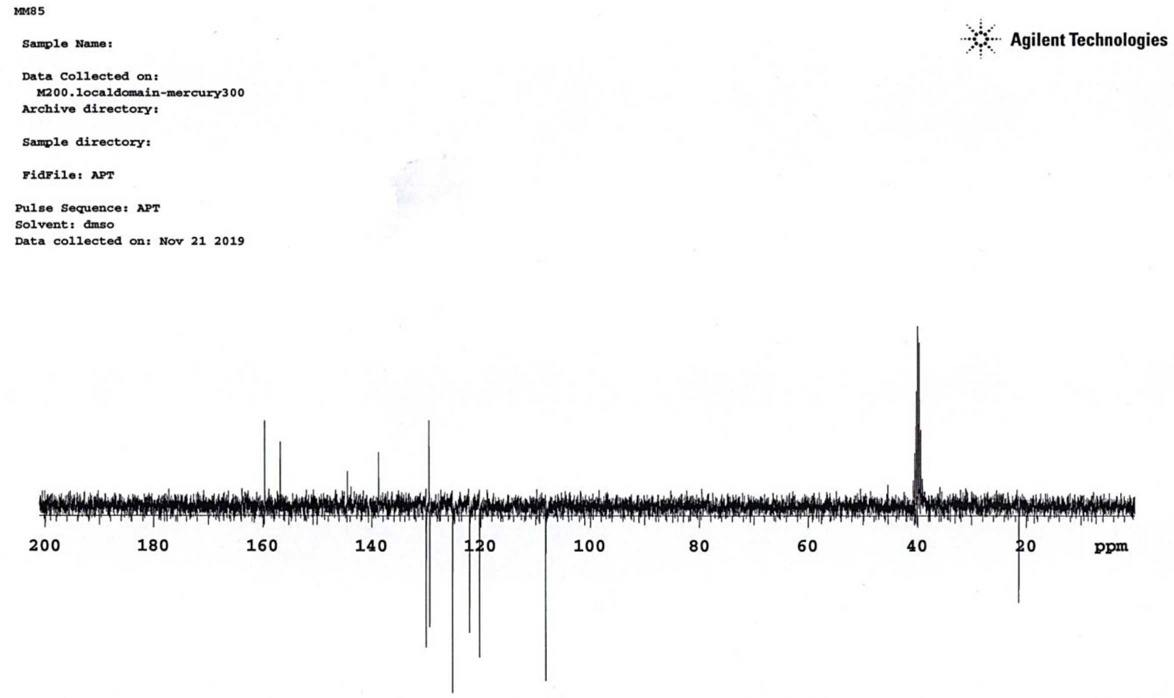

Figure S10. ^{13}C NMR spectrum of **4**.

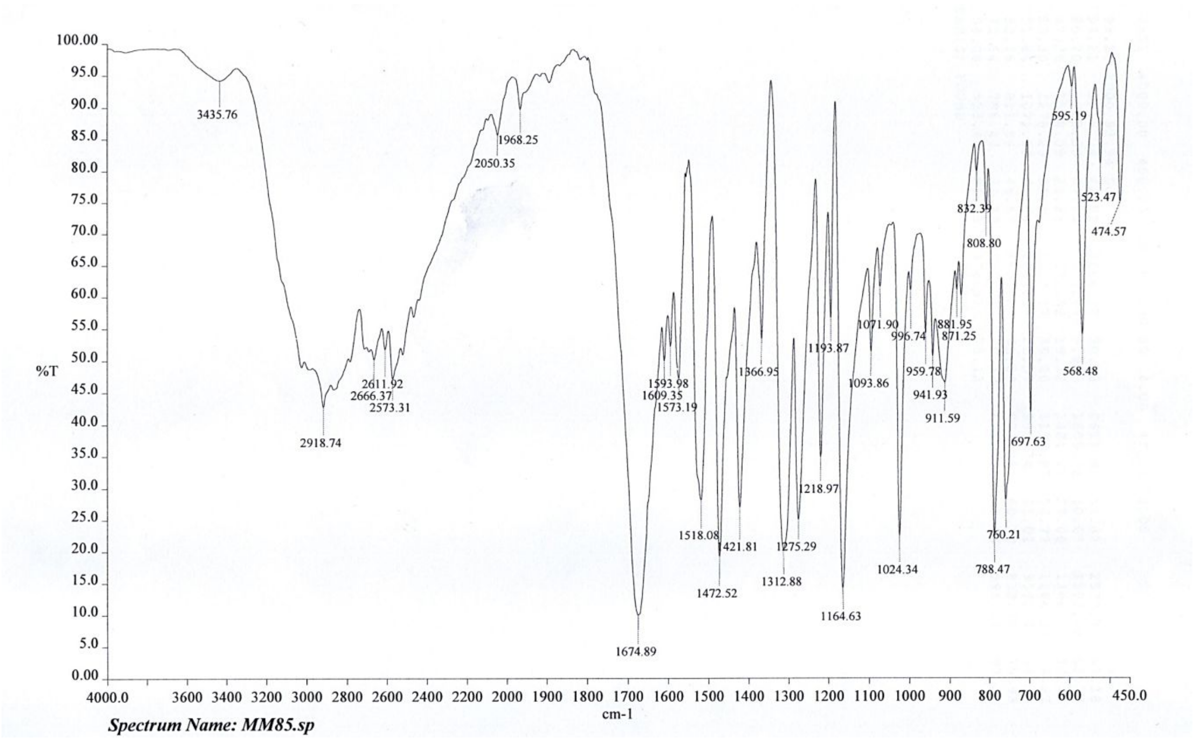

Figure S11. FT-IR spectrum of **4**.

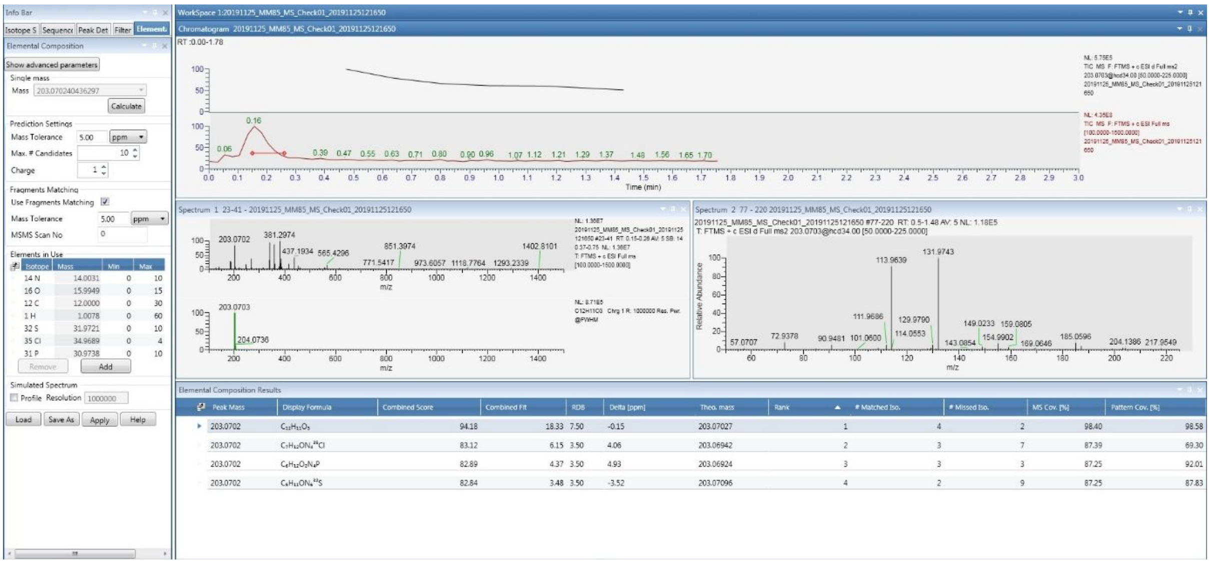

Figure S12. HRMS spectrum and data of **4**.

S2.4. 5-(3-Aminophenyl)furan-2-carboxylic acid (5)

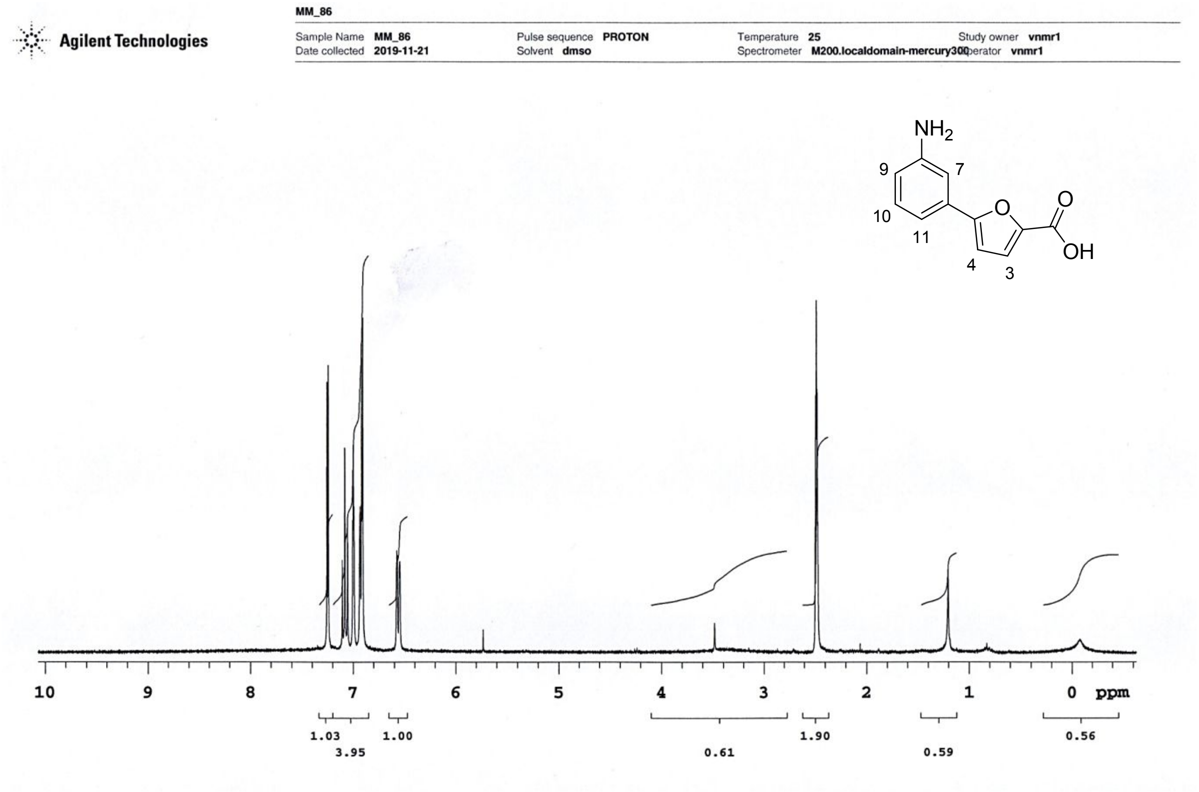

Figure S13. ^{1}H NMR spectrum of **5**.

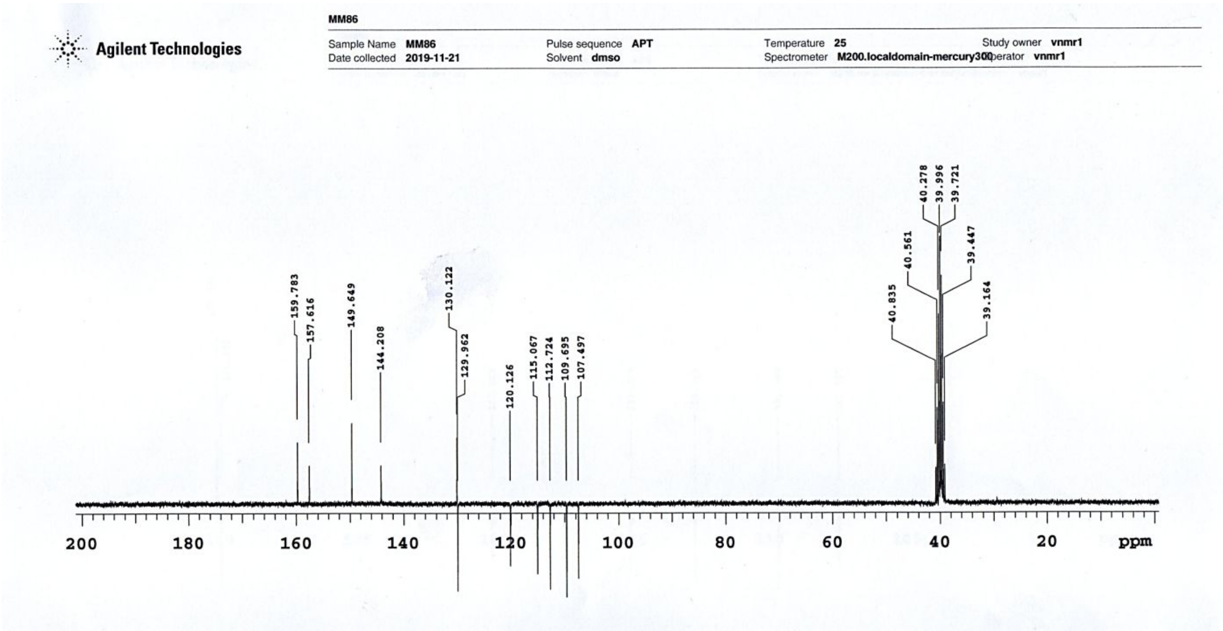

Figure S14. ^{13}C NMR spectrum of **5**.

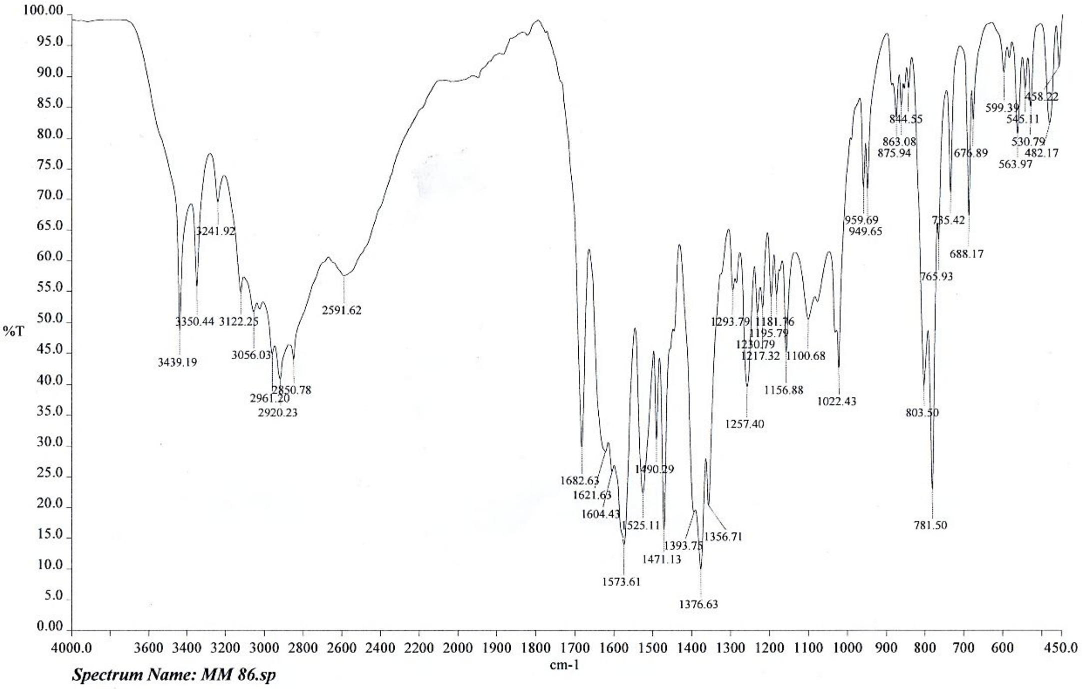

Figure S15. FT-IR spectrum of **5**.

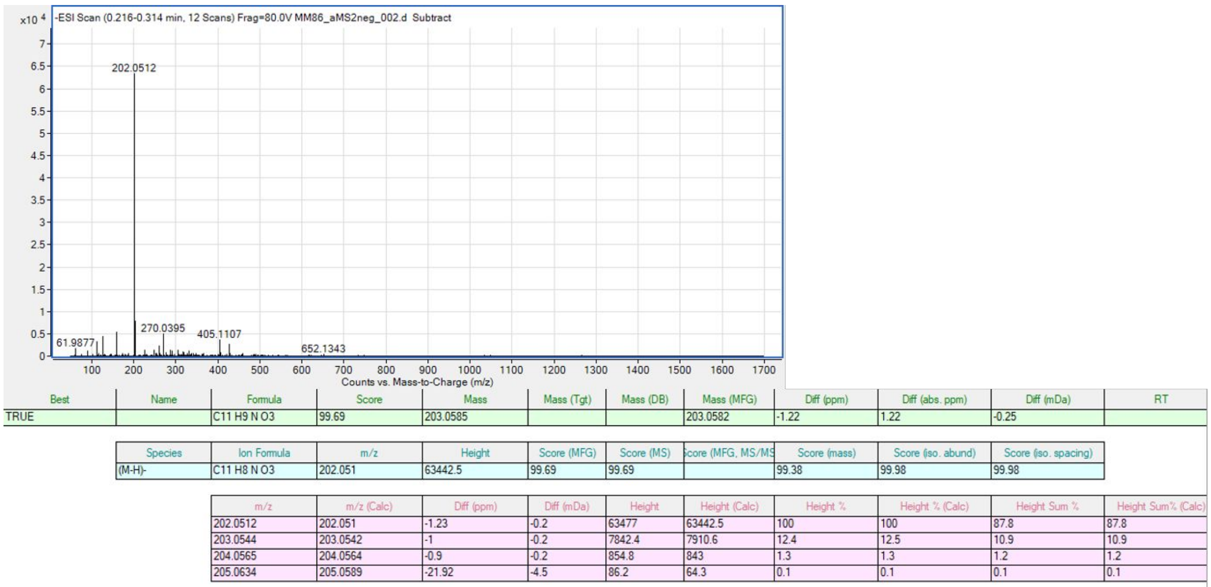

Best	Name	Formula	Score	Mass	Mass (Tgt)	Mass (DB)	Mass (MFG)	Diff (ppm)	Diff (abs. ppm)	Diff (mDa)	RT
TRUE		C11 H9 N O3	99.69	203.0585			203.0582	-1.22	1.22	-0.25	

Species	Ion Formula	m/z	Height	Score (MFG)	Score (MS)	Score (MFG, MS/MS	Score (mass)	Score (iso. abund)	Score (iso. spacing)
(M-H)-	C11 H8 N O3	202.051	63442.5	99.69	99.69		99.38	99.98	99.98

m/z	m/z (Calc)	Diff (ppm)	Diff (mDa)	Height	Height (Calc)	Height %	Height % (Calc)	Height Sum %	Height Sum% (Calc)
202.0512	202.051	-1.23	-0.2	63477	63442.5	100	100	87.8	87.8
203.0544	203.0542	-1	-0.2	7842.4	7910.6	12.4	12.5	10.9	10.9
204.0565	204.0564	-0.9	-0.2	854.8	843	1.3	1.3	1.2	1.2
205.0634	205.0589	-21.92	-4.5	86.2	64.3	0.1	0.1	0.1	0.1

Figure S16. HRMS spectrum and data of **5**.

S2.5. 5-(3-Carbamoylphenyl)furan-2-carboxylic acid (6)

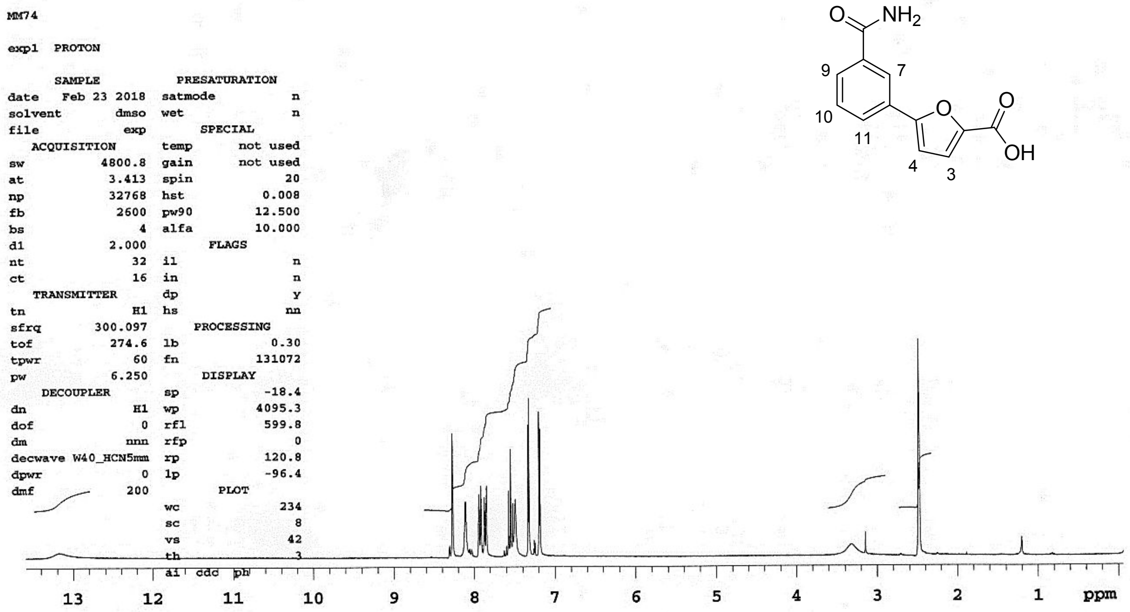

Figure S17. ^{1}H NMR spectrum of **6**.

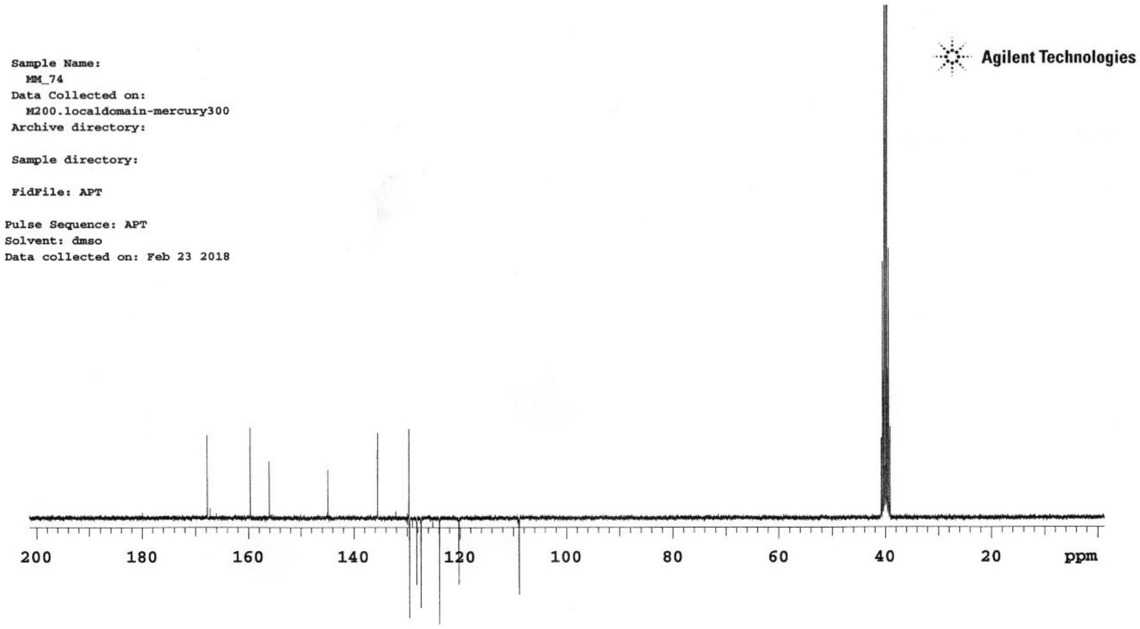

Figure S18. ^{13}C NMR spectrum of **6**.

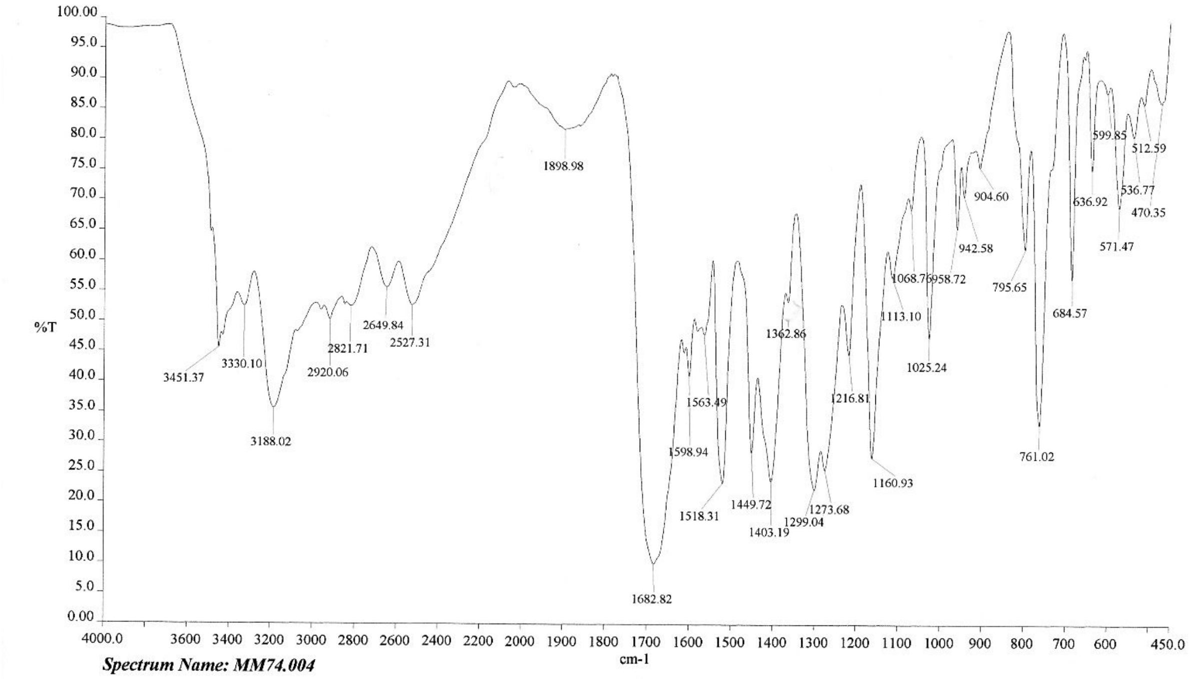

Figure S19. FT-IR spectrum of **6**.

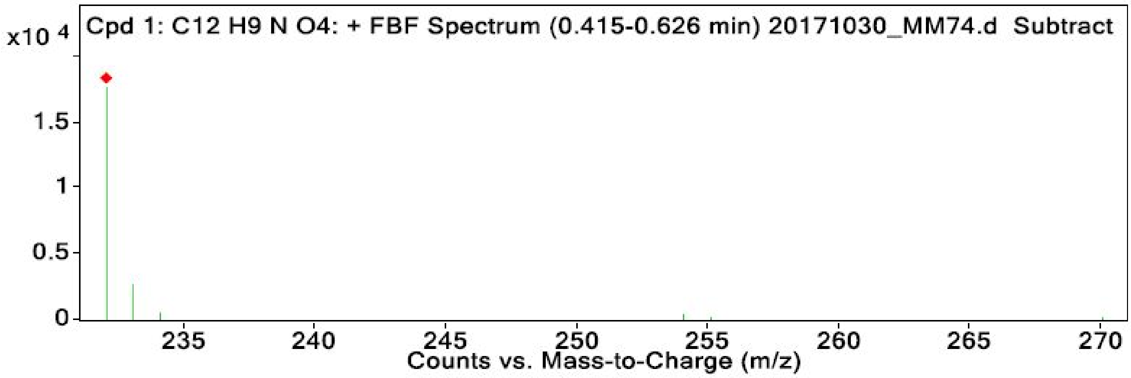

Peak List

m/z	z	Abund	Formula	Ion
232.0614	1	17701.73	C12H10NO4	(M+H)+
233.0621	1	2732.1	C12H10NO4	(M+H)+
234.061	1	523.11	C12H10NO4	(M+H)+
254.0419	1	481.04	C12H9NNaO4	(M+Na)+
255.0524	1	191.91	C12H9NNaO4	(M+Na)+
270.0063	1	167.86	C12H9KNO4	(M+K)+

Figure S20. HRMS spectrum and data of **6**.

S2.6. 5-(3-(Methylcarbamoyl)phenyl)furan-2-carboxylic acid (7)

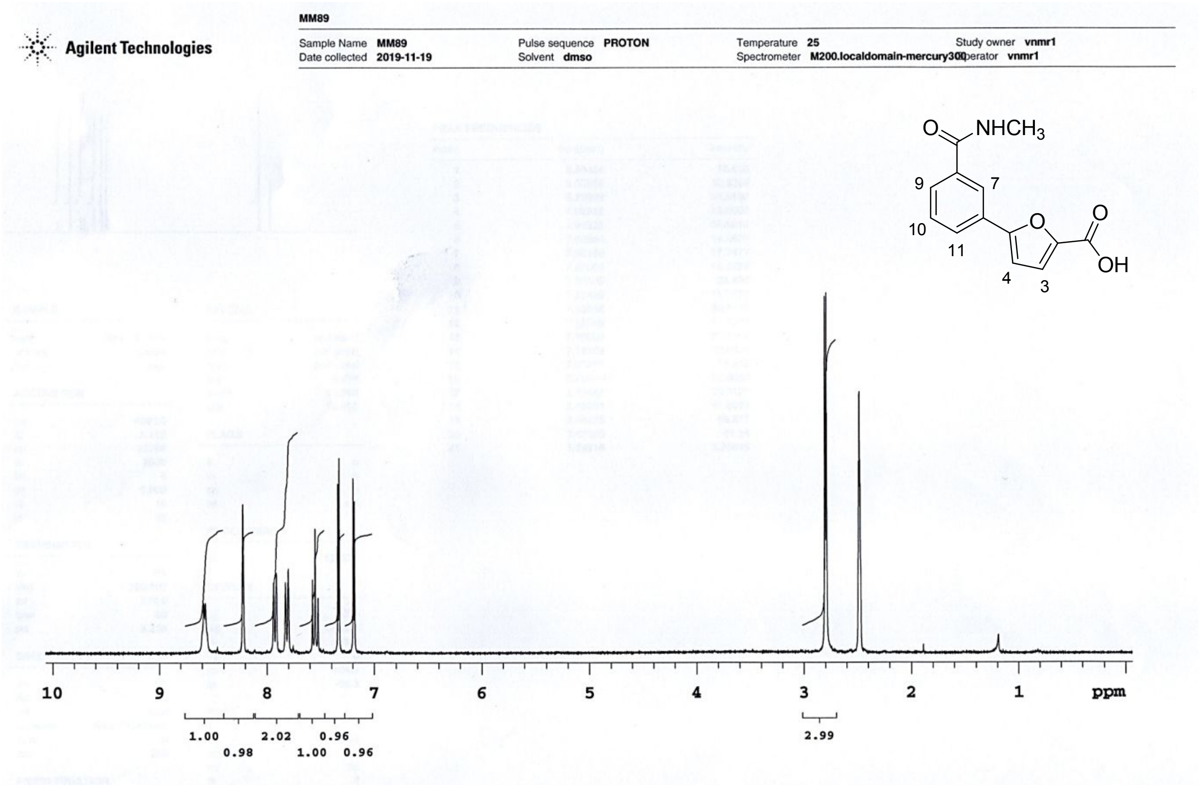

Figure S21. ^{1}H NMR spectrum of **7**.

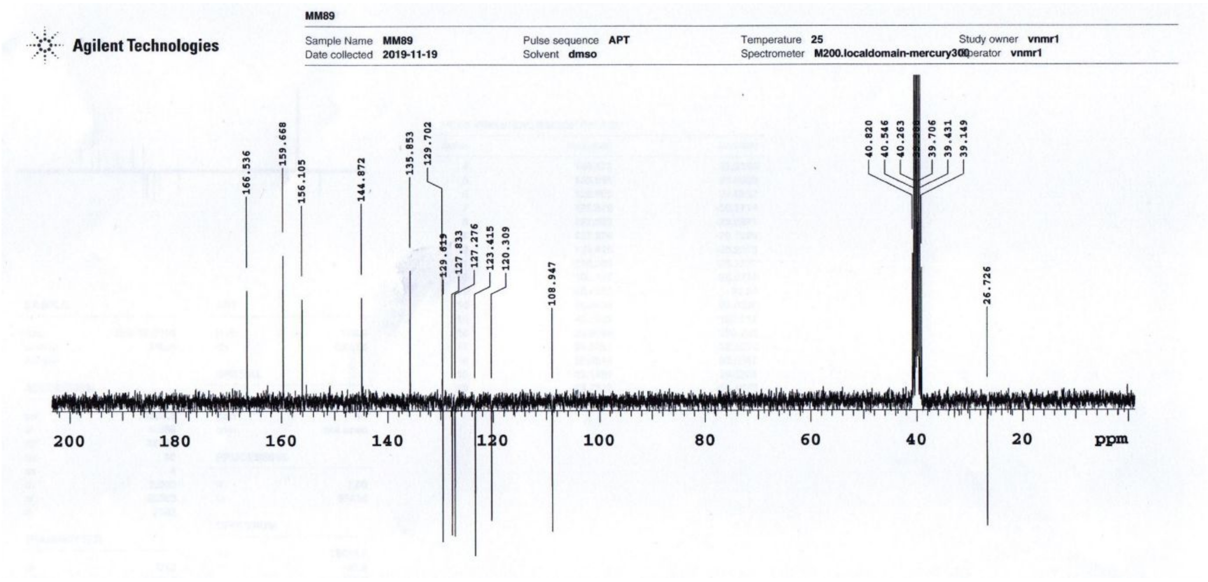

Figure S22. ^{13}C NMR spectrum of **7**.

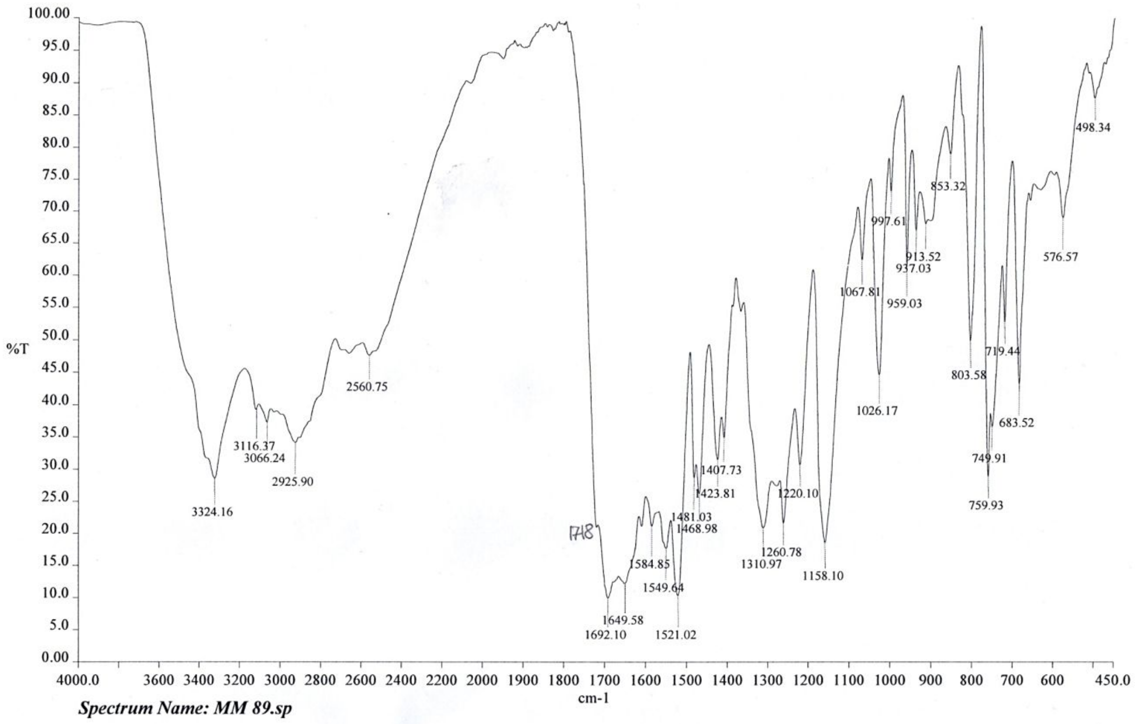

Figure S23. FT-IR spectrum of **7**.

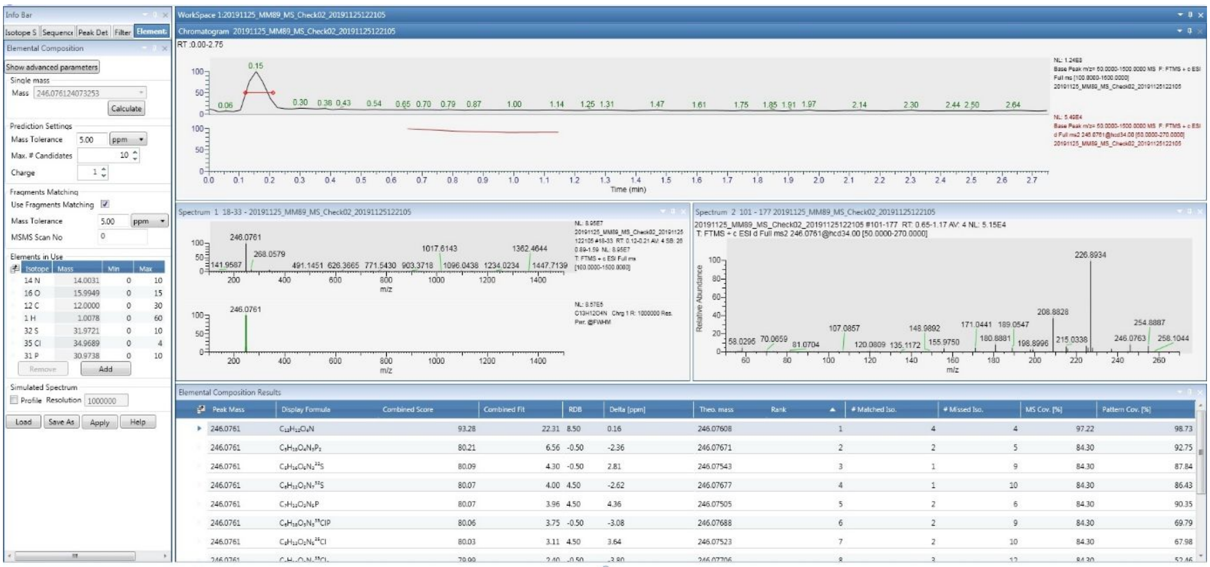

Figure S24. HRMS spectrum and data of **7**.

S2.7. 5-(3-Sulfamoylphenyl)furan-2-carboxylic acid (8)

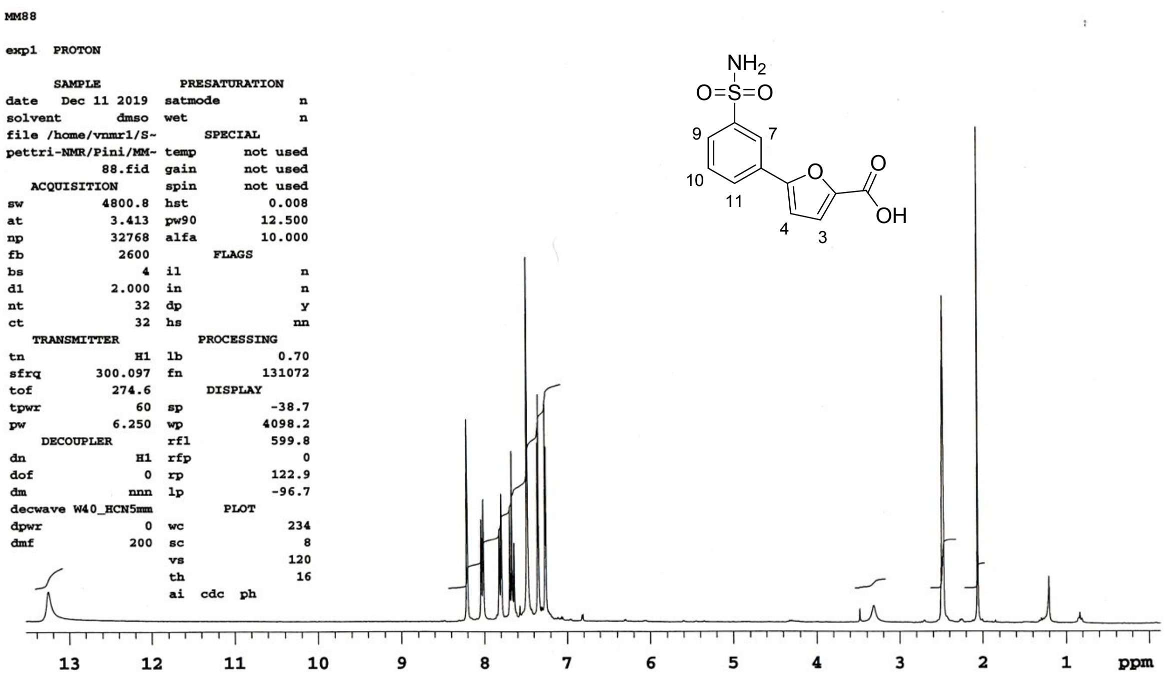

Figure S25. ^{1}H NMR spectrum of **8**.

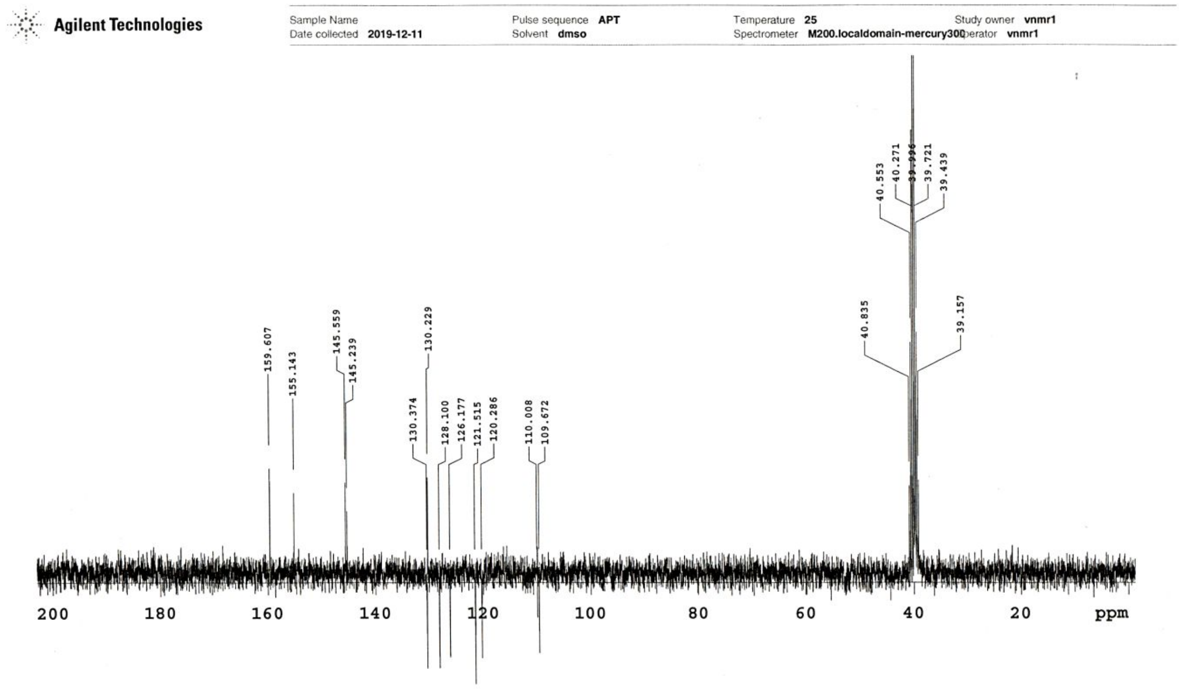

Figure S26. ^{13}C NMR spectrum of **8**.

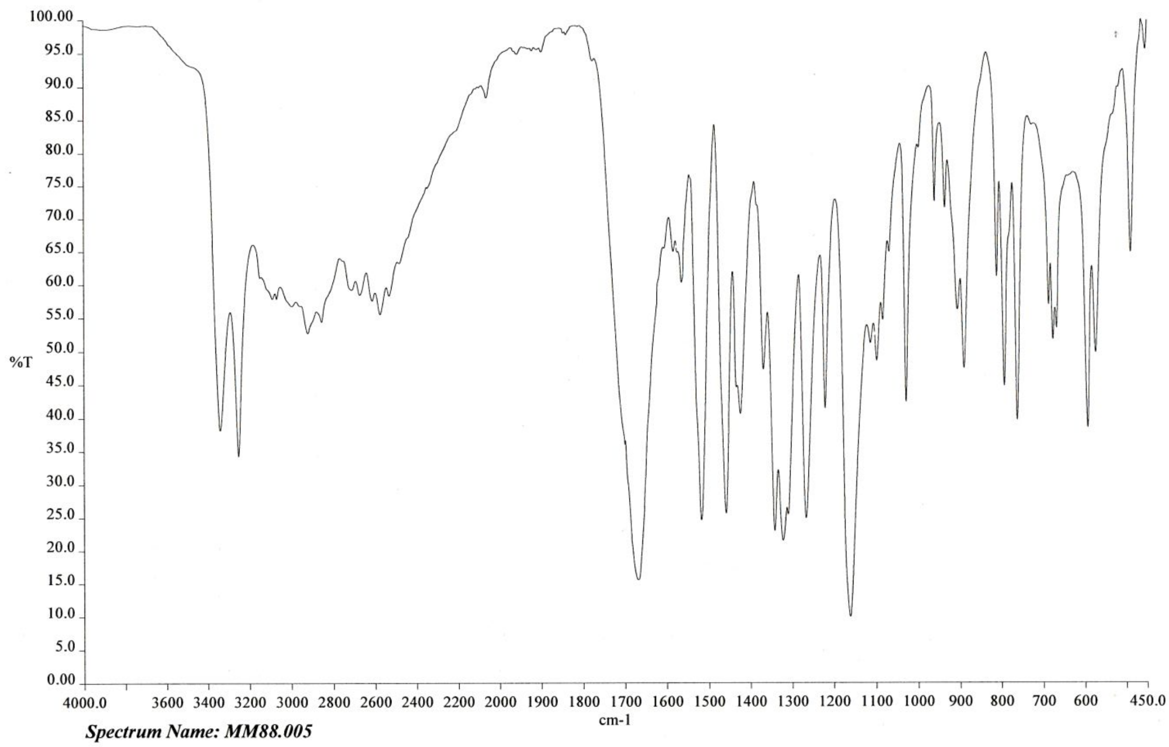

Figure S27. FT-IR spectrum of **8**.

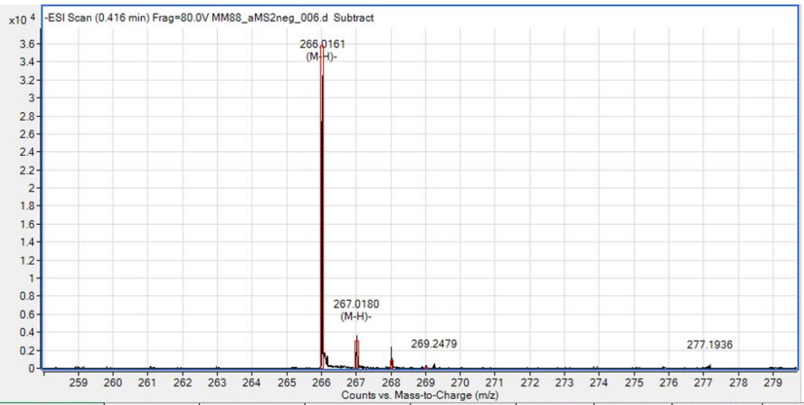

Best	Name	Formula	Score	Mass	Mass (Tgt)	Mass (DB)	Mass (MFG)	Diff (ppm)	Diff (abs. ppm)	Diff (mDa)	RT
TRUE		C11 H9 N O5 S	86.3	267.0222			267.0201	-7.55	7.55	-2.02	

Species	Ion Formula	m/z	Height	Score (MFG)	Score (MS)	Score (MFG, MS/MS	Score (mass)	Score (iso. abund)	Score (iso. spacing)
(M-H)-	C11 H8 N O5 S	266.0129	137009.9	86.3	86.3		71.61	99.4	99.97

m/z	m/z (Calc)	Diff (ppm)	Diff (mDa)	Height	Height (Calc)	Height %	Height % (Calc)	Height Sum %	Height Sum% (Calc)
266.0149	266.0129	-7.65	-2	138111.4	137009.9	100	100	83.7	83
267.0179	267.0158	-7.83	-2.1	17929.1	18269.8	13	13.3	10.9	11.1
268.0133	268.0114	-7.3	-2	7766.7	8662.5	5.6	6.3	4.7	5.2
269.0128	269.0138	3.84	1	1096.3	998.6	0.8	0.7	0.7	0.6
270.0153	270.0143	-3.56	-1	177.4	140	0.1	0.1	0.1	0.1

Figure S28. HRMS spectrum and data of **8**.

S2.8. 5-(3-Carboxyphenyl)furan-2-carboxylic acid (9)

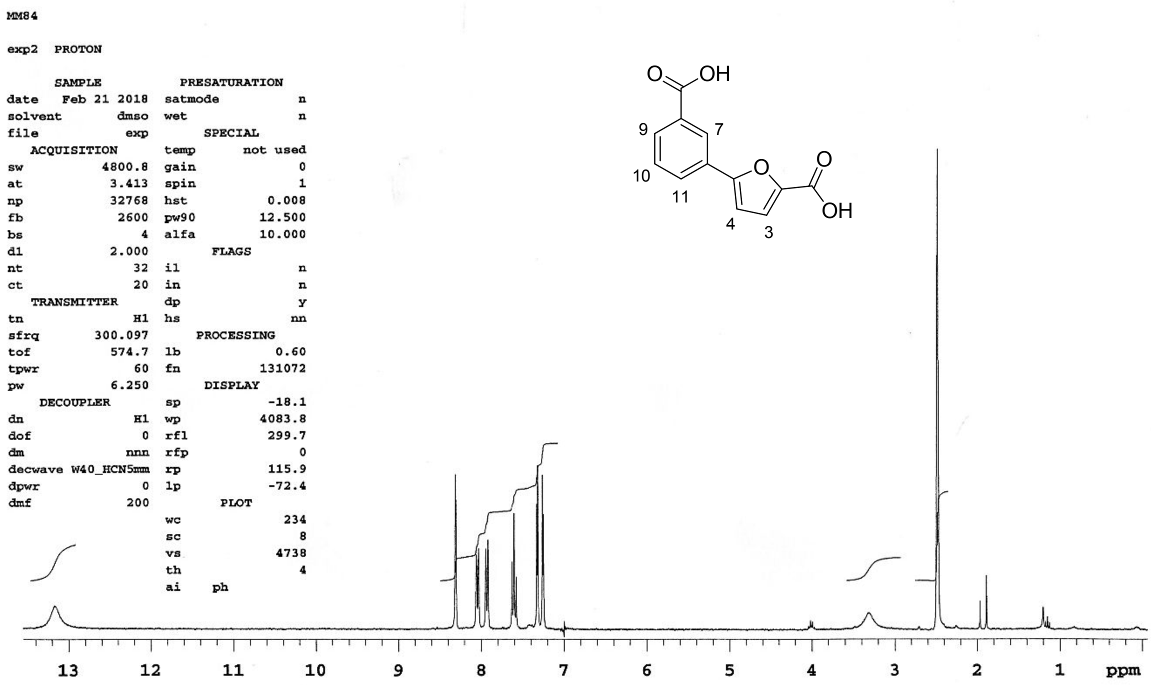

Figure S29. ^{1}H NMR spectrum of **9**.

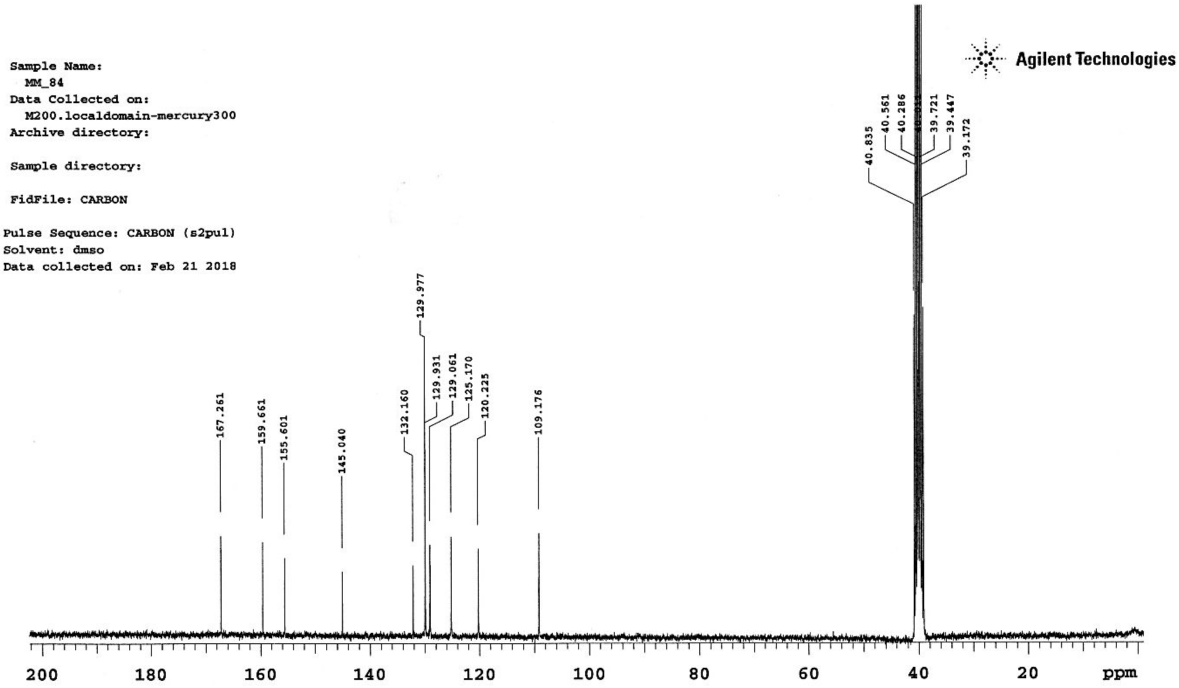

Figure S30. ^{13}C NMR spectrum of **9**.

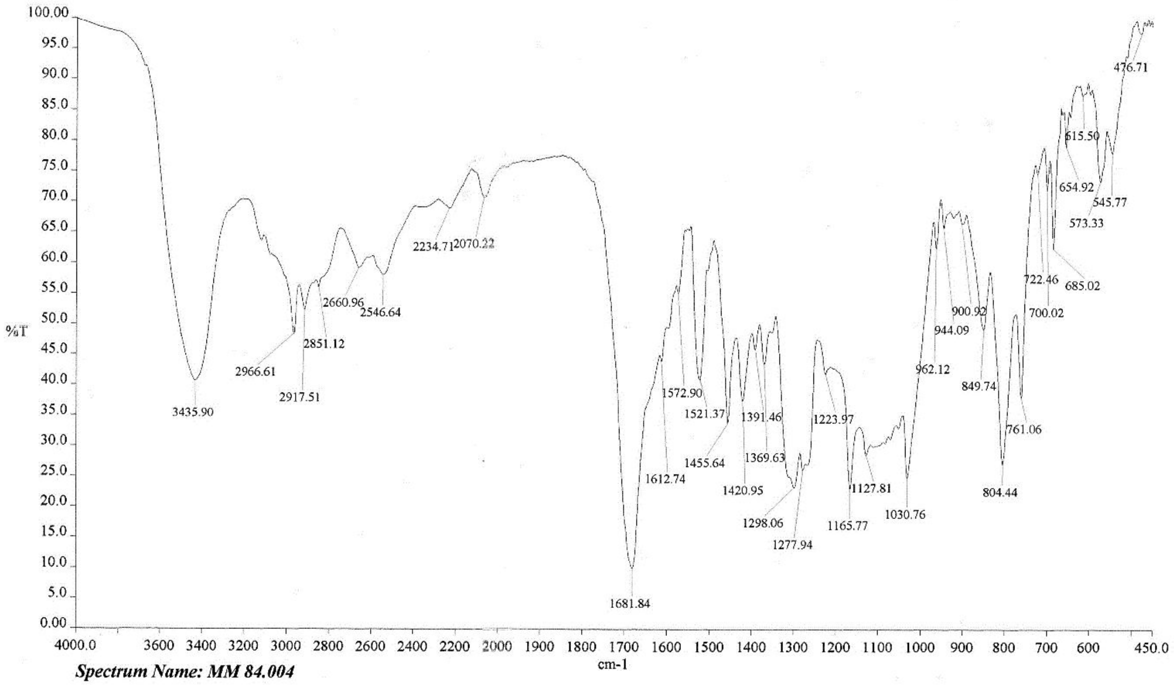

Figure S31. FT-IR spectrum of **9**.

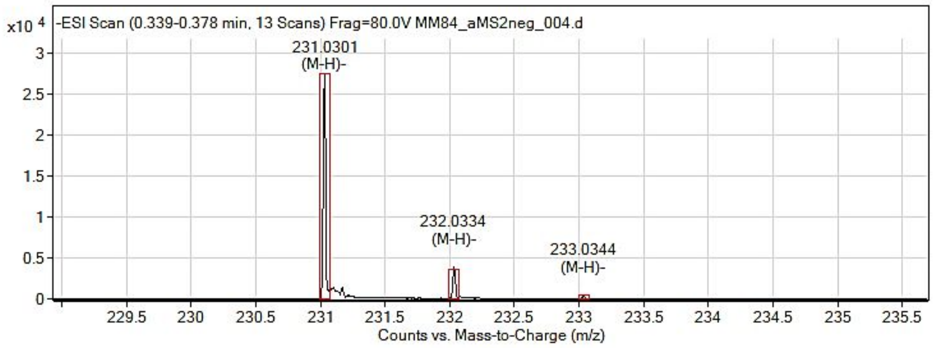

Best Name	Formula	Score	Mass	Mass (Tgt)	Mass (DB)	Mass (MFG)	Diff (ppm)	Diff (abs. ppm)	Diff (mDa)	RT
TRUE	C12 H8 O5	99.45	232.0374			232.0372	-0.79	0.79	-0.18	

Species	Ion Formula	m/z	Height	Score (MFG)	Score (MS)	Score (MFG, MS/MS)	Score (mass)	Score (iso. abund)	Score (iso. spacing)
(M-H)-	C12 H7 O5	231.0299	27465.2	99.45	99.45		99.69	98.75	99.82

m/z	m/z (Calc)	Diff (ppm)	Diff (mDa)	Height	Height (Calc)	Height %	Height % (Calc)	Height Sum %	Height Sum% (Calc)
231.0301	231.0299	-0.91	-0.2	27311.9	27465.2	100	100	86.4	86.9
232.0334	232.0333	-0.42	-0.1	3880.7	3639.1	14.2	13.2	12.3	11.5
233.0344	233.0353	3.61	0.8	415.7	504	1.5	1.8	1.3	1.6

Figure S32. HRMS spectrum and data of **9**.

S2.9. 5-(3-Cyanophenyl)furan-2-carboxylic acid (10)

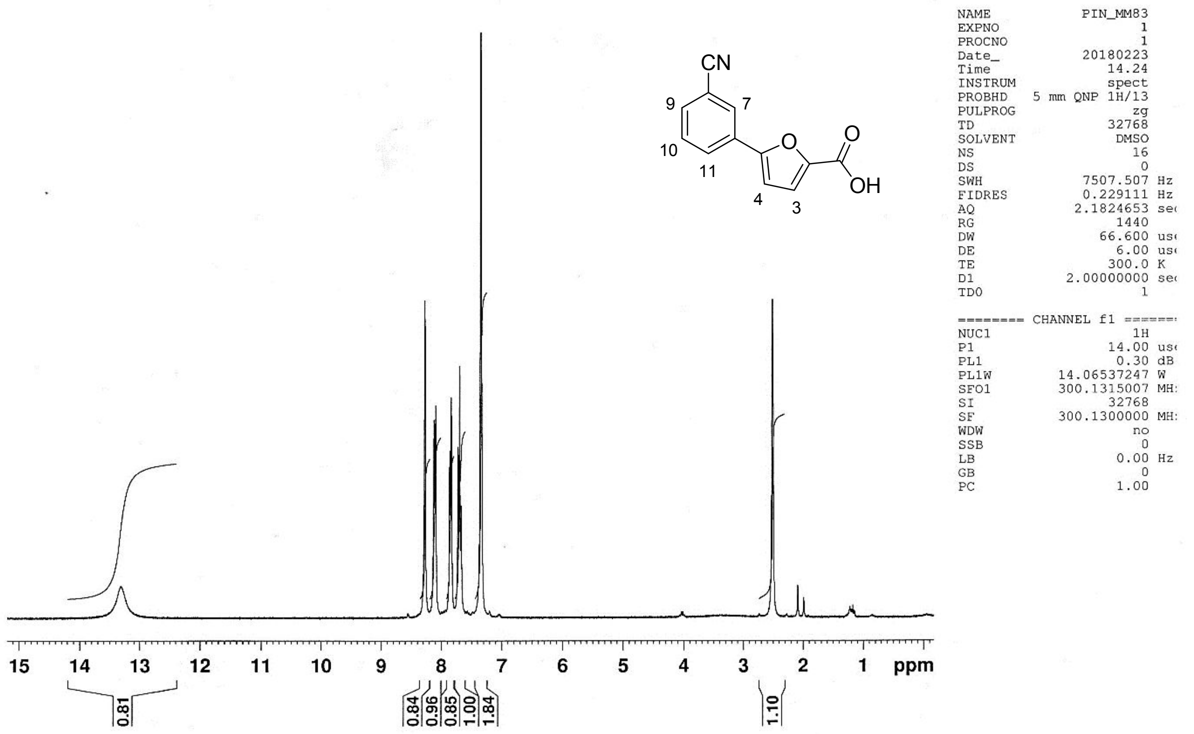

Figure S33. ^{1}H NMR spectrum of **10**.

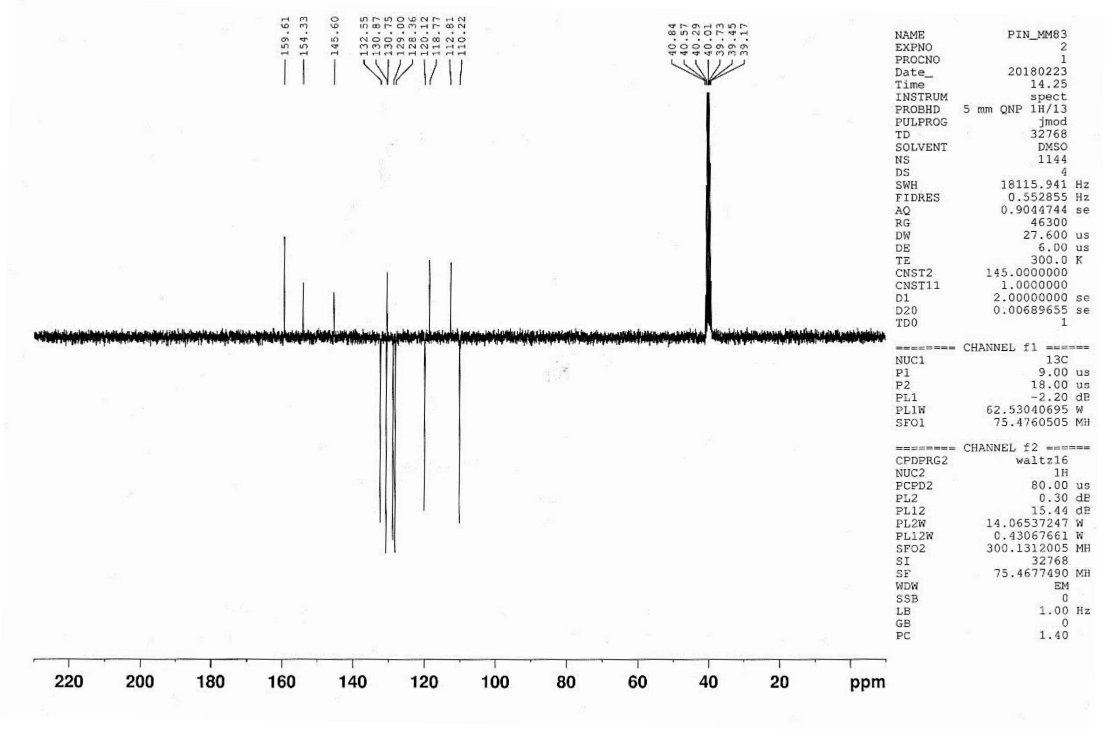

Figure S34. ^{13}C NMR spectrum of **10**.

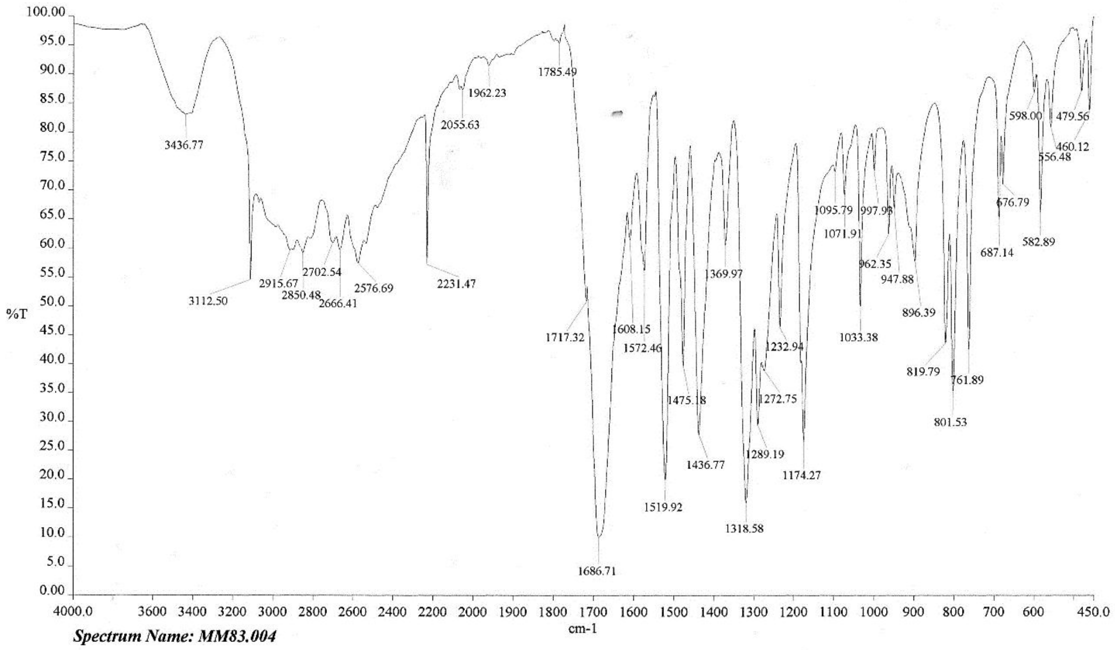

Figure S35. FT-IR spectrum of **10**.

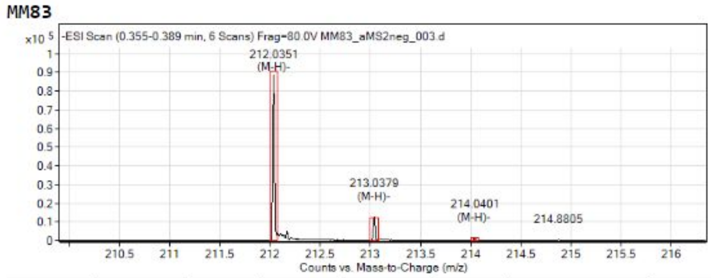

Best Name	Formula	Score	Mass	Mass (Tgt)	Mass (DB)	Mass (MFG)	Diff (ppm)	Diff (abs. ppm)	Diff (mDa)	RT
TRUE	C12 H7 N O3	99.21	213.0423			213.0426	1.27	1.27	0.27	

Species	Ion Formula	m/z	Height	Score (MFG)	Score (MS)	Score (MFG, MS/MS)	Score (mass)	Score (iso. abund)	Score (iso. spacing)
(M-H)-	C12 H6 N O3	212.0353	90642.5	99.21	99.21		99.29	99.53	98.68

m/z	m/z (Calc)	Diff (ppm)	Diff (mDa)	Height	Height (Calc)	Height %	Height % (Calc)	Height Sum %	Height Sum% (Calc)
212.0351	212.0353	1.01	0.2	90060.4	90642.5	100	100	86.4	87
213.0379	213.0385	2.91	0.6	12772	12261.7	14.2	13.5	12.3	11.8
214.0401	214.0408	3.17	0.7	1395.7	1323.9	1.5	1.5	1.3	1.3

Figure S36. HRMS spectrum and data of **10**.

S2.10. 5-(3-cyanophenyl)furan-2-carboxamide (11)

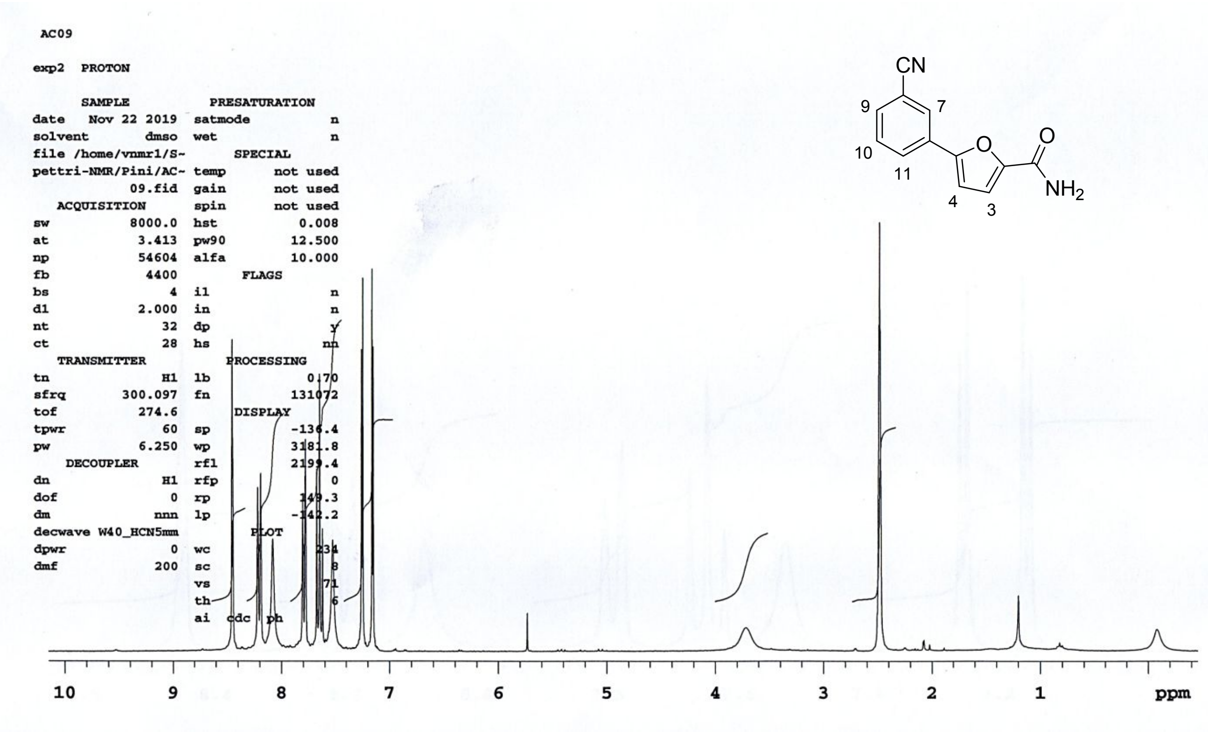

Figure S37. ^{1}H NMR spectrum of **11**.

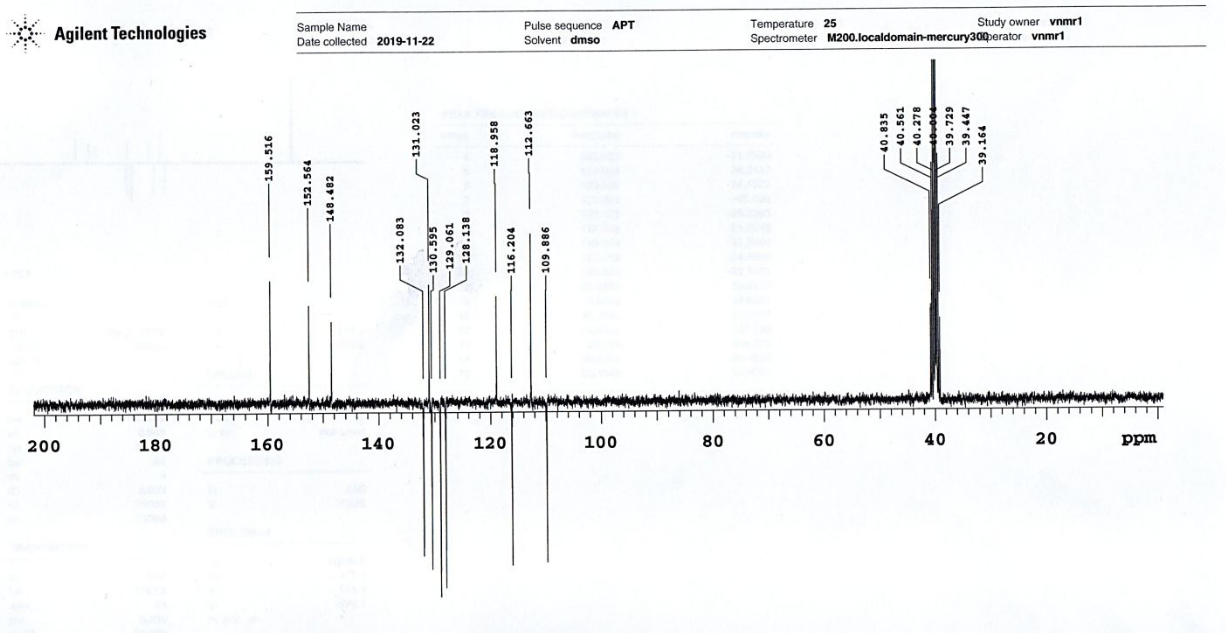

Figure S38. ^{13}C NMR spectrum of **11**.

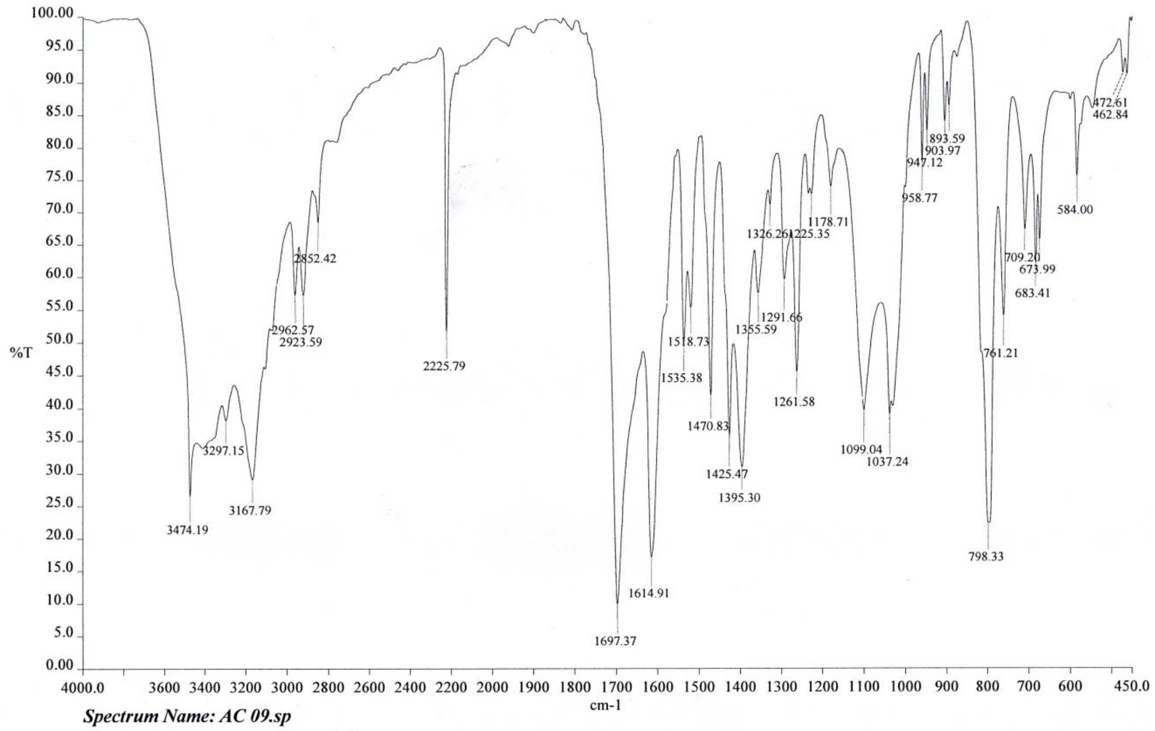

Figure S39. FT-IR spectrum of **11**.

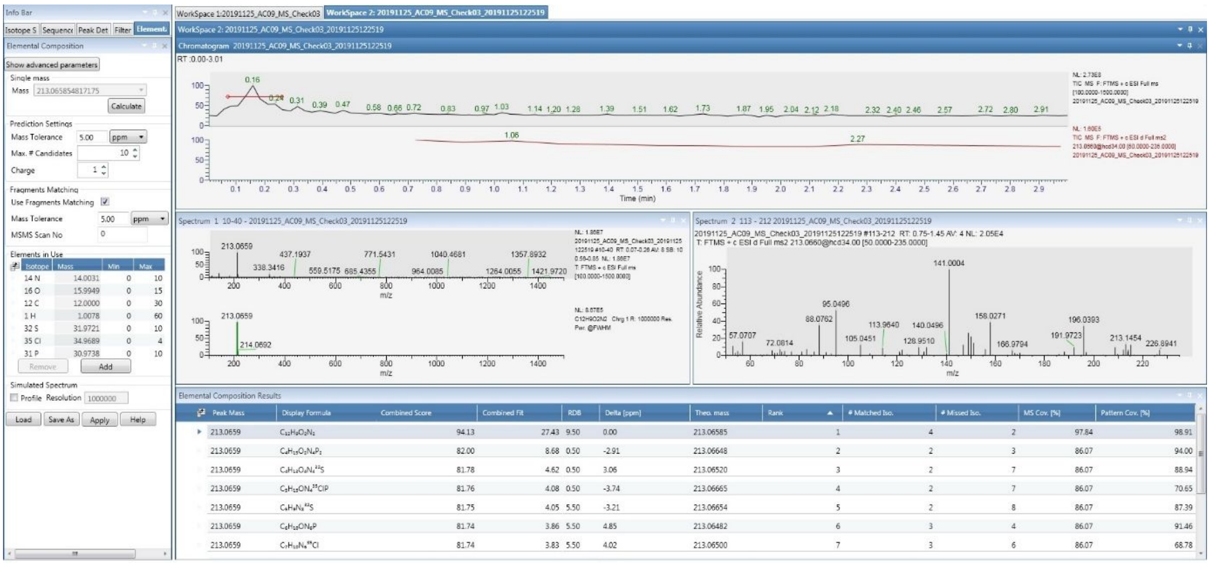

Figure S40. HRMS spectrum and data of **11**.

S2.11. Methyl 5-bromofuran-2-carboxylate (12)

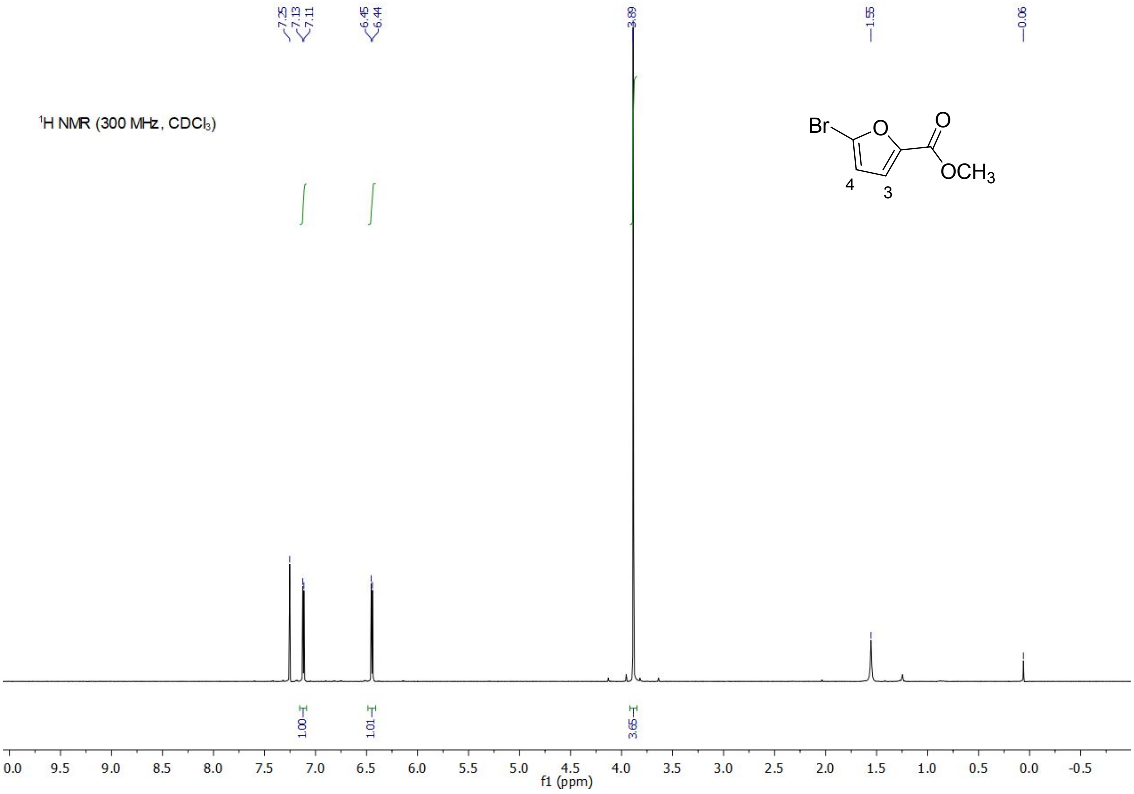

Figure S41. ^{1}H NMR spectrum of **12**.

S2.12. Methyl 5-(3-chlorophenyl)furan-2-carboxylate (13)

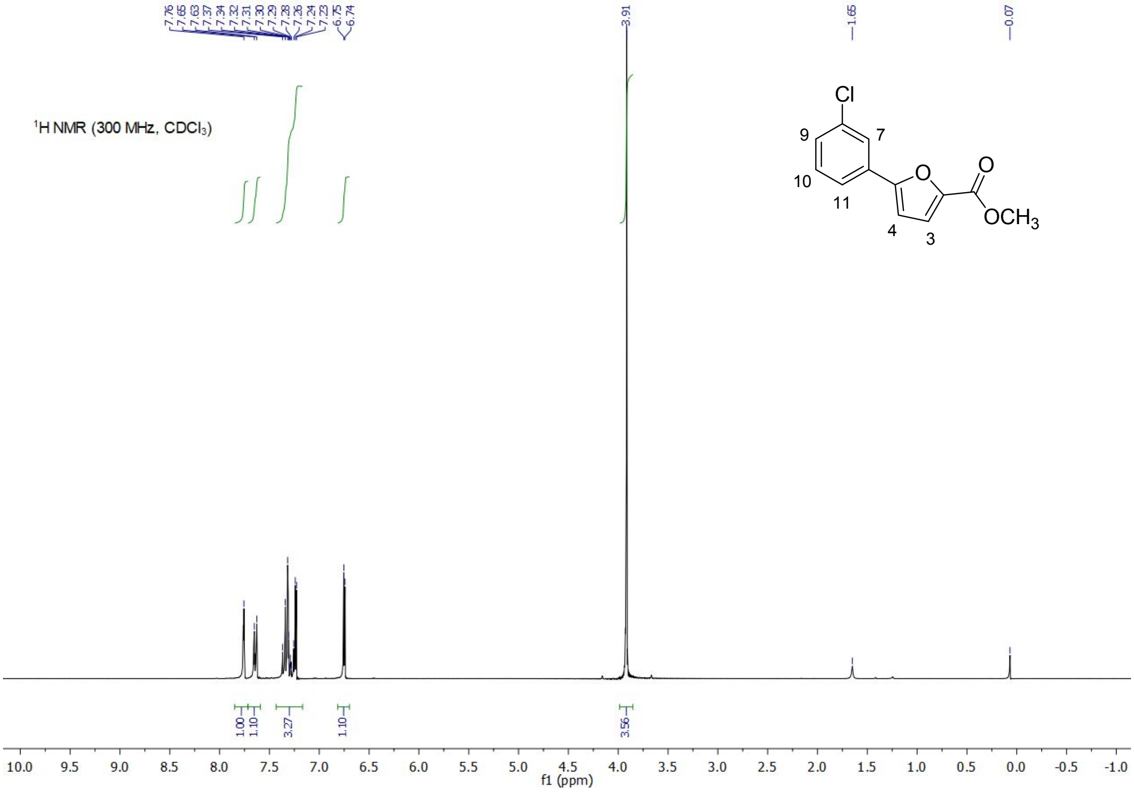

Figure S42. ^{1}H NMR spectrum of **13**.

S2.13. Methyl 5-(3-hydroxyphenyl)furan-2-carboxylate (14)

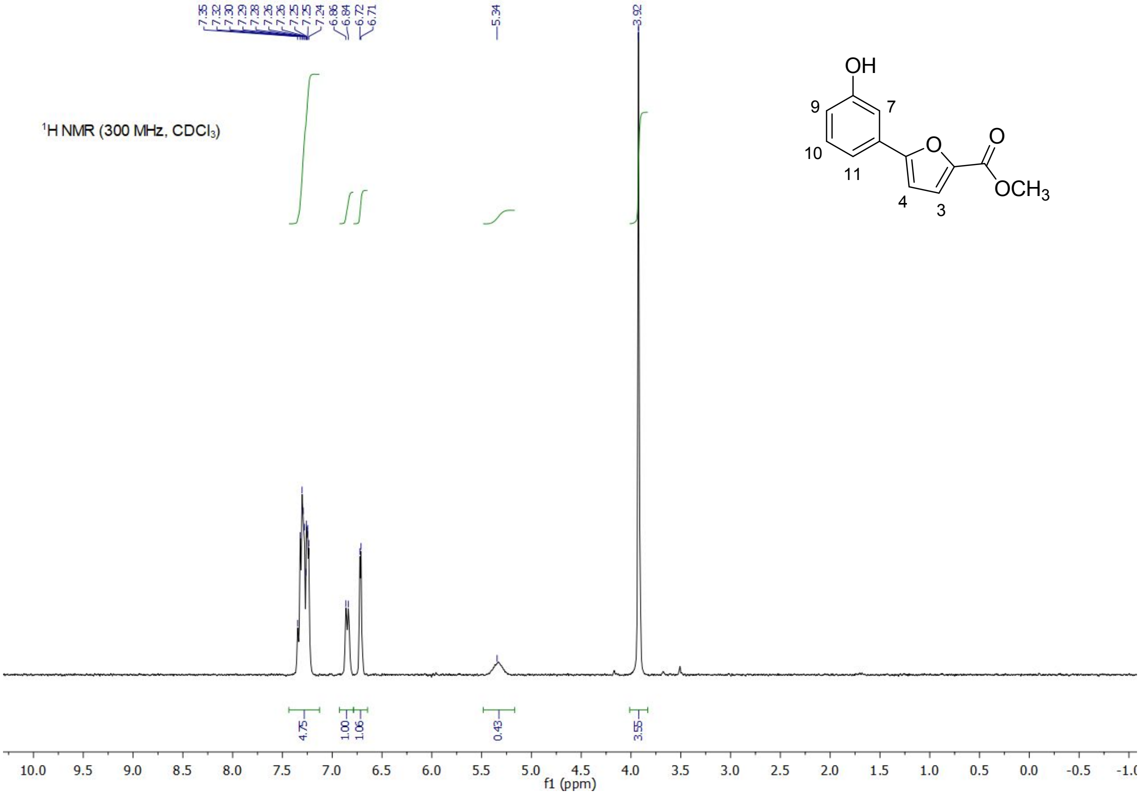

Figure S43. ^{1}H NMR spectrum of **14**.

S2.14. Methyl 5-(3-carbamoylphenyl)furan-2-carboxylate (15)

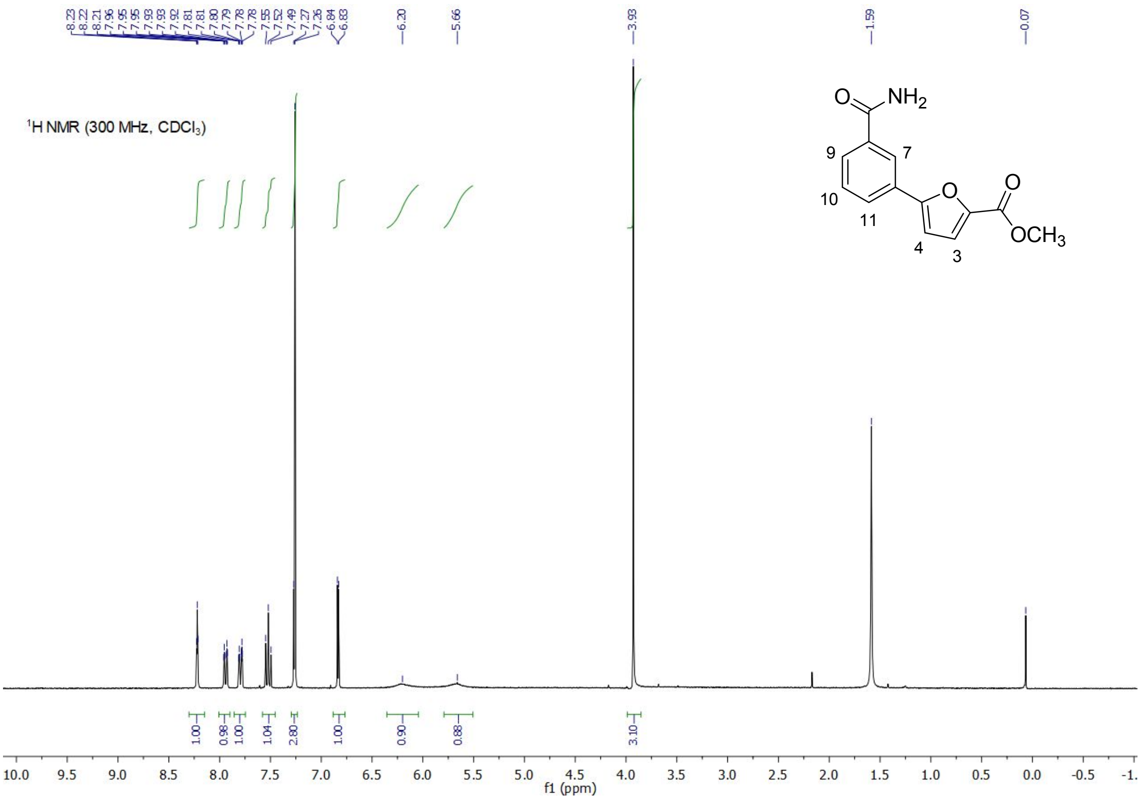

Figure S44. ^{1}H NMR spectrum of **15**.

S2.15. Methyl 5-(3-cyanophenyl)furan-2-carboxylate (16)

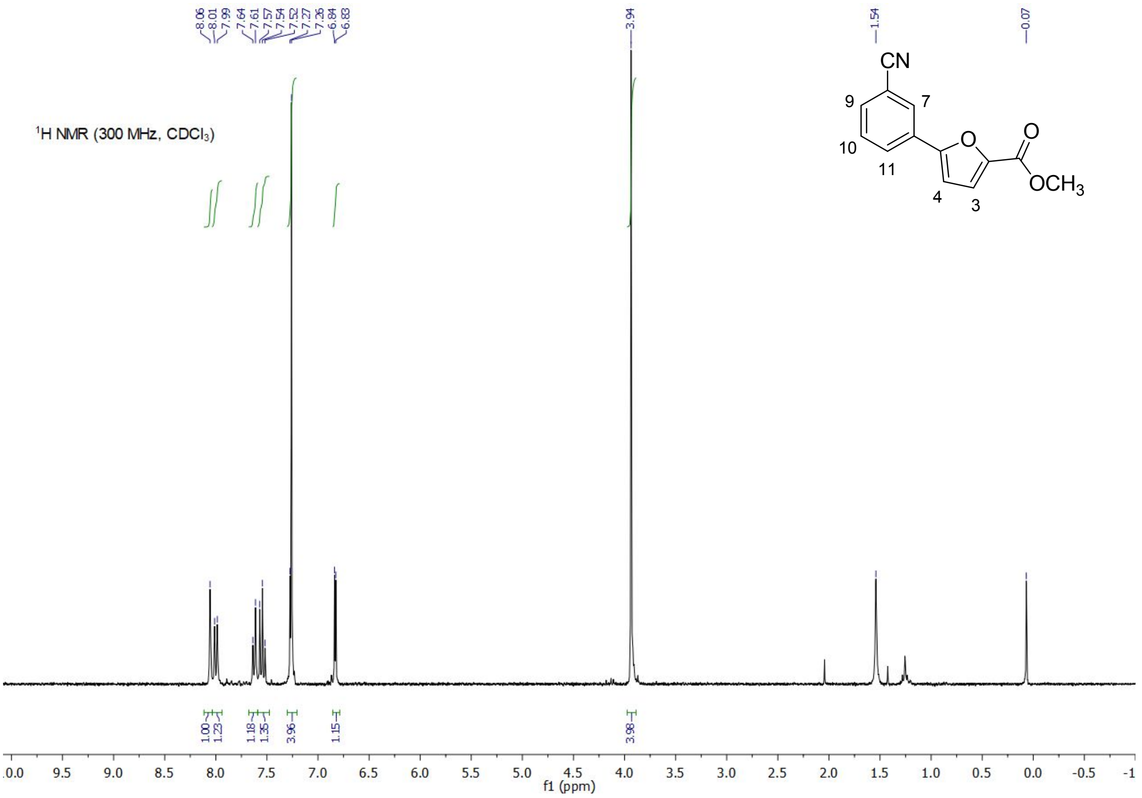

Figure S45. ^{1}H NMR spectrum of **16**.

S2.16. (5-(methoxycarbonyl)furan-2-yl)boronic acid (17)

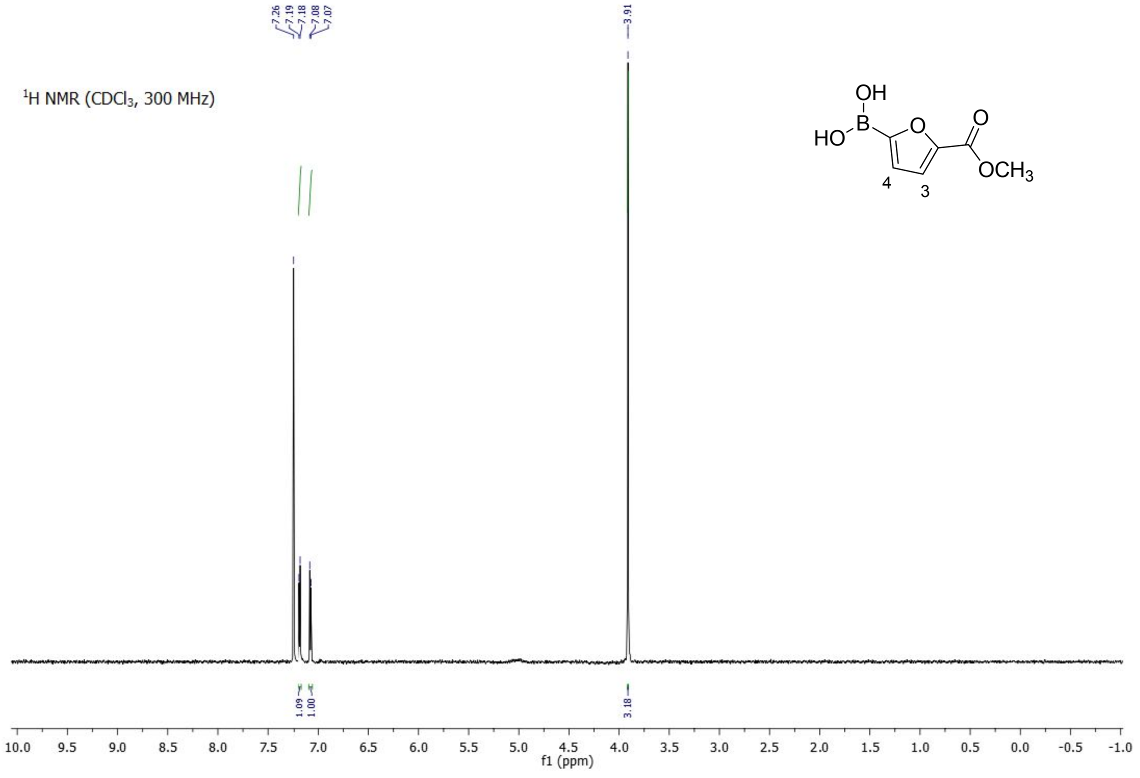

Figure S46. ^{1}H NMR spectrum of **17**.

S2.17. Methyl 5-(*m*-tolyl)furan-2-carboxylate (18)

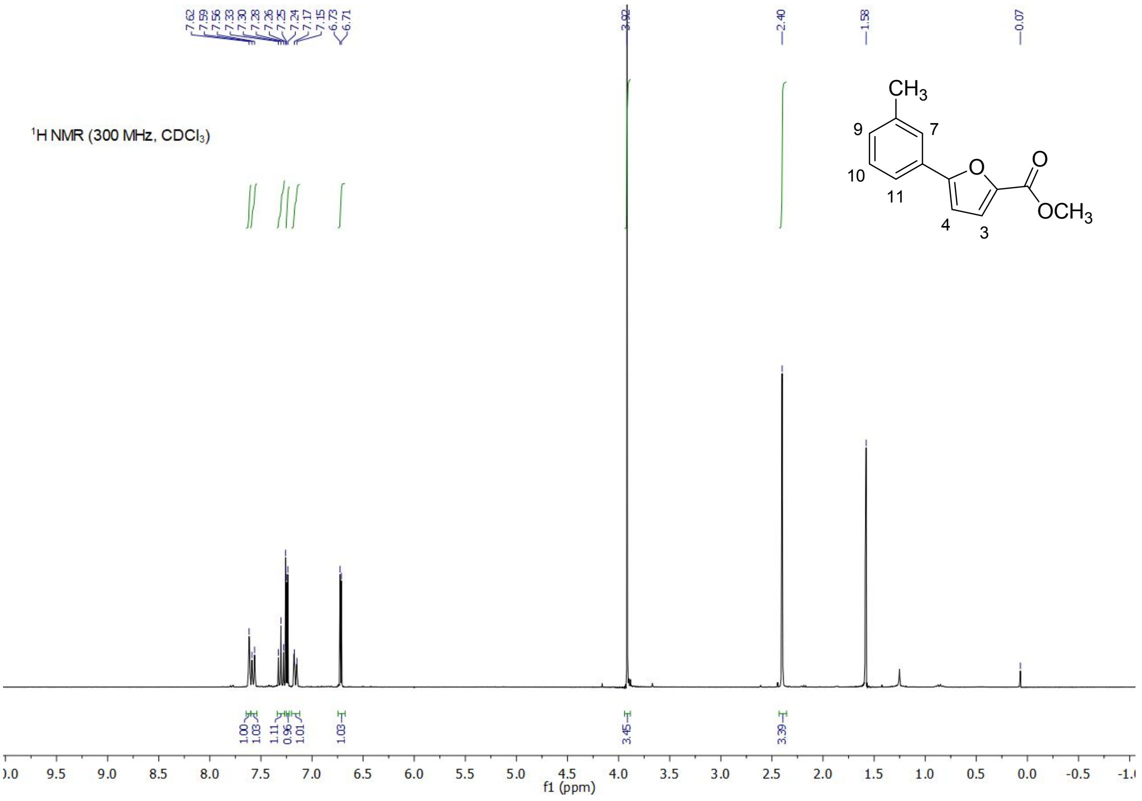

Figure S47. ^{1}H NMR spectrum of **18**.

S2.18. Methyl 5-(3-(methylcarbamoyl)phenyl)furan-2-carboxylate (19)

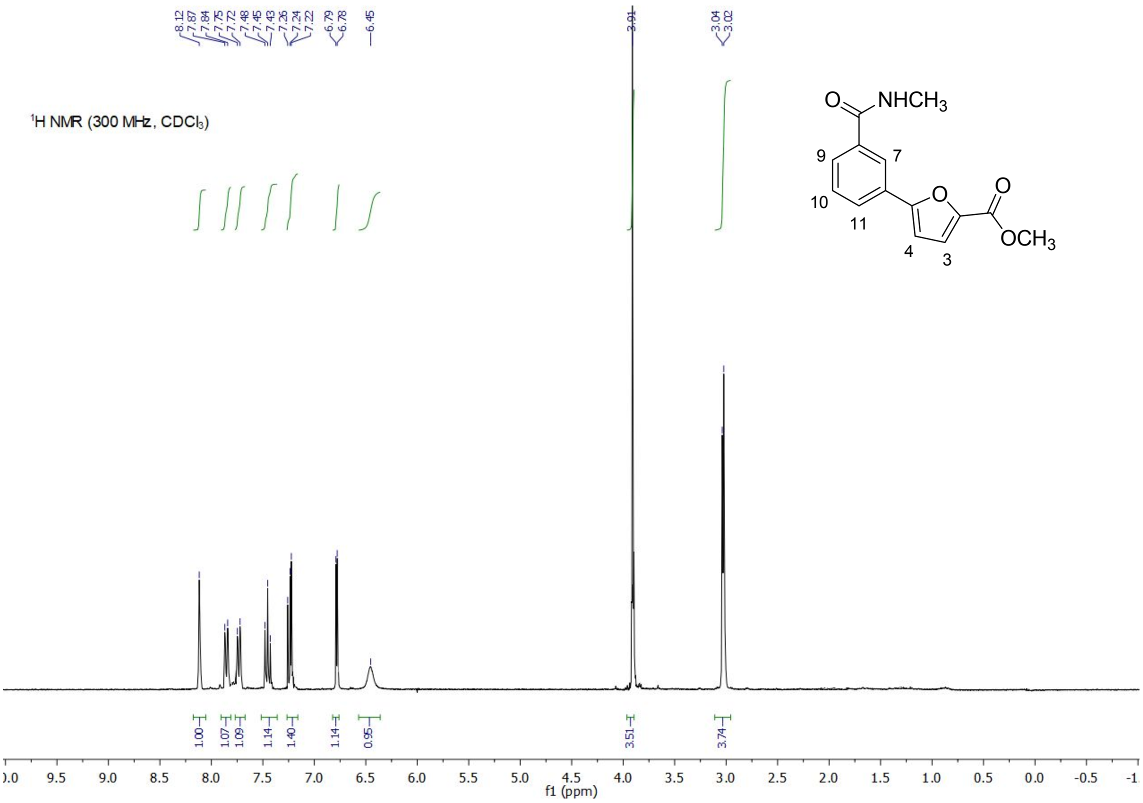

Figure S48. ^{1}H NMR spectrum of **19**.

S2.19. Methyl 5-(3-sulfamoylphenyl)furan-2-carboxylate (20)

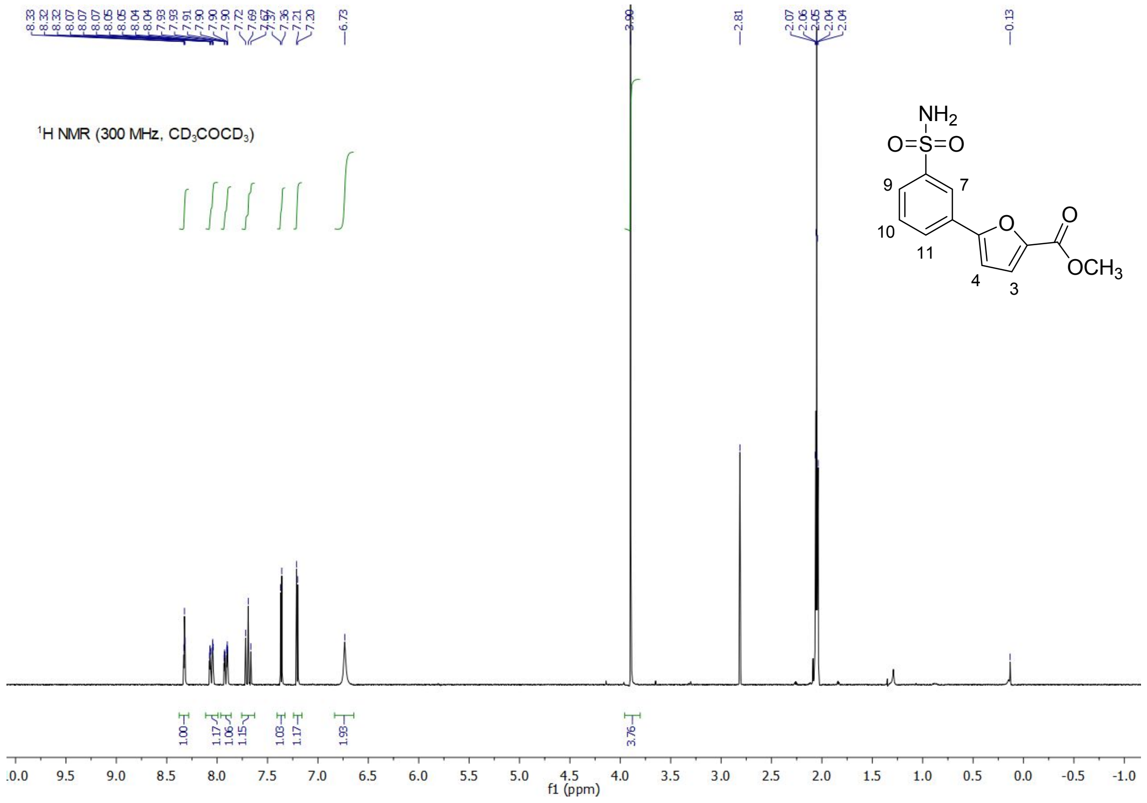

Figure S49. ^{1}H NMR spectrum of **20**.

S2.20. Methyl 5-(3-(methoxycarbonyl)phenyl)furan-2-carboxylate (21)

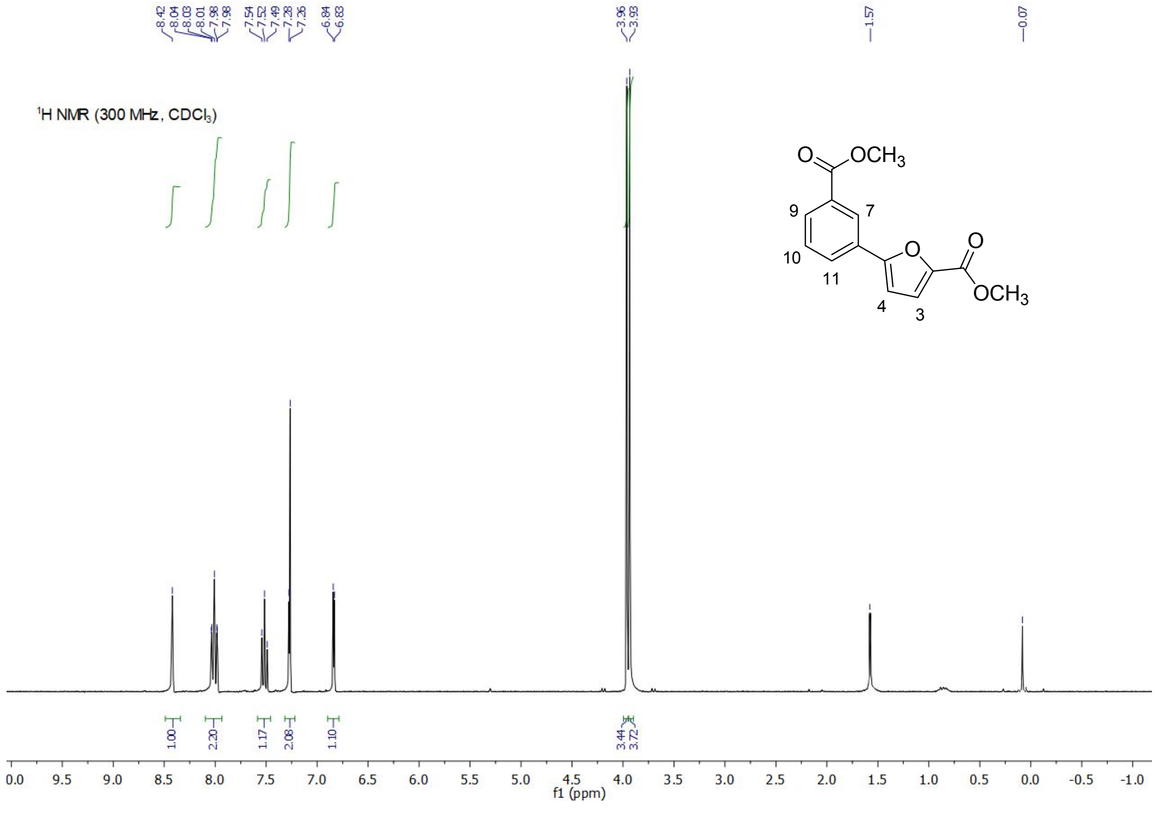

Figure S50. ^{1}H NMR spectrum of **21**.

S2.21. Methyl 5-(3-aminophenyl)furan-2-carboxylate (22)

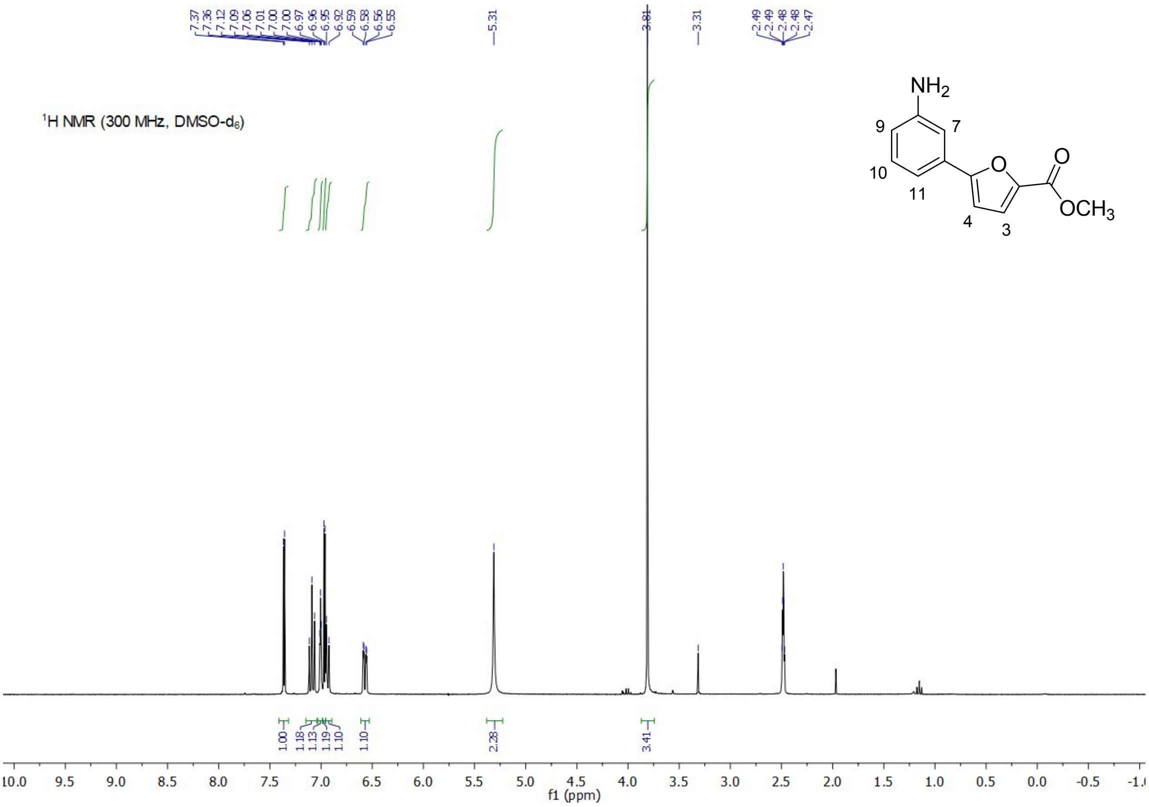

Figure S51. ^{1}H NMR spectrum of **22**.

S2.22. Methyl 5-(3-nitrophenyl)furan-2-carboxylate (23)

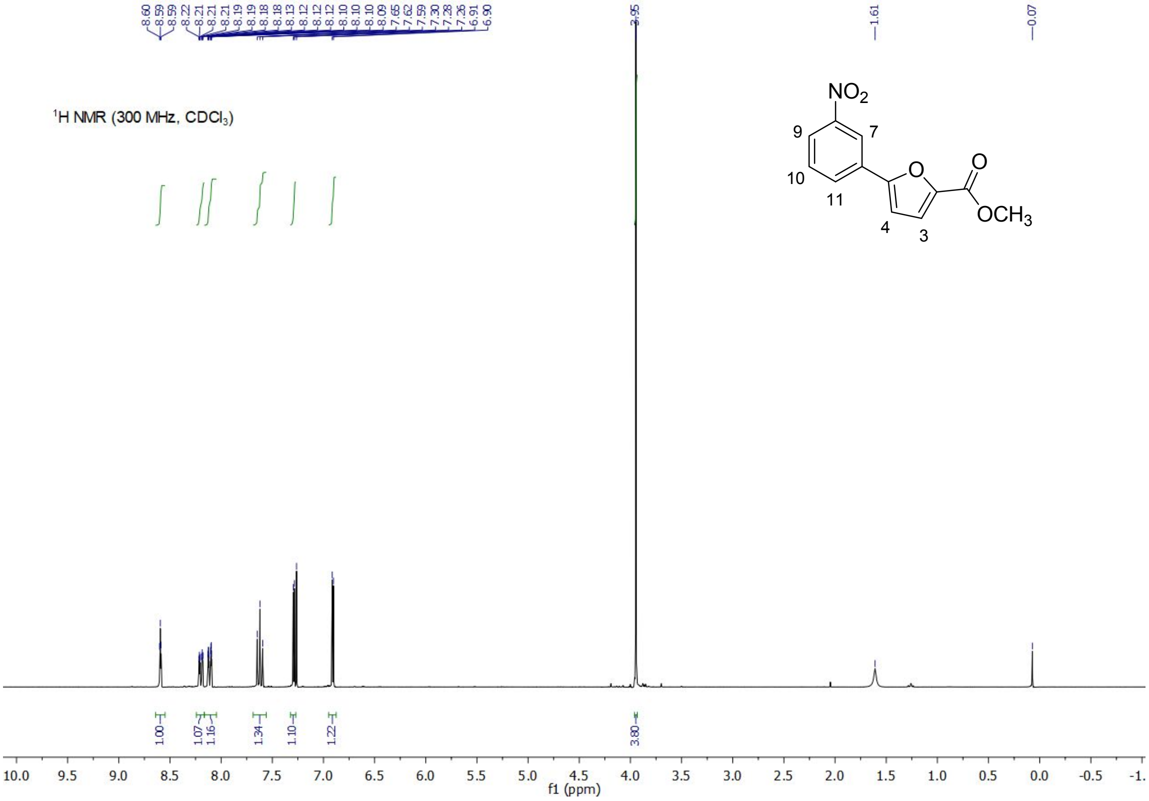

Figure S52. ^{1}H NMR spectrum of **23**.

S2.23. 3-Bromo-*N*-methylbenzamide (24)

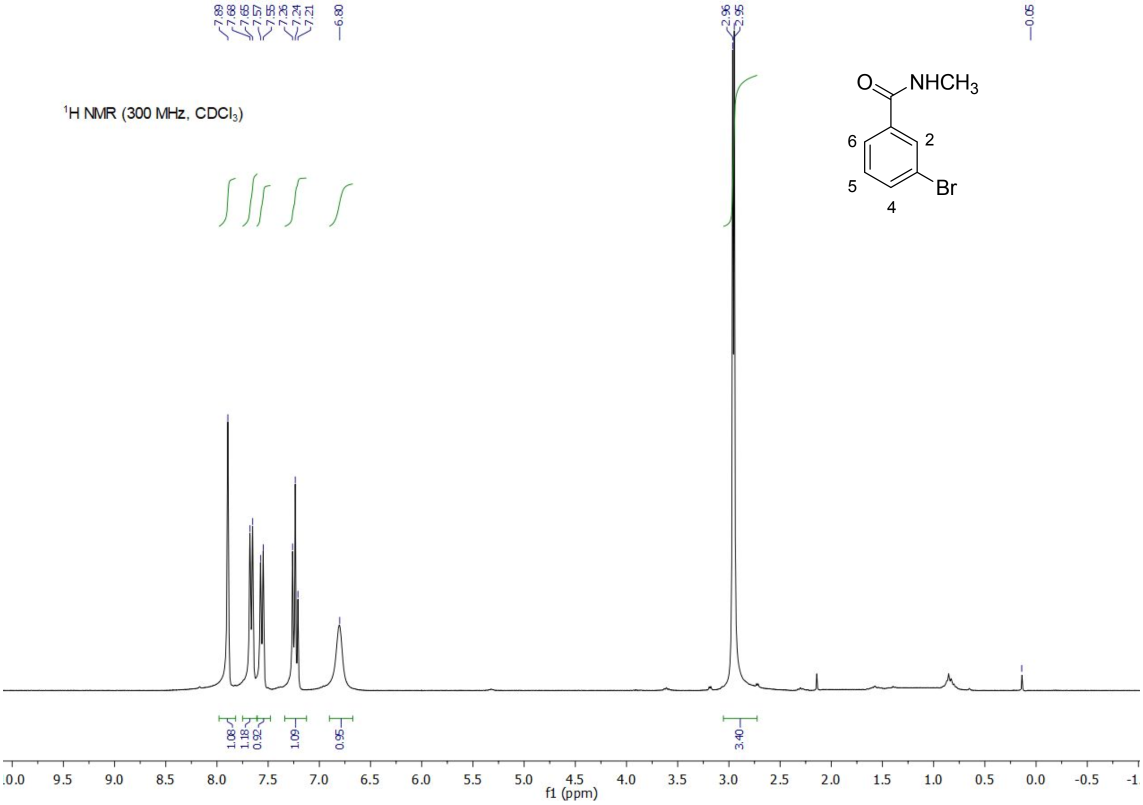

Figure S53. ^{1}H NMR spectrum of **24**.

S3. Crystal structures

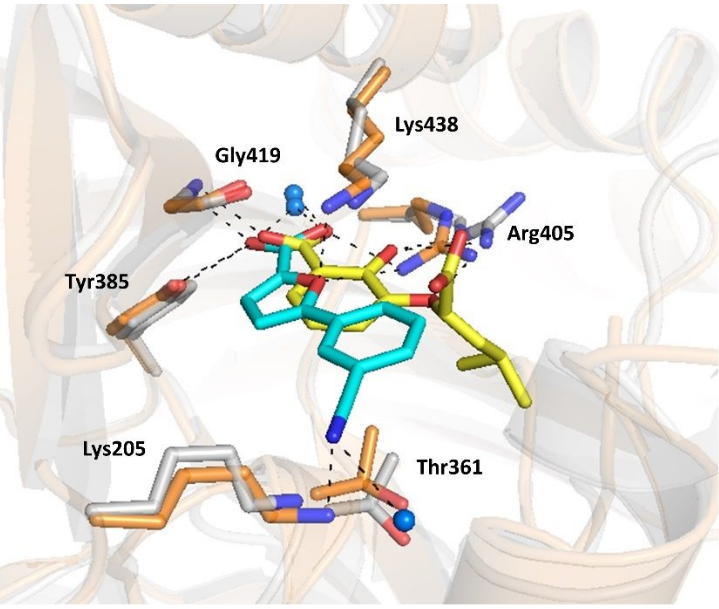

Figure S54. Superposition of the ribbon diagrams of MbtI-**10** (orange, PDB ID 6ZA4) and PDB ID 3RV7 (grey): the interactions of the ligand with the side chains (in sticks) are represented as dashed lines, and the water molecules as blue spheres.

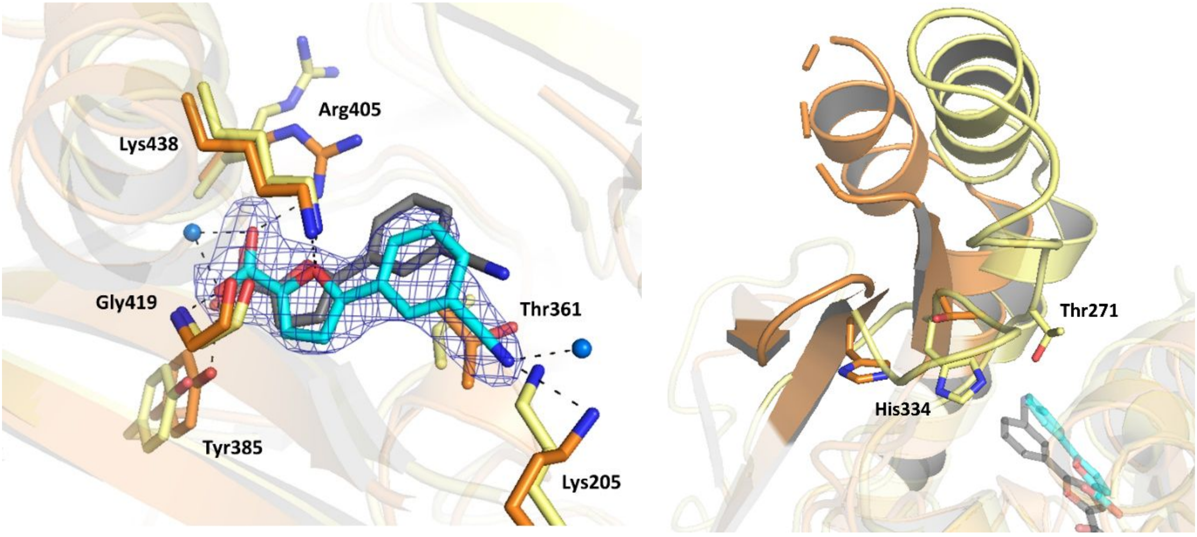

Figure S55. On the left, the superposition of the ribbon diagram of MbtI-**10** (orange, PDB ID 6ZA4) and the computational model (PDB ID 3VEH, light yellow): the interactions of the ligand with the side chains (in sticks) and the water molecules (blue spheres) are represented as dashed lines. The blue mesh represents the electron density around the ligand (contoured at 1σ). On the right, the different position of Thr271 and His334 is highlighted.

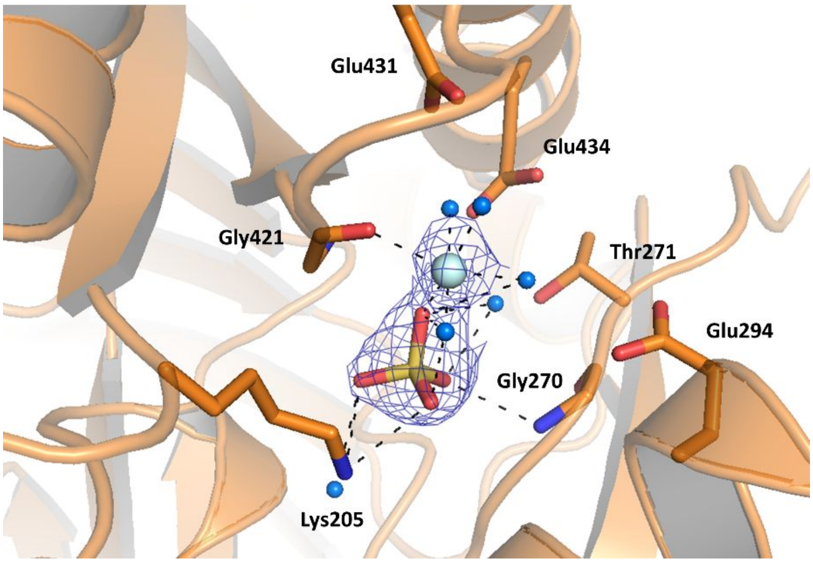

Figure S56. Ribbon diagram of the MbtI-Mg^{2+} structure (PDB ID 6ZA5), focused on the active site of chain A. The interactions of the ligands with the side chains (in sticks), the Mg^{2+} ion (light blue sphere) and the water molecules (blue spheres) are represented as dashed lines. The blue mesh represents the electron density around the ligand (contoured at 1σ).

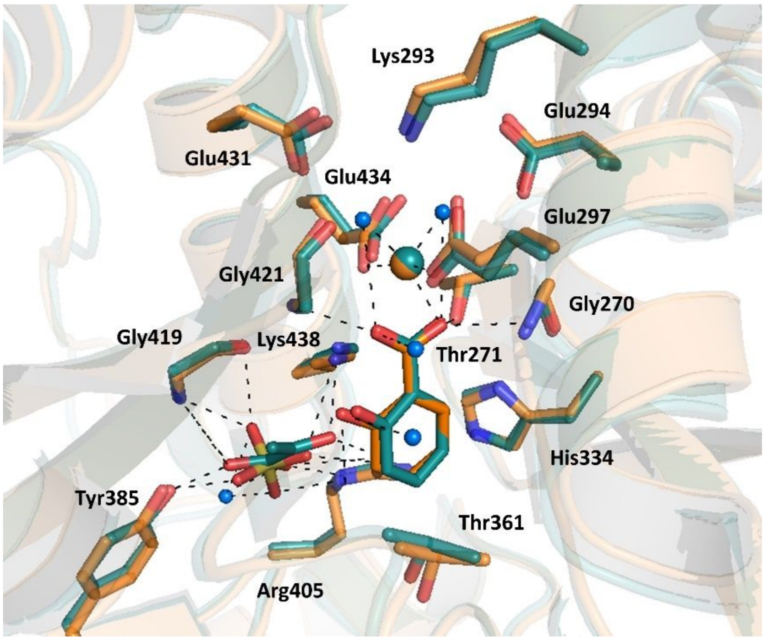

Figure S57. Overlay of MbtI-Mg^{2+} (orange, PDB ID 6ZA5) with Irp9 from *Yersinia enterocolitica* (PDB ID 2FN1, teal). The interactions between the ligands and the side chains (in sticks) and the water molecules (as blue spheres) are represented as dashed lines. For the sake of clarity, the amino acid numbering refers to MbtI.

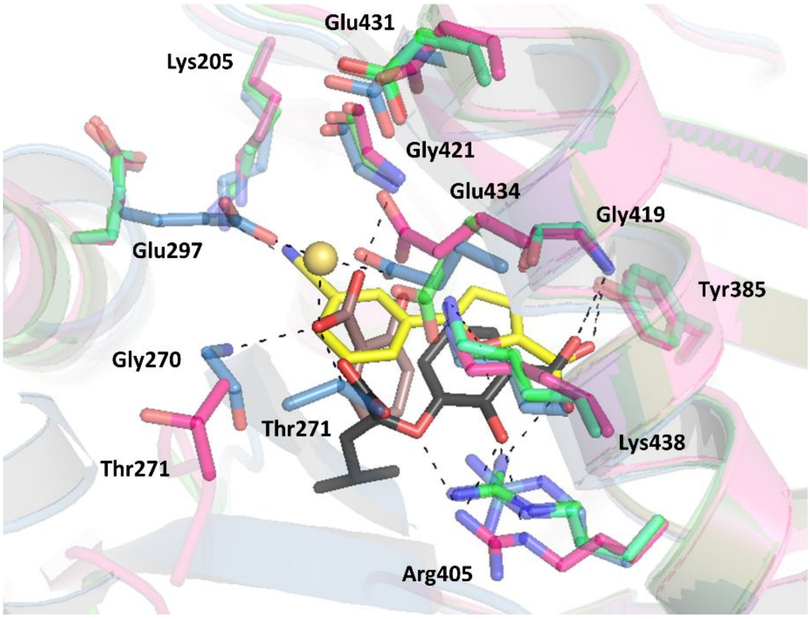

Figure S58. Superposition of the ribbon diagram of MbtI-Mg^{2+} (blue, PDB ID 6ZA5), MbtI-**10** (green, PDB ID 6ZA4), PDB ID 3RV7 (magenta), evidencing the rearrangement of the side chains in the active site. The interactions between the ligands and the side chains (in sticks) are represented as dashed lines; the Mg^{2+} ion is represented as a light-yellow sphere. The ligands are represented with different colors, old rose for salicylate, yellow for **10**, and black for the 3RV7-inhibitor.

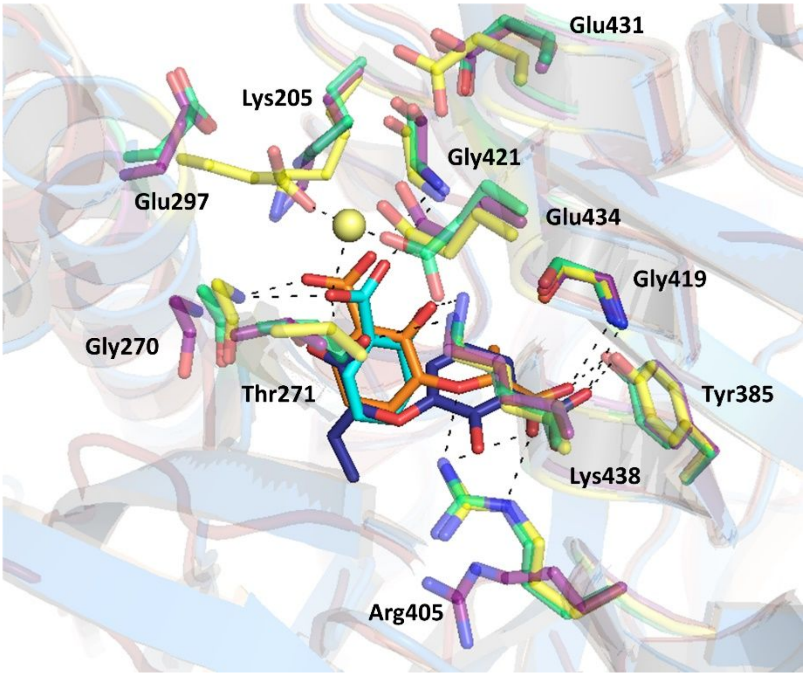

Figure S59. Superposition of the ribbon diagram of MbtI-Mg^{2+} (yellow, PDB ID 6ZA5), PDB ID 3VEH (purple), PDB ID 3ST6 (green), evidencing the rearrangement of the side chains in the active site. The interactions between the ligands and the side chains (in sticks) are represented as dashed lines; the Mg^{2+} ion is represented as a light-yellow sphere. The ligands are represented with different colors, cyan for salicylate, blue for 3VEH-inhibitor, and orange for the 3ST6-inhibitor.

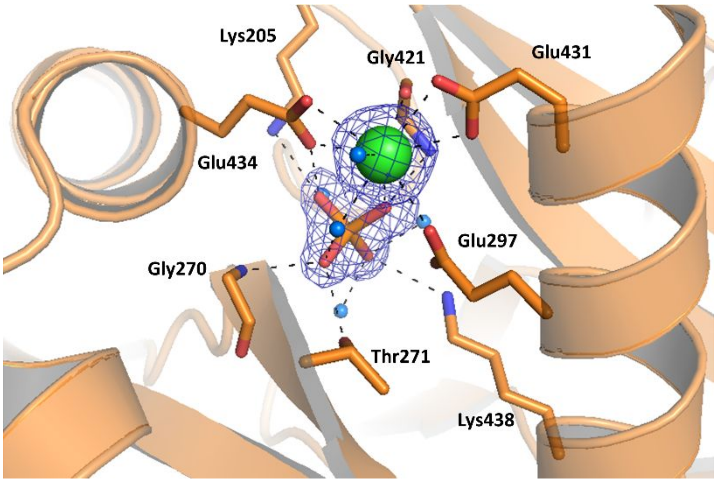

Figure S60. Ribbon diagram of the MbtI-Ba^{2+} structure (PDB ID 6ZA6): the interactions of Ba^{2+} (green sphere) and its phosphate counterion with the side chains (in sticks) and the water molecules (blue spheres) are represented as dashed lines. The blue mesh represents the electron density around the ions (contoured at 1σ).

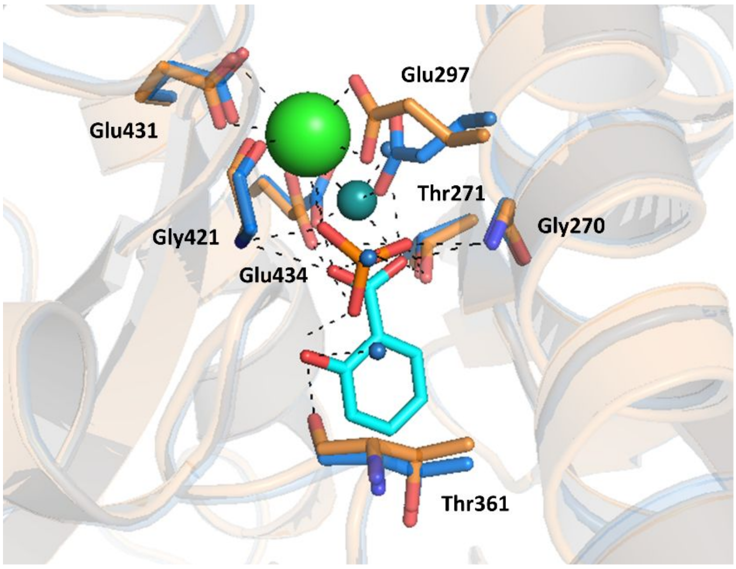

Figure 61. Overlay of MbtI-Ba^{2+} (orange, PDB ID 6ZA6) with MbtI-Mg^{2+} (blue, PDB ID 6ZA5), with Ba^{2+} and Mg^{2+} ions as green and teal spheres, respectively. The interactions between the ligands and the side chains (in sticks) are represented as dashed lines.

S4. Computational simulation

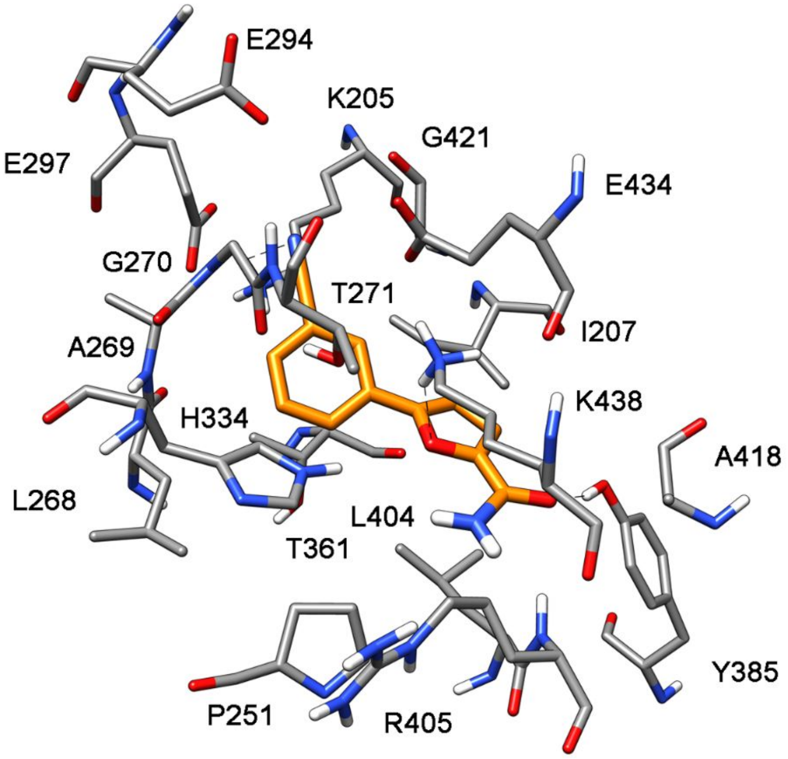

Figure S62. Simulated binding mode of compound **11** within MbtI (PDB ID 3VEH).

S5. Mass spectrometry analysis

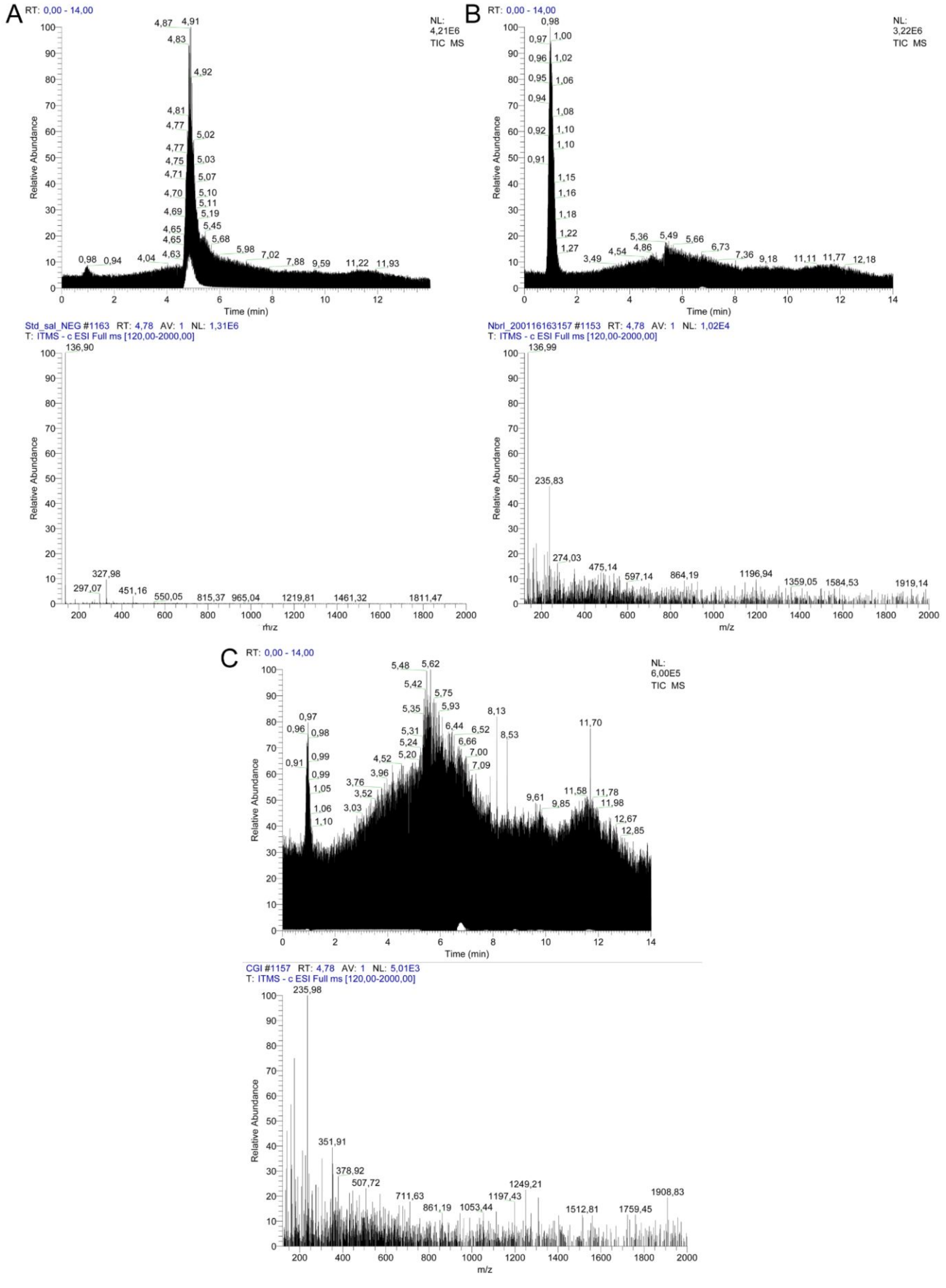

Figure S63. UPLC-MS profile of the supernatant of the denatured MbtI confirmed the presence of a peak with m/z=137, corresponding to the salicylic acid bound to the recombinant protein. A) Profile of the standard of salicylic acid; B) profile of the supernatant of the MbtI solution after heat denaturation;

C) profile of the negative control, pantothenate kinase, expressed, purified and treated in the same manner of MbtI.

S6. SMILES codes

COMPOUND	SMILES code
I	OC(C(O1)=CC=C1C2=CC=C([N+]([O-])=O)C=C2)=O
II	OC(C(O1)=CC=C1C2=CC=C(C(F)(F)F)C=C2C(F)(F)F)=O
1	OC(C(O1)=CC=C1C2=CC=CC(C(F)(F)F)=C2)=O
2	OC(C(O1)=CC=C1C2=CC=CC(Cl)=C2)=O
3	OC(C(O1)=CC=C1C2=CC=CC(O)=C2)=O
4	OC(C(O1)=CC=C1C2=CC=CC(C)=C2)=O
5	OC(C(O1)=CC=C1C2=CC=CC(N)=C2)=O
6	OC(C(O1)=CC=C1C2=CC=CC(C(N)=O)=C2)=O
7	OC(C(O1)=CC=C1C2=CC=CC(C(NC)=O)=C2)=O
8	OC(C(O1)=CC=C1C2=CC=CC(S(=O)(N)=O)=C2)=O
9	OC(C(O1)=CC=C1C2=CC=CC(C(O)=O)=C2)=O
10	OC(C(O1)=CC=C1C2=CC=CC(C#N)=C2)=O
11	NC(C(O1)=CC=C1C2=CC=CC(C#N)=C2)=O
